# Supplementary material for: Analysis of inter-hospital transfer on clinical outcomes after primary percutaneous coronary intervention for ST-segment elevation myocardial infarction: A secondary analysis of the BRIGHT-4 trial
Source: PLoS Med. 2025 Jul 23;22(7):e1004679. doi: 10.1371/journal.pmed.1004679 (PMC12313069; doi:10.1371/journal.pmed.1004679)
Supplement: S1 Table — (DOCX) [file pmed.1004679.s001.docx]

S1 Table. Medications and procedural data

|  | **Inter-hospital transfer (N=2121)** | **Direct admission**  **(N=3817)** | ***P* Value** |
| --- | --- | --- | --- |
| Chronic oral anticoagulant | 45 (2.1%) | 98 (2.6%) | 0.28 |
| Warfarin | 13 (0.6%) | 31 (0.8%) | 0.39 |
| Direct-acting oral anticoagulant | 32 (1.5%) | 67 (1.8%) | 0.48 |
| Beta-blocker | 1611 (76.0%) | 2901 (76.0%) | 0.97 |
| Calcium channel blocker | 167 (7.9%) | 281 (7.4%) | 0.47 |
| ACEI/ARB/ARNI | 1233 (58.1%) | 2180 (57.1%) | 0.45 |
| Diuretic | 1269 (59.8%) | 2220 (58.2%) | 0.21 |
| Any lipid-lowering agent | 2000 (94.3%) | 3637 (95.3%) | 0.10 |
| Statin | 1911 (90.1%) | 3460 (90.6%) | 0.49 |
| Other lipid-lowering agent | 103 (4.9%) | 188 (4.9%) | 0.91 |

Data are shown as n (%). ACEI, angiotensin converting enzyme inhibitor. ARB, angiotensin receptor blocker. ARNI, angiotensin receptor-neprilysin inhibitor.
