# Supplementary material for: Analysis of inter-hospital transfer on clinical outcomes after primary percutaneous coronary intervention for ST-segment elevation myocardial infarction: A secondary analysis of the BRIGHT-4 trial
Source: PLoS Med. 2025 Jul 23;22(7):e1004679. doi: 10.1371/journal.pmed.1004679 (PMC12313069; doi:10.1371/journal.pmed.1004679)
Supplement: S2 Table — (DOCX) [file pmed.1004679.s002.docx]

S2 Table. Baseline characteristics of patients randomized to bivalirudin vs. heparin

|  | **Direct admission (N=3817)** | | | **Inter-hospital transfer (N=2121)** | | | |
| --- | --- | --- | --- | --- | --- | --- | --- |
|  | **Bivalirudin  (N=1890)** | **Heparin  (N=1927)** | ***P* Value** | | **Bivalirudin  (N=1084)** | **Heparin  (N=1037)** | ***P* Value** |
| Age, years | 60.6 ± 11.8 | 60.7 ± 11.9 | 0.63 | | 60.4 ± 12.7 | 60.3 ± 12.6 | 0.93 |
| Male | 1475 (78.0%) | 1523 (79.0%) | 0.46 | | 856 (79.0%) | 822 (79.3%) | 0.86 |
| Body mass index, kg/m^2^ | 24.86 ± 3.51 | 24.98 ± 3.58 | 0.31 | | 24.58 ± 3.61 | 24.92 ± 4.12 | 0.04 |
| Medical history |  |  |  | |  |  |  |
| Hypertension | 1003 (53.1%) | 990 (51.4%) | 0.30 | | 539 (49.7%) | 499 (48.1%) | 0.46 |
| Diabetes mellitus | 417 (22.1%) | 445 (23.1%) | 0.45 | | 236 (21.8%) | 239 (23.0%) | 0.48 |
| Smoking |  |  | 0.60 | |  |  | 0.43 |
| Active | 828 (43.8%) | 868 (45.0%) |  | | 528 (48.7%) | 510 (49.2%) |  |
| Former | 138 (7.3%) | 148 (7.7%) |  | | 86 (7.9%) | 97 (9.4%) |  |
| Never | 924 (48.9%) | 911 (47.3%) |  | | 470 (43.4%) | 430 (41.5%) |  |
| Previous MI | 146 (7.7%) | 156 (8.1%) | 0.67 | | 41 (3.8%) | 40 (3.9%) | 0.93 |
| Previous PCI | 150 (7.9%) | 152 (7.9%) | 0.96 | | 35 (3.2%) | 33 (3.2%) | 0.95 |
| Previous stroke | 228 (12.1%) | 224 (11.6%) | 0.67 | | 123 (11.3%) | 120 (11.6%) | 0.87 |
| Killip class |  |  | 0.50 | |  |  | 0.64 |
| I | 1138 (60.2%) | 1201 (62.3%) |  | | 661 (61.0%) | 638 (61.5%) |  |
| II | 560 (29.6%) | 528 (27.4%) |  | | 311 (28.7%) | 278 (26.8%) |  |
| III | 137 (7.2%) | 141 (7.3%) |  | | 79 (7.3%) | 83 (8.0%) |  |
| IV | 55 (2.9%) | 57 (3.0%) |  | | 33 (3.0%) | 38 (3.7%) |  |
| Hemoglobin, g/dL | 140.87 ± 18.20 | 141.00 ± 18.16 | 0.83 | | 138.99 ± 17.69 | 139.01 ± 18.33 | 0.98 |
| Anemia* | 350 (18.5%) | 373 (19.4%) | 0.51 | | 239 (22.0%) | 246 (23.7%) | 0.36 |
| Platelet count, 10^9^/L | 231.8 ± 71.8 | 228.3 ± 65.4 | 0.12 | | 224.7 ± 71.3 | 226.6 ± 67.6 | 0.54 |
| eGFR, ml/min/1.73m^2^ | 103.8 ± 33.6 | 103.7 ± 32.0 | 0.95 | | 107.7 ± 34.4 | 108.7 ± 35.4 | 0.51 |
| <60 ml/min/1.73m^2^ | 124 (6.6%) | 127 (6.6%) | 0.97 | | 80 (7.4%) | 72 (6.9%) | 0.70 |
| Symptom onset-to-FMC, hrs | 2.33 (1.05-4.81) | 2.39 (1.08-5.10) | 0.34 | | 1.67 (0.97-4.33) | 1.83 (0.95-4.00) | 0.73 |
| Transfer time, first hospital arrival to tertiary hospital arrival, min | - | - | - | | 135 (71-259) | 131 (73-252) | 0.75 |
| Symptom onset-to-tertiary hospital arrival, hrs | 2.50 (1.50-4.88) | 2.50 (1.50-5.25) | 0.35 | | 5.00 (3.00-9.00) | 4.88 (2.95-8.87) | 0.64 |
| ≤12h | 1507/1625 (92.7%) | 1516/1673 (90.6%) | 0.03 | | 772/937 (82.4%) | 734/890 (82.5%) | 0.96 |
| >12h | 118/1625 (7.3%) | 157/1673 (9.4%) |  | | 165/937 (17.6%) | 156/890 (17.5%) |  |
| Symptom onset-to-wire time, hrs | 3.92 (2.77-6.53) | 3.97 (2.83-6.92) | 0.11 | | 6.08 (4.03-10.03) | 5.93 (3.92-10.33) | 0.74 |
| FMC-to-wire time, hrs | 1.27 (0.98-1.78) | 1.28 (0.98-1.90) | 0.26 | | 3.63 (2.40-5.73) | 3.52 (2.50-5.38) | 0.68 |
| Tertiary hospital door-to-wire time, hrs | 1.20 (0.92-1.68) | 1.23 (0.92-1.78) | 0.14 | | 0.98 (0.77-1.40) | 0.97 (0.75-1.42) | 0.31 |
| Procedure duration, min^†^ | 28 (20-41) | 30 (20-42) | 0.11 | | 30 (20-43) | 29 (20-41) | 0.67 |

Data are shown as n (%), mean ± SD, or median (IQR). eGFR, estimated glomerular filtration rate; FMC, first medical contact (defined as arrival time at the first hospital); MI, myocardial infarction; PCI, percutaneous coronary intervention. *Defined as a hemoglobin concentration of <13 g/dL in men and <12 g/dL in women. ^†^Defined as the time from guiding catheter insertion to its withdrawal.
