# Supplementary material for: Analysis of inter-hospital transfer on clinical outcomes after primary percutaneous coronary intervention for ST-segment elevation myocardial infarction: A secondary analysis of the BRIGHT-4 trial
Source: PLoS Med. 2025 Jul 23;22(7):e1004679. doi: 10.1371/journal.pmed.1004679 (PMC12313069; doi:10.1371/journal.pmed.1004679)
Supplement: S3 Table — (DOCX) [file pmed.1004679.s003.docx]

S3 Table. Medications and procedural data in patients randomized to bivalirudin vs. heparin

|  | **Direct admission (N=3817)** | | | **Inter-hospital transfer (N=2121)** | | |
| --- | --- | --- | --- | --- | --- | --- |
|  | **Bivalirudin  (N=1890)** | **Heparin  (N=1927)** | ***P* Value** | **Bivalirudin  (N=1084)** | **Heparin  (N=1037)** | ***P* Value** |
| **Medications** |  |  |  |  |  |  |
| Study medications |  |  |  |  |  |  |
| Heparin | 19 (1.0%) | 1903 (98.8%) | - | 3 (0.3%) | 1028 (99.3%) | - |
| Total dose during PCI, U | - | 5600 (4900-6900) | - | - | 5460 (4550-6550) | - |
| Bivalirudin | 1870 (99.0%) | 24 (1.2%) | - | 1081 (99.7%) | 7 (0.7%) | - |
| Post-PCI infusion, any | 1869/1869 (100.0%) | - | - | 1053/1053 (100.0%) | - | - |
| Post-PCI infusion, hrs | 3.0 (2.3-4.0) | - | - | 3.0 (2.0-4.0) | - | - |
| Additional bolus of study drug | 75 (4.0%) | 678 (35.2%) | <0.0001 | 31 (2.9%) | 355 (34.3%) | <0.0001 |
| Tirofiban for procedural thrombotic complications | 227 (12.0%) | 250 (13.0%) | 0.37 | 118 (10.9%) | 161(15.6%) | 0.002 |
| Peak activated clotting time, sec | 319 (279-365) | 270 (240-325) | <0.0001 | 325 ­(275-366) | 261 (238-302) | <0.0001 |
| Dual antiplatelet therapy |  |  |  |  |  |  |
| Aspirin | 1874 (99.2%) | 1914 (99.3%) | 0.54 | 1080 (99.6%) | 1033 (99.6%) | 0.95 |
| P2Y12 inhibitor |  |  | 0.19 |  |  | 0.52 |
| Clopidogrel | 485 (25.7%) | 531 (27.6%) |  | 497 (45.8%) | 461 (44.5%) |  |
| Ticagrelor | 1405 (74.3%) | 1396 (72.4%) |  | 587 (54.2%) | 576 (55.5%) |  |
| **Procedural data** |  |  |  |  |  |  |
| Arterial access* |  |  | 0.24 |  |  | 0.41 |
| Transradial | 1755 (92.9%) | 1771 (91.9%) |  | 1025 (94.6%) | 970 (93.7%) |  |
| Transfemoral | 134 (7.1%) | 156 (8.1%) |  | 59 (5.4%) | 65 (6.3%) |  |
| Revascularization, any | 1869 (98.9%) | 1905 (98.9%) | 0.93 | 1053 (97.1%) | 1015 (97.9%) | 0.28 |
| Coronary arteries treated^†^ |  |  |  |  |  |  |
| Left main | 18 (1.0%) | 21 (1.1%) | 0.67 | 8 (0.8%) | 11 (1.1%) | 0.44 |
| Left anterior descending | 904 (48.4%) | 903 (47.4%) | 0.55 | 533 (50.6%) | 532 (52.4%) | 0.41 |
| Left circumflex | 235 (12.6%) | 250 (13.1%) | 0.61 | 138 (13.1%) | 112 (11.0%) | 0.15 |
| Right | 784 (42.0%) | 819 (43.0%) | 0.52 | 407 (38.7%) | 410 (40.4%) | 0.42 |
| Multivessel intervention | 75 (4.0%) | 88 (4.6%) | 0.36 | 34 (3.2%) | 54 (5.3%) | 0.02 |
| PCI | 1861 (98.5%) | 1900 (98.6%) | 0.73 | 1050 (96.9%) | 1009 (97.3%) | 0.55 |
| Drug-eluting stent implantation | 1680 (88.9%) | 1715 (89.0%) | 0.91 | 957 (88.3%) | 920 (88.7%) | 0.75 |
| Number of stents | 1.29 ± 0.54 | 1.30 ± 0.56 | 0.81 | 1.27 ± 0.53 | 1.30 ± 0.58 | 0.22 |
| Total length of stents, mm | 32.96 ± 15.86 | 32.93 ± 16.33 | 0.96 | 33.09 ± 16.30 | 33.79 ± 17.27 | 0.37 |
| Balloon angioplasty only | 181 (9.6%) | 185 (9.6%) | 0.98 | 93 (8.6%) | 89 (8.6%) | 0.99 |
| Thrombus aspiration | 373 (20.0%) | 373 (19.6%) | 0.75 | 160 (15.2%) | 153 (15.2%) | 0.96 |
| TIMI flow, site-assessed |  |  |  |  |  |  |
| Pre-PCI |  |  | 0.49 |  |  | 0.83 |
| 0 | 1406 (76.0%) | 1474 (77.9%) |  | 789 (75.3%) | 740 (74.2%) |  |
| 1 | 130 (7.0%) | 131 (6.9%) |  | 60 (5.7%) | 67 (6.7%) |  |
| 2 | 136 (7.3%) | 121 (6.4%) |  | 84 (8.0%) | 80 (8.0%) |  |
| 3 | 179 (9.7%) | 167 (8.8%) |  | 115 (11.0%) | 110 (11.0%) |  |
| Post-PCI |  |  | 0.21 |  |  | 0.25 |
| 0 | 6 (0.3%) | 15 (0.8%) |  | 5 (0.5%) | 8 (0.8%) |  |
| 1 | 2 (0.1%) | 2 (0.1%) |  | 2 (0.2%) | 3 (0.3%) |  |
| 2 | 16 (0.9%) | 22 (1.2%) |  | 12 (1.1%) | 21 (2.1%) |  |
| 3 | 1829 (98.7%) | 1855 (97.9%) |  | 1028 (98.2%) | 968 (96.8%) |  |
| Staged PCI within 30 days | 146 (7.7%) | 143 (7.4%) | 0.72 | 82 (7.6%) | 92 (8.9%) | 0.27 |
| Coronary artery bypass graft surgery | 8 (0.4%) | 5 (0.3%) | 0.39 | 3 (0.3%) | 6 (0.6%) | 0.29 |
| Coronary angiography only | 20 (1.1%) | 22 (1.1%) | 0.80 | 31 (2.9%) | 20 (1.9%) | 0.16 |
| None | 1 (0.1%) | 0 (0.0%) | 0.50 | 0 (0.0%) | 2 (0.2%) | 0.15 |

Data are shown as n (%), mean ± SD, or median (IQR). *Angiography was not performed in 1 patient in the direct admission group and in 2 patients in the inter-hospital transfer group. ^†^Per patient; some patients had more than one epicardial coronary artery treated during the index percutaneous coronary intervention or bypass graft procedure, so the total is more than 100%. PCI, percutaneous coronary intervention. TIMI, thrombolysis in myocardial infarction.
