# Supplementary material for: Analysis of inter-hospital transfer on clinical outcomes after primary percutaneous coronary intervention for ST-segment elevation myocardial infarction: A secondary analysis of the BRIGHT-4 trial
Source: PLoS Med. 2025 Jul 23;22(7):e1004679. doi: 10.1371/journal.pmed.1004679 (PMC12313069; doi:10.1371/journal.pmed.1004679)
Supplement: S4 Table — (DOCX) [file pmed.1004679.s004.docx]

S4 Table. Comparison of HRs (95%CI) for the primary outcome: Cox model vs. Cox frailty model

|  | **Unadjusted HR (95%CI)** | ***P* value** | **Adjusted HR (95%CI)** | ***P* value** |
| --- | --- | --- | --- | --- |
| **Inter-hospital transfer vs. Direct admission** |  |  |  |  |
| Cox models | 1.21 (0.92, 1.59) | 0.17 | 0.99 (0.73, 1.33) | 0.94 |
| Cox frailty models | 1.20 (0.91, 1.59) | 0.21 | 0.92 (0.67, 1.25) | 0.59 |
| **Inter-hospital transfer (Bivalirudin vs. Heparin)** |  |  |  |  |
| Cox models | 0.72 (0.47, 1.10) | 0.13 | 0.66 (0.42, 1.05) | 0.08 |
| Cox frailty models | 0.72 (0.47, 1.10) | 0.13 | 0.65 (0.41, 1.04) | 0.07 |
| **Direct admission**  **(Bivalirudin vs. Heparin)** |  |  |  |  |
| Cox models | 0.67 (0.47, 0.95) | 0.02 | 0.62 (0.43, 0.89) | 0.01 |
| Cox frailty models | 0.68 (0.48, 0.97) | 0.03 | 0.63 (0.44, 0.92) | 0.02 |
