# Supplementary material for: Analysis of inter-hospital transfer on clinical outcomes after primary percutaneous coronary intervention for ST-segment elevation myocardial infarction: A secondary analysis of the BRIGHT-4 trial
Source: PLoS Med. 2025 Jul 23;22(7):e1004679. doi: 10.1371/journal.pmed.1004679 (PMC12313069; doi:10.1371/journal.pmed.1004679)
Supplement: S5 Table — (DOCX) [file pmed.1004679.s005.docx]

S5 Table. Clinical outcomes at 30 days after propensity score matching

|  | **Inter-hospital transfer (N=1882)** | **Direct admission  (N=1882)** | **HR (95%CI)** | ***P* Value** |
| --- | --- | --- | --- | --- |
| Primary outcome: All-cause death or BARC types 3-5 bleeding | 67 (3.6%) | 65 (3.5%) | 1.03 (0.73, 1.45) | 0.87 |
| Death from any cause | 63 (3.3%) | 62 (3.3%) | 1.01 (0.71, 1.44) | 0.94 |
| From cardiovascular causes | 60 (3.2%) | 59 (3.1%) | 1.01 (0.71, 1.45) | 0.94 |
| BARC types 3-5 bleeding | 9 (0.5%) | 7 (0.4%) | 1.29 (0.48, 3.45) | 0.62 |
| Reinfarction | 16 (0.9%) | 8 (0.4%) | 2.00 (0.86, 4.68) | 0.11 |
| Ischemia-driven TVR | 8 (0.4%) | 8 (0.4%) | 1.00 (0.38, 2.66) | >0.999 |
| Stroke | 10 (0.5%) | 10 (0.5%) | 1.00 (0.42, 2.40) | >0.999 |
| Stent thrombosis | 16 (0.9%) | 11 (0.6%) | 1.46 (0.68, 3.14) | 0.34 |
| Acute (<24 hours) | 7 (0.4%) | 4 (0.2%) | 1.75 (0.51, 5.98) | 0.37 |
| Subacute (1-30 days) | 9 (0.5%) | 7 (0.4%) | 1.29 (0.48, 3.46) | 0.62 |
| MACCE* | 86 (4.6%) | 83 (4.4%) | 1.03 (0.77, 1.40) | 0.83 |
| BARC types 2-5 bleeding | 32 (1.7%) | 44 (2.3%) | 0.72 (0.46, 1.14) | 0.16 |
| All-cause death or BARC types 2-5 bleeding | 89 (4.7%) | 99 (5.3%) | 0.89 (0.67, 1.19) | 0.44 |
| Acquired thrombocytopenia^†^ | 85 (4.5%) | 63 (3.3%) | 1.36 (0.98, 1.88) | 0.07 |
| NACE^‡^ | 88 (4.7%) | 85 (4.5%) | 1.03 (0.77, 1.39) | 0.83 |

Event rates are number of events (Kaplan-Meier estimated percentages). MACCE, Major adverse cardiac or cerebral events. NACE, Net adverse clinical events. *MACCE includes all-cause death, myocardial infarction, ischemia-driven target vessel revascularization, or stroke. ^†^Defined as nadir platelet count of <150×10^9^ cells/L after the index procedure in patients in whom the baseline platelet count was ≥150×10^9^ cells/L. ^‡^NACE includes MACCE or BARC types 3-5 bleeding.
