# Supplementary material for: Analysis of inter-hospital transfer on clinical outcomes after primary percutaneous coronary intervention for ST-segment elevation myocardial infarction: A secondary analysis of the BRIGHT-4 trial
Source: PLoS Med. 2025 Jul 23;22(7):e1004679. doi: 10.1371/journal.pmed.1004679 (PMC12313069; doi:10.1371/journal.pmed.1004679)
Supplement: S6 Table — (DOCX) [file pmed.1004679.s006.docx]

S6 Table. Clinical outcomes at 30 days in patients with symptom onset-to-wire time ≤ 24 hours

|  | **Inter-hospital transfer (N=1468)** | **Direct admission  (N=2838)** | **Unadjusted HR (95%CI)** | ***P* Value** | **Adjusted HR (95%CI)** | ***P* Value** |
| --- | --- | --- | --- | --- | --- | --- |
| Primary outcome: All-cause death or BARC types 3-5 bleeding | 53 (3.6%) | 82 (2.9%) | 1.25 (0.89, 1.77) | 0.20 | 0.99 (0.68, 1.46) | 0.97 |
| Death from any cause | 46 (3.1%) | 78 (2.7%) | 1.14 (0.79, 1.64) | 0.48 | 0.91 (0.60, 1.36) | 0.63 |
| From cardiovascular causes | 46 (3.1%) | 75 (2.6%) | 1.19 (0.82, 1.71) | 0.36 | 0.95 (0.63, 1.43) | 0.80 |
| BARC types 3-5 bleeding | 9 (0.6%) | 9 (0.3%) | 1.93 (0.77, 4.87) | 0.16 | 1.51 (0.56, 4.02) | 0.41 |
| Reinfarction | 10 (0.7%) | 22 (0.8%) | 0.88 (0.42, 1.86) | 0.73 | 0.95 (0.44, 2.08) | 0.91 |
| Ischemia-driven TVR | 5 (0.3%) | 15 (0.5%) | 0.64 (0.23, 1.77) | 0.39 | 0.63 (0.22, 1.79) | 0.39 |
| Stroke | 9 (0.6%) | 18 (0.6%) | 0.97 (0.43, 2.15) | 0.93 | 1.11 (0.49, 2.53) | 0.80 |
| Stent thrombosis | 11 (0.7%) | 23 (0.8%) | 0.93 (0.45, 1.90) | 0.83 | 0.96 (0.46, 2.03) | 0.92 |
| Acute (<24 hours) | 7 (0.5%) | 8 (0.3%) | 1.69 (0.61, 4.66) | 0.31 | 1.61 (0.56, 4.67) | 0.38 |
| Subacute (1-30 days) | 4 (0.3%) | 15 (0.5%) | 0.52 (0.17, 1.55) | 0.24 | 0.57 (0.18, 1.77) | 0.33 |
| MACCE* | 61 (4.2%) | 119 (4.2%) | 0.99 (0.73, 1.35) | 0.95 | 0.88 (0.63, 1.23) | 0.47 |
| BARC types 2-5 bleeding | 30 (2.0%) | 79 (2.8%) | 0.73 (0.48, 1.11) | 0.14 | 0.74 (0.48, 1.14) | 0.17 |
| All-cause death or BARC types 2-5 bleeding | 74 (5.0%) | 149 (5.3%) | 0.96 (0.72, 1.27) | 0.76 | 0.87 (0.64, 1.17) | 0.35 |
| Acquired thrombocytopenia^†^ | 71 (4.9%) | 89 (3.1%) | 1.56 (1.14, 2.13) | 0.005 | 1.39 (1.00, 1.93) | 0.05 |
| NACE^‡^ | 66 (4.5%) | 122 (4.3%) | 1.05 (0.78, 1.41) | 0.77 | 0.92 (0.67, 1.27) | 0.61 |

Event rates are number of events (Kaplan-Meier estimated percentages). MACCE, Major adverse cardiac or cerebral events. NACE, Net adverse clinical events. *MACCE includes all-cause death, myocardial infarction, ischemia-driven target vessel revascularization, or stroke. ^†^Defined as nadir platelet count of <150×10^9^ cells/L after the index procedure in patients in whom the baseline platelet count was ≥150×10^9^ cells/L. ^‡^NACE includes MACCE or BARC types 3-5 bleeding.
