# Supplementary material for: Analysis of inter-hospital transfer on clinical outcomes after primary percutaneous coronary intervention for ST-segment elevation myocardial infarction: A secondary analysis of the BRIGHT-4 trial
Source: PLoS Med. 2025 Jul 23;22(7):e1004679. doi: 10.1371/journal.pmed.1004679 (PMC12313069; doi:10.1371/journal.pmed.1004679)
Supplement: S7 Table — (DOCX) [file pmed.1004679.s007.docx]

S7 Table. Clinical outcomes at 30 days according to randomization to bivalirudin vs. heparin in patients with symptom onset-to-wire time ≤ 24 hours

|  | **Direct admission (N=2838)** | | | | **Inter-hospital transfer (N=1468)** | | | |
| --- | --- | --- | --- | --- | --- | --- | --- | --- |
|  | **Bivalirudin  (N=1405)** | **Heparin (N=1433)** | **Adjusted HR (95%CI)** | ***P* value** | **Bivalirudin  (N=739)** | **Heparin  (N=729)** | **Adjusted HR (95%CI)** | ***P* value** |
| Primary outcome: All-cause death or BARC types 3-5 bleeding | 27 (1.9%) | 55 (3.8%) | 0.47 (0.29, 0.76) | 0.002 | 25 (3.4%) | 28 (3.8%) | 0.87 (0.48, 1.57) | 0.65 |
| Death from any cause | 26 (1.9%) | 52 (3.6%) | 0.48 (0.30, 0.79) | 0.004 | 24 (3.2%) | 22 (3.0%) | 1.15 (0.60, 2.19) | 0.68 |
| From cardiovascular causes | 26 (1.9%) | 49 (3.4%) | 0.52 (0.31, 0.85) | 0.009 | 24 (3.2%) | 22 (3.0%) | 1.15 (0.60, 2.19) | 0.68 |
| BARC types 3-5 bleeding | 2 (0.1%) | 7 (0.5%) | 0.21 (0.03, 1.29) | 0.09 | 1 (0.1%) | 8 (1.1%) | 0.03 (0.00, 0.48) | 0.01 |
| Reinfarction | 9 (0.6%) | 13 (0.9%) | 0.70 (0.30, 1.65) | 0.42 | 4 (0.5%) | 6 (0.8%) | 0.83 (0.20, 3.54) | 0.80 |
| Ischemia-driven TVR | 5 (0.4%) | 10 (0.7%) | 0.49 (0.17, 1.45) | 0.20 | 1 (0.1%) | 4 (0.5%) | 0.42 (0.01, 24.23) | 0.68 |
| Stroke | 9 (0.6%) | 9 (0.6%) | 1.09 (0.43, 2.77) | 0.86 | 5 (0.7%) | 4 (0.5%) | 1.29 (0.31, 5.30) | 0.73 |
| Stent thrombosis | 5 (0.4%) | 18 (1.3%) | 0.27 (0.10, 0.74) | 0.01 | 4 (0.5%) | 7 (1.0%) | 0.51 (0.13, 2.02) | 0.34 |
| Acute (<24 hours) | 2 (0.1%) | 6 (0.4%) | 0.31 (0.06, 1.57) | 0.16 | 2 (0.3%) | 5 (0.7%) | 0.23 (0.03, 1.92) | 0.18 |
| Subacute (1-30 days) | 3 (0.2%) | 12 (0.8%) | 0.25 (0.07, 0.90) | 0.03 | 2 (0.3%) | 2 (0.3%) | 0.88 (0.10, 7.58) | 0.91 |
| MACCE* | 47 (3.3%) | 72 (5.0%) | 0.66 (0.45, 0.96) | 0.03 | 30 (4.1%) | 31 (4.3%) | 1.08 (0.63, 1.86) | 0.77 |
| BARC types 2-5 bleeding | 41 (2.9%) | 38 (2.7%) | 1.13 (0.72, 1.77) | 0.59 | 11 (1.5%) | 19 (2.6%) | 0.51 (0.23, 1.12) | 0.09 |
| All-cause death or BARC types 2-5 bleeding | 64 (4.6%) | 85 (5.9%) | 0.77 (0.55, 1.07) | 0.13 | 35 (4.7%) | 39 (5.3%) | 0.88 (0.54, 1.44) | 0.62 |
| Acquired thrombocytopenia^†^ | 43 (3.1%) | 46 (3.2%) | 0.92 (0.60, 1.41) | 0.70 | 30 (4.1%) | 41 (5.6%) | 0.74 (0.46, 1.21) | 0.23 |
| NACE^‡^ | 48 (3.4%) | 74 (5.2%) | 0.65 (0.45, 0.95) | 0.02 | 30 (4.1%) | 36 (4.9%) | 0.90 (0.53, 1.51) | 0.68 |

Event rates are number of events (Kaplan-Meier estimated percentages). MACCE, Major adverse cardiac or cerebral events. NACE, Net adverse clinical events. *MACCE includes all-cause death, myocardial infarction, ischemia-driven target vessel revascularization, or stroke. ^†^Defined as nadir platelet count of <150×10^9^ cells/L after the index procedure in patients in whom the baseline platelet count was ≥150×10^9^ cells/L. ^‡^NACE includes MACCE or BARC types 3-5 bleeding.
