# Supplementary material for: Analysis of inter-hospital transfer on clinical outcomes after primary percutaneous coronary intervention for ST-segment elevation myocardial infarction: A secondary analysis of the BRIGHT-4 trial
Source: PLoS Med. 2025 Jul 23;22(7):e1004679. doi: 10.1371/journal.pmed.1004679 (PMC12313069; doi:10.1371/journal.pmed.1004679)
Supplement: S8 Table — (DOCX) [file pmed.1004679.s008.docx]

S8 Table. Clinical outcomes at 30 days according to symptom onset-to-wire time

|  | **≥3 hours**  **(N=3307)** | **<3 hours**  **(N=1174)** | ***P* Value** |
| --- | --- | --- | --- |
| **Primary outcome**: All-cause death or BARC types 3-5 bleeding | 117 (3.5%) | 22 (1.9%) | 0.005 |
| Death from any cause | 109 (3.3%) | 19 (1.6%) | 0.003 |
| From cardiovascular causes | 107 (3.2%) | 18 (1.5%) | 0.002 |
| BARC types 3-5 bleeding | 13 (0.4%) | 5 (0.4%) | 0.88 |
| Reinfarction | 20 (0.6%) | 13 (1.1%) | 0.08 |
| Ischemia-driven TVR | 10 (0.3%) | 10 (0.9%) | 0.02 |
| Stroke | 25 (0.8%) | 3 (0.3%) | 0.06 |
| Stent thrombosis | 21 (0.6%) | 13 (1.1%) | 0.11 |
| Acute (<24 hours) | 8 (0.2%) | 7 (0.6%) | 0.07 |
| Subacute (1-30 days) | 13 (0.4%) | 6 (0.5%) | 0.59 |
| MACCE* | 147 (4.4%) | 39 (3.3%) | 0.10 |
| BARC bleeding, types 2-5 | 81 (2.4%) | 32 (2.7%) | 0.60 |
| All-cause death or BARC types 2-5 bleeding | 182 (5.5%) | 49 (4.2%) | 0.08 |
| Acquired thrombocytopenia^†^ | 137 (4.1%) | 30 (2.6%) | 0.01 |
| NACE^‡^ | 152(4.6%) | 42(3.6%) | 0.14 |

Event rates are number of events (Kaplan-Meier estimated percentages). MACCE, Major adverse cardiac or cerebral events. NACE, Net adverse clinical events. *MACCE includes all-cause death, myocardial infarction, ischemia-driven target vessel revascularization, or stroke. ^†^Defined as nadir platelet count of <150×10^9^ cells/L after the index procedure in patients in whom the baseline platelet count was ≥150×10^9^ cells/L. ^‡^NACE includes MACCE or BARC types 3-5 bleeding.
