# Supplementary material for: Analysis of inter-hospital transfer on clinical outcomes after primary percutaneous coronary intervention for ST-segment elevation myocardial infarction: A secondary analysis of the BRIGHT-4 trial
Source: PLoS Med. 2025 Jul 23;22(7):e1004679. doi: 10.1371/journal.pmed.1004679 (PMC12313069; doi:10.1371/journal.pmed.1004679)
Supplement: S9 Table — (DOCX) [file pmed.1004679.s009.docx]

S9 Table. Clinical outcomes at 30 days in the direct admission versus inter-hospital transfer groups according to symptom onset-to-wire time

|  | **Direct admission (N=2914) *** | | | **Inter-hospital transfer (N=1567) *** | | | ***P* Value for interaction** |
| --- | --- | --- | --- | --- | --- | --- | --- |
|  | ≥ **3 hours (N=1922)** | **<3 hours (N=992)** | **Adjusted HR (95%CI)** | ≥ **3 hours (N=1385)** | **<3 hours (N=182)** | **Adjusted HR (95%CI)** |  |
| **Primary outcome**: All-cause death or BARC types 3-5 bleeding | 68 (3.5%) | 18 (1.8%) | 1.74 (1.02, 2.97) | 49 (3.5%) | 4 (2.2%) | 1.34 (0.44, 4.09) | 0.29 |
| Death from any cause | 65 (3.4%) | 17 (1.7%) | 1.80 (1.04, 3.13) | 44 (3.2%) | 2 (1.1%) | 2.09 (0.45, 9.62) | 0.88 |
| From cardiovascular  causes | 63 (3.3%) | 16 (1.6%) | 1.84 (1.05, 3.24) | 44 (3.2%) | 2 (1.1%) | 2.09 (0.45, 9.62) | 0.86 |
| BARC types 3-5 bleeding | 6 (0.3%) | 3 (0.3%) | 0.95 (0.21, 4.25) | 7 (0.5%) | 2 (1.1%) | 0.33 (0.04, 2.52) | 0.39 |
| Reinfarction | 12 (0.6%) | 10 (1.0%) | 0.57 (0.24, 1.35) | 8 (0.6%) | 3 (1.6%) | 0.28 (0.06, 1.35) | 0.37 |
| Ischemia-driven TVR | 5 (0.3%) | 10 (1.0%) | 0.26 (0.09, 0.79) | 5 (0.4%) | 0 (0.0%) | - | 0.99 |
| Stroke | 16 (0.8%) | 2 (0.2%) | 4.58 (1.04, 20.27) | 9 (0.6%) | 1 (0.5%) | 1.17 (0.13, 10.41) | 0.23 |
| Stent thrombosis | 12 (0.6%) | 11 (1.1%) | 0.50 (0.21, 1.15) | 9 (0.6%) | 2 (1.1%) | 0.22 (0.04, 1.33) | 0.82 |
| Acute (<24 hours) | 2 (0.1%) | 6 (0.6%) | 0.18 (0.03, 0.98) | 6 (0.4%) | 1 (0.5%) | 0.14 (0.01, 3.34) | 0.40 |
| Subacute (1-30 days) | 10 (0.5%) | 5 (0.5%) | 0.75 (0.25, 2.27) | 3 (0.2%) | 1 (0.5%) | 0.26 (0.01, 4.82) | 0.36 |
| MACCE^†^ | 88 (4.6%) | 35 (3.5%) | 1.22 (0.82, 1.82) | 59 (4.3%) | 4 (2.2%) | 1.80 (0.61, 5.32) | 0.75 |
| BARC bleeding, types 2-5 | 54 (2.8%) | 28 (2.8%) | 0.98 (0.61, 1.58) | 27 (1.9%) | 4 (2.2%) | 1.08 (0.31, 3.73) | 0.95 |
| All-cause death or BARC types 2-5 bleeding | 113 (5.9%) | 43 (4.3%) | 1.29 (0.89, 1.85) | 69 (5.0%) | 6 (3.3%) | 1.52 (0.59, 3.91) | 0.91 |
| Acquired thrombocytopenia^‡^ | 65 (3.4%) | 27 (2.7%) | 0.99 (0.62, 1.59) | 72 (5.2%) | 3 (1.6%) | 2.73 (0.85, 8.81) | 0.14 |
| NACE^§^ | 90 (4.7%) | 36 (3.6%) | 1.20 (0.81, 1.78) | 62 (4.5%) | 6 (3.3%) | 1.26 (0.51, 3.11) | 0.75 |

Event rates are number of events (Kaplan-Meier estimated percentages). *903 patients in the direct admission group and 554 patients in the inter-hospital transfer group with missing data on symptom onset-to-wire time were excluded. MACCE, Major adverse cardiac or cerebral events. NACE, Net adverse clinical events. ^†^MACCE includes all-cause death, myocardial infarction, ischemia-driven target vessel revascularization, or stroke. ^‡^Defined as nadir platelet count of <150×10^9^ cells/L after the index procedure in patients in whom the baseline platelet count was ≥150×10^9^ cells/L. ^§^NACE includes MACCE or BARC types 3-5 bleeding.
