# Supplementary material for: Analysis of inter-hospital transfer on clinical outcomes after primary percutaneous coronary intervention for ST-segment elevation myocardial infarction: A secondary analysis of the BRIGHT-4 trial
Source: PLoS Med. 2025 Jul 23;22(7):e1004679. doi: 10.1371/journal.pmed.1004679 (PMC12313069; doi:10.1371/journal.pmed.1004679)
Supplement: S10 Table — (DOCX) [file pmed.1004679.s010.docx]

S10 Table. Multivariable-adjusted HRs (95% CI) of 30-day primary outcome according to symptom onset-to-wire time

|  | **Primary outcome (%)** | **Adjusted HR (95%CI)** | ***P* value** |
| --- | --- | --- | --- |
| **All population** |  |  |  |
| < 3 hours (N=1174) | 22 (1.9%) | Reference | - |
| 3-6 hours (N=1679) | 42 (2.5%) | 1.31 (0.77, 2.23) | 0.32 |
| 6-12 hours (N=1015) | 52 (5.1%) | 2.15 (1.27, 3.64) | 0.004 |
| ≥ 12 hours (N=613) | 23 (3.8%) | 1.37 (0.74, 2.52) | 0.31 |
| **Direct admission** |  |  |  |
| < 3 hours (N=992) | 18 (1.8%) | Reference | - |
| 3-6 hours (N=1079) | 25 (2.3%) | 1.29 (0.69, 2.41) | 0.43 |
| 6-12 hours (N=539) | 30 (5.6%) | 2.55 (1.38, 4.69) | 0.003 |
| ≥ 12 hours (N=304) | 13 (4.3%) | 1.63 (0.78, 3.43) | 0.20 |
| **Inter-hospital transfer** |  |  |  |
| < 3 hours (N=182) | 4 (2.2%) | Reference | - |
| 3-6 hours (N=600) | 17 (2.8%) | 1.19 (0.37, 3.87) | 0.77 |
| 6-12 hours (N=476) | 22 (4.6%) | 1.80 (0.54, 6.00) | 0.34 |
| ≥ 12 hours (N=309) | 10 (3.2%) | 1.19 (0.33, 4.31) | 0.79 |

Event rates are number of events. Primary outcome, all-cause death or BARC types 3-5 bleeding.
