# Supplementary material for: Analysis of inter-hospital transfer on clinical outcomes after primary percutaneous coronary intervention for ST-segment elevation myocardial infarction: A secondary analysis of the BRIGHT-4 trial
Source: PLoS Med. 2025 Jul 23;22(7):e1004679. doi: 10.1371/journal.pmed.1004679 (PMC12313069; doi:10.1371/journal.pmed.1004679)
Supplement: S1 Text — (DOCX) [file pmed.1004679.s012.docx]

S1 Text. Institutional Review Boards

|  | **Institutional Review Boards (IRBs)** |
| --- | --- |
| 01 | General Hospital of Northern Theater Command, Shenyang, China |
| 02 | Kaifeng Central Hospital, Kaifeng, China |
| 03 | West China Hospital Sichuan University, Chengdu, China |
| 04 | Yuzhou City People’s Hospital, Yuzhou, China |
| 05 | Taian City Central Hospital, Taian, China |
| 06 | Tianjin Chest Hospital, Tianjin, China |
| 07 | Tangdu Hospital, Air Force Medical University, Xi’an, China |
| 08 | The Fifth People’s Hospital of Jinan, Jinan, China |
| 09 | The Second Hospital of Shenyang Medical College, Shenyang, China |
| 10 | Affillated Hospital of Qilu Medical University (The People’s Hospital of Xintai City), Xintai, China |
| 11 | Ganzhou People’s Hospital, Ganzhou, China |
| 12 | Bozhou People’s Hospital, Bozhou, China |
| 13 | Beijing Luhe Hospital of Capital Medical University, Beijing, China |
| 14 | Ganzhou Municipal Hospital, Ganzhou, China |
| 15 | Shangqiu First People’s Hospital, Shangqiu, China |
| 16 | Jiaozuo People’s Hospital, Jiaozuo, China |
| 17 | The First Affiliated Hospital of Anhui University of Science and Technology, Huannan, China |
| 18 | The Second Affiliated Hospital of Nanchang University, Nanchang, China |
| 19 | First Affiliated Hospital of Gannan Medical University, Ganzhou, China |
| 20 | The Third Affiliated Hospital of Xinxiang Medical University, Xinxiang, China |
| 21 | Yanbian University Hospital, Yanbian, China |
| 22 | The Fourth Affiliated Hospital of China Medical University, Shenyang, China |
| 23 | Heze Municipal Hospital, Heze, China |
| 24 | Huainan Cardiovascular Research Laboratory, Huannan, China |
| 25 | Xichang People’s Hospital, Xichang, China |
| 26 | The First Affiliated Hospital of Zhengzhou University, Zhengzhou, China |
| 27 | Peking University Third Hospital Yanqing Hospital, Beijing, China |
| 28 | The First Hospital of Lanzhou University, Lanzhou, China |
| 29 | Taihe County People’s Hospital, Taihe, China |
| 30 | Taizhou Central Hospital, Taizhou, China |
| 31 | The Affiliated Traditional Chinese Medicine Hospital of Southwest Medical University, Luzhou, China |
| 32 | Datong Third People’s Hospital, Datong, China |
| 33 | Luohe Central Hospital, Luohe, China |
| 34 | The Second Hospital of Jilin University, Changchun, China |
| 35 | China-Japan Union Hospital of Jilin University, Changchun, China |
| 36 | Fuyang People’s Hospital, Fuyang, China |
| 37 | The Affiliated Hospital of Guizhou Medical University, Guiyang, China |
| 38 | Meizhou Peopie’s Hospital, Meizhou, China |
| 39 | Qingdao Municipal Hospital, Qingdao, China |
| 40 | Affiliated Hospital of Zunyi Medical University, Zunyi, China |
| 41 | Bengbu Second People’s Hospital, Bengbu, China |
| 42 | Yueyang Central Hospital, Yueyang, China |
| 43 | The First Affiliated Hospital of Zhengzhou University, Zhengzhou, China |
| 44 | People’s Hospital of Pingchang County, Pingchang, China |
| 45 | Yunnan St.John’s Hospital, Kunming, China |
| 46 | Fuwai Central China Cardiovascular Hospital, Zhengzhou, China |
| 47 | The First Hospital of Liangshan, Liangshan, China |
| 48 | Central Hospital Affiliated to Shandong First Medical University, Jinan, China |
| 49 | Nanjing First Hospital, Nanjing, China |
| 50 | The First Affiliated Hospital of Nanchang University, Nanchang, China |
| 51 | Tianjin Medical University General Hospital, Tianjin, China |
| 52 | Hengshui Cardiovascular Hospital, Hengshui, China |
| 53 | Hospital Of Chengdu University of Traditional Chinese Medicine, Chengdu, China |
| 54 | Tongji Hospital, Tongji Medical College of HUST, Wuhan, China |
| 55 | Zaozhuang Central Hospital of Shandong Guoxin Healthcare Group, Zaozhuang, China |
| 56 | Shandong Second Provincial General Hospital, Jinan, China |
| 57 | Union Hospital, Tongji Medical College Of HUST, Wuhan, China |
| 58 | Xuzhou Central Hospital, Xuzhou, China |
| 59 | The First People’s Hospital of Ruzhou, Ruzhou, China |
| 60 | The Affiliated Hospital of Northwest University, Xi’an, China |
| 61 | Shanxi Cardiovascular Hospital, Xi’an, China |
| 62 | Suzhou Municipal Hospital of Anhui Province, Suzhou, China |
| 63 | Chongqing University Fuling Hospital, Chongqing, China |
| 64 | Heze Mudan People’s Hospital, Heze, China |
| 65 | The Fifth Affiliated Hospital of Xinjiang Medical University, Urumqi, China |
| 66 | Dalian No.3 People’s Hospital, Dalian, China |
| 67 | Linquan County People’s Hospital, Linquan, China |
| 68 | General Hospital of Fuxin Mining Industry Group of Liaoning Health Industry Group, Fuxin, China |
| 69 | General Hospital of Benxi Iron & Steel Industry Group of Liaoning Health Industry Group, Benxi, China |
| 70 | Yulin First People’s Hospital, Yulin, China |
| 71 | The First People’s Hospital of Xinmi, Xinmi, China |
| 72 | General Hospital of Fushun Mining Industry Group of Liaoning Health Industry Group, Fushun, China |
| 73 | The First Affiliated Hospital of Zhejiang Chinese Medical University, Hangzhou, China |
| 74 | The Second Affiliated Hospital of Xi’an Jiaotong University, Xi’an, China |
| 75 | No.2 People’s Hospital of Fuyang City, Fuyang, China |
| 76 | Xiangtan Central Hospital, Xiangtan, China |
| 77 | Loudi Central Hospital, Loudi, China |
| 78 | Jiangxi Provincial People’s Hospital, Nanchang, China |
| 79 | Wuhan Asia General Hospital, Wuhan, China |
| 80 | Jiangsu Taizhou People’s Hospital, Taizhou, China |
| 81 | Chifeng College Affiliated Hospital, Chifeng, China |
| 82 | Chengdu Second People’s Hospital, Chengdu, China |
| 83 | General Hospital of Tiefa Coal Industry Group of Liaoning Health Industry Group, Tiefa, China |
| 84 | Fushun Central Hospital, Fushun, China |
| 85 | Yan’an University Affiliated Hospital, Yan’an, China |
| 86 | Fourth Affiliated Hospital of Harbin Medical University, Harbin, China |
| 87 | Suixi Hospital, Suixi, China |
