# Supplementary material for: Analysis of inter-hospital transfer on clinical outcomes after primary percutaneous coronary intervention for ST-segment elevation myocardial infarction: A secondary analysis of the BRIGHT-4 trial
Source: PLoS Med. 2025 Jul 23;22(7):e1004679. doi: 10.1371/journal.pmed.1004679 (PMC12313069; doi:10.1371/journal.pmed.1004679)
Supplement: S2 Text — (DOCX) [file pmed.1004679.s013.docx]

S2 Text. Study protocol

|  | |
| --- | --- |
|  | |
| **BRIGHT-4**  **BivaliRudin with prolonged high-dose Infusion durinG primary PCI versus Heparin Trial** | |
| **title page** | |
|  | |
| **Sponsor:**  **Version** | General Hospital of Northern Theatre Command  1.10 |
| **Contract Research Organization:** | Liaoning Excellent Technology Co., Ltd. |

**PROTOCOL SYNOPSIS**

| **Title of Study** | BRIGHT-4 Study - BivaliRudin with prolonged high-dose Infusion durinG primary PCI: a Multicenter, Randomized, Open-label Trial |
| --- | --- |
| **Sponsor** | General Hospital of Northern Theatre Command |
| **Principal** **Investigator** | Yaling Han, MD, Professor of Medicine  Director, Dept. Cardiology, General Hospital of Northern Theatre Command |
| **Co-principal Investigator** | Gregg W. Stone, MD, FACC  Professor of Icahn School of Medicine at Mount Sinai, Director of Academic Affairs for the Mount Sinai Heart Health System |
| **Major Study Center** | General Hospital of Northern Theatre Command |
| **Study Centers** | 85 sites in China |
| **Study Design** | Prospective, multicenter, randomized, open-label, active drug parallel-controlled study |
| **Purposes** | The primary study objective is to evaluate the net clinical benefit of bivalirudin with prolonged high-dose infusion vs heparin alone among ST elevation myocardial infarction (STEMI) patients undergoing primary PCI. |
| **Study Population** | STEMI patients undergoing primary PCI |
| **Study Medication** | Bivalirudin (Jiangsu Hansoh Pharmaceutical Group Co., Ltd.) |
| **Control Medication** | Heparin Sodium Injection (Shanghai Pharma No.1 Biochemical Pharmaceutical Co., Ltd.) |
| **Primary** **Endpoint** | The incidence of a composite of all-cause death or Bleeding Academic Research Consortium (BARC) types 3-5 bleeding at 30 days after randomization. |
| **Secondary Endpoints** | 1. Net adverse clinical events (NACE, defined as a composite of all-cause death, recurrent myocardial infarction, ischemia-driven target vessel revascularization, stroke or BARC types 3-5 bleeding); 2. The incidence of a composite of all-cause death or BARC types 2-5 bleeding; 3. Stent thrombosis (defined as the definite or probable stent thrombosis according to Academic Research Consortium definition); 4. Major adverse cardiac and cerebral events (MACCE, defined as a composite of all-cause death, recurrent myocardial infarction, ischemia-driven target vessel revascularization or stroke); 5. BARC types 3-5 bleeding; 6. BARC types 2-5 bleeding; 7. Thrombocytopenia (defined as post-PCI platelet counts <150×10^9^/L for patients with baseline platelet count >150×10^9^/L); 8. The incidence of each individual event, including all-cause death, cardiac death, non-cardiac death, recurrent myocardial infraction, ischemia-driven target vessel revascularization, ischemia-driven target lesion revascularization and stroke.   All secondary endpoints are assessed at 30 days, 6 and 12 months after randomization. |
| **Inclusion Criteria** | (All must be present)   1. Any age; 2. STEMI patients with symptom onset ≤48h with primary PCI planned (STEMI is defined as ST elevation ≥1mm in ≥2 contiguous leads or new LBBB, or persistent ischemic symptoms in the presence of RBBB^7^); 3. Patients requiring staged revascularization of non-culprit vessels within 30 days may also be enrolled. In such cases the antithrombotic agents and procedures in the staged PCI must be consistent with the index PCI, especially the peri-procedural antithrombotic agents including assigned heparin vs. bivalirudin, and tirofiban); 4. No contraindications to dual antiplatelet therapy, and dual antiplatelet agents must be administrated according to current guidelines before PCI (loading doses and maintenance doses of aspirin and clopidogrel/ticagrelor); 5. The subject or legal representatives has been fully informed and written informed consent provided. |
| **Exclusion Criteria** | (All must be absent)   1. Not suitable for primary PCI as judged by the physician; 2. STEMI treated by thrombolysis; 3. Patients received heparin, LMWH, fondaparinux, bivalirudin, or GP IIb/IIIa inhibitor (GPI) within 48 hours before the index PCI; 4. Mechanical complications (such as ventricular septal rupture, papillary muscle rupture or acute mitral regurgitation, etc.); 5. Known allergy or contraindications to heparin, bivalirudin, aspirin, clopidogrel or ticagrelor; 6. Patients are participating in other drug or device studies; 7. Patients for whom the investigators consider participation in the study to be inappropriate or who may be nonadherent to the study protocol during the follow-up period, such as those with psychiatric disorder, alcoholism or drug addiction. |
| **Follow-up Period** | Primary end point: 30 days; Secondary end points:12 months. |
| **Treatment Plan** | 1. Bivalirudin group: in the cath lab, bivalirudin 0.75 mg/kg intravenous bolus loading dose is started before angiography, and is immediately followed by an intravenous infusion of 1.75 mg/kg/h until 2-4 hours after primary PCI. Activated clotting time (ACT) is monitored 5 min after the first administration, and if ACT is <225 s (Hemotec method), an additional intravenous injection of 0.30 mg/kg of bivalirudin is administered, to ensure the re-checked ACT is >225 s.   Bivalirudin bolus loading dose is not affected by renal function. Bivalirudin maintenance infusion dose should be reduced to 1.0mg/kg/h for patients with eGFR <30 ml/min and 0.25 mg/kg/h for patients on dialysis.  eGFR = (140-age) × weight(kg)/72×CCr(mg/dl) × (0.85 if female).   1. Heparin group: in the cath lab, an intravenous bolus injection of heparin 70 U/kg is given before angiography. ACT is monitored 5 min after the first administration, and if the ACT is <225 s (Hemotec method), an additional intravenous injection of 1000 U of heparin is administered, to ensure the re-checked ACT is >225 s.   Routine use of GPI during procedure is strictly forbidden. Intravenous or intra-coronary tirofiban is only allowed for target vessel slow blood flow, no-reflow, obvious thrombus or thrombosis complication during PCI. If necessary, intravenous tirofiban should be started with a 10-25 μg/kg bolus infusion (given over more than 5 min) followed by 0.15 μg/kg/min maintenance infusion for up to 18 hours. For patients with eGFR <60ml/min, the maintenance dose is reduced to 0.075 μg/kg/min. Intra-coronary injection of tirofiban should be 500-750μg per injection, with repeated injection intervals of 3-5 min and total dose no more than 1500-2250μg.  LWMH after primary PCI can be administered at operator’s discretion.  Loading dose of aspirin (300mg), and P2Y12 receptor inhibitor clopidogrel (300/600mg) or ticagrelor (180mg) shall be administered before index PCI. Other medications are prescribed according to current guidelines. Trans-radial (preferred) or -femoral approach, and stent type are at operator’s discretion. |
| **Statistical Methods** | |
| **Endpoint Analyses** | The efficacy analysis will be performed on the full analysis set according to the intention-to-treat principle. This analysis will be repeated in the per protocol set to support the primary results.  The safety analysis will be performed on the safety set, defined as the population who received at least 1 dose of study drugs and who have data observed at any time after randomization until the end of the study. |
| **Sample Size Determination** | The main aim of this study is to investigate if bivalirudin with prolonged high-dose infusion after PCI is superior to heparin alone in reducing 30-day death or major bleeding for patients with STEMI undergoing primary PCI. The null hypothesis (H_0_) for this analysis is that the incidence of primary endpoint in the intervention group is same as that of the control group, namely P_0_=P_1_. The alternative hypothesis (H_1_) is that the incidences of primary endpoints in the two groups are not equal, namely P_0_≠P_1_. Assuming the incidence of the primary endpoint in heparin group is 3.3%, and assuming 1% loss of the primary endpoint at 30 days, 3000 evaluable patients in each group (6000 in total) are planned to be enrolled and randomly assigned in a 1:1 ratio, in order to provide 80% power to detect a 1.2% absolute risk reduction (35% relative risk reduction) in the bivalirudin group in comparison with the heparin group with a 2-sided type I error of 0.05. |

**TABLE OF CONTENTS**

**[PROTOCOL SYNOPSIS](file:///C:\\Users\\momo\\Desktop\\transferred-in%20and%20direct%20admission\\direct%20admission%20vs.%20inter-hospital%20transfer\\投稿6\\upload-revision\\PLOS修稿\\BRIGHT-4%20Supplementary_Appendix%20revised%20clean.docx#_Toc101778660)**

**[PROTOCOL SIGNATURE PAGE](file:///C:\\Users\\momo\\Desktop\\transferred-in%20and%20direct%20admission\\direct%20admission%20vs.%20inter-hospital%20transfer\\投稿6\\upload-revision\\PLOS修稿\\BRIGHT-4%20Supplementary_Appendix%20revised%20clean.docx#_Toc101778661)**

**[1. INTRODUCTION](file:///C:\\Users\\momo\\Desktop\\transferred-in%20and%20direct%20admission\\direct%20admission%20vs.%20inter-hospital%20transfer\\投稿6\\upload-revision\\PLOS修稿\\BRIGHT-4%20Supplementary_Appendix%20revised%20clean.docx#_Toc101778662)**

**[2. STUDY OBJECTIVES](file:///C:\\Users\\momo\\Desktop\\transferred-in%20and%20direct%20admission\\direct%20admission%20vs.%20inter-hospital%20transfer\\投稿6\\upload-revision\\PLOS修稿\\BRIGHT-4%20Supplementary_Appendix%20revised%20clean.docx#_Toc101778663)**

**[3. BASIC INFORMATION OF MEDICATIONS](file:///C:\\Users\\momo\\Desktop\\transferred-in%20and%20direct%20admission\\direct%20admission%20vs.%20inter-hospital%20transfer\\投稿6\\upload-revision\\PLOS修稿\\BRIGHT-4%20Supplementary_Appendix%20revised%20clean.docx#_Toc101778664)**

[3.1 Bivalirudin](file:///C:\\Users\\momo\\Desktop\\transferred-in%20and%20direct%20admission\\direct%20admission%20vs.%20inter-hospital%20transfer\\投稿6\\upload-revision\\PLOS修稿\\BRIGHT-4%20Supplementary_Appendix%20revised%20clean.docx#_Toc101778665)

[3.1.1 Pharmacology](file:///C:\\Users\\momo\\Desktop\\transferred-in%20and%20direct%20admission\\direct%20admission%20vs.%20inter-hospital%20transfer\\投稿6\\upload-revision\\PLOS修稿\\BRIGHT-4%20Supplementary_Appendix%20revised%20clean.docx#_Toc101778666)

[3.1.2 Drug interaction](file:///C:\\Users\\momo\\Desktop\\transferred-in%20and%20direct%20admission\\direct%20admission%20vs.%20inter-hospital%20transfer\\投稿6\\upload-revision\\PLOS修稿\\BRIGHT-4%20Supplementary_Appendix%20revised%20clean.docx#_Toc101778667)

[3.1.3 Adverse drug reaction](file:///C:\\Users\\momo\\Desktop\\transferred-in%20and%20direct%20admission\\direct%20admission%20vs.%20inter-hospital%20transfer\\投稿6\\upload-revision\\PLOS修稿\\BRIGHT-4%20Supplementary_Appendix%20revised%20clean.docx#_Toc101778668)

[3.2 Heparin](file:///C:\\Users\\momo\\Desktop\\transferred-in%20and%20direct%20admission\\direct%20admission%20vs.%20inter-hospital%20transfer\\投稿6\\upload-revision\\PLOS修稿\\BRIGHT-4%20Supplementary_Appendix%20revised%20clean.docx#_Toc101778669)

[3.2.1 Pharmacology](file:///C:\\Users\\momo\\Desktop\\transferred-in%20and%20direct%20admission\\direct%20admission%20vs.%20inter-hospital%20transfer\\投稿6\\upload-revision\\PLOS修稿\\BRIGHT-4%20Supplementary_Appendix%20revised%20clean.docx#_Toc101778670)

[3.2.2 Drug interaction](file:///C:\\Users\\momo\\Desktop\\transferred-in%20and%20direct%20admission\\direct%20admission%20vs.%20inter-hospital%20transfer\\投稿6\\upload-revision\\PLOS修稿\\BRIGHT-4%20Supplementary_Appendix%20revised%20clean.docx#_Toc101778671)

[3.2.3 Adverse drug reaction](file:///C:\\Users\\momo\\Desktop\\transferred-in%20and%20direct%20admission\\direct%20admission%20vs.%20inter-hospital%20transfer\\投稿6\\upload-revision\\PLOS修稿\\BRIGHT-4%20Supplementary_Appendix%20revised%20clean.docx#_Toc101778672)

**[4. STUDY DESIGN 12](file:///C:\\Users\\momo\\Desktop\\transferred-in%20and%20direct%20admission\\direct%20admission%20vs.%20inter-hospital%20transfer\\投稿6\\upload-revision\\PLOS修稿\\BRIGHT-4%20Supplementary_Appendix%20revised%20clean.docx#_Toc101778673)**

[4.1 Overall study design and flow chart](file:///C:\\Users\\momo\\Desktop\\transferred-in%20and%20direct%20admission\\direct%20admission%20vs.%20inter-hospital%20transfer\\投稿6\\upload-revision\\PLOS修稿\\BRIGHT-4%20Supplementary_Appendix%20revised%20clean.docx#_Toc101778674)

[4.2 Treatment](file:///C:\\Users\\momo\\Desktop\\transferred-in%20and%20direct%20admission\\direct%20admission%20vs.%20inter-hospital%20transfer\\投稿6\\upload-revision\\PLOS修稿\\BRIGHT-4%20Supplementary_Appendix%20revised%20clean.docx#_Toc101778675)

[4.3 Randomization](file:///C:\\Users\\momo\\Desktop\\transferred-in%20and%20direct%20admission\\direct%20admission%20vs.%20inter-hospital%20transfer\\投稿6\\upload-revision\\PLOS修稿\\BRIGHT-4%20Supplementary_Appendix%20revised%20clean.docx#_Toc101778676)

[4.4 Blinding](file:///C:\\Users\\momo\\Desktop\\transferred-in%20and%20direct%20admission\\direct%20admission%20vs.%20inter-hospital%20transfer\\投稿6\\upload-revision\\PLOS修稿\\BRIGHT-4%20Supplementary_Appendix%20revised%20clean.docx#_Toc101778677)

[4.5 Schedule of data collection](file:///C:\\Users\\momo\\Desktop\\transferred-in%20and%20direct%20admission\\direct%20admission%20vs.%20inter-hospital%20transfer\\投稿6\\upload-revision\\PLOS修稿\\BRIGHT-4%20Supplementary_Appendix%20revised%20clean.docx#_Toc101778678)

**[5. STUDY POPULATION](file:///C:\\Users\\momo\\Desktop\\transferred-in%20and%20direct%20admission\\direct%20admission%20vs.%20inter-hospital%20transfer\\投稿6\\upload-revision\\PLOS修稿\\BRIGHT-4%20Supplementary_Appendix%20revised%20clean.docx#_Toc101778679)**

[5.1 Inclusion criteria](file:///C:\\Users\\momo\\Desktop\\transferred-in%20and%20direct%20admission\\direct%20admission%20vs.%20inter-hospital%20transfer\\投稿6\\upload-revision\\PLOS修稿\\BRIGHT-4%20Supplementary_Appendix%20revised%20clean.docx#_Toc101778680)

[5.2 Exclusion Criteria](file:///C:\\Users\\momo\\Desktop\\transferred-in%20and%20direct%20admission\\direct%20admission%20vs.%20inter-hospital%20transfer\\投稿6\\upload-revision\\PLOS修稿\\BRIGHT-4%20Supplementary_Appendix%20revised%20clean.docx#_Toc101778681)

[5.3 Criteria of patient removal, treatment discontinuation and drop out](file:///C:\\Users\\momo\\Desktop\\transferred-in%20and%20direct%20admission\\direct%20admission%20vs.%20inter-hospital%20transfer\\投稿6\\upload-revision\\PLOS修稿\\BRIGHT-4%20Supplementary_Appendix%20revised%20clean.docx#_Toc101778682)

[5.3.1 Criteria of removal](file:///C:\\Users\\momo\\Desktop\\transferred-in%20and%20direct%20admission\\direct%20admission%20vs.%20inter-hospital%20transfer\\投稿6\\upload-revision\\PLOS修稿\\BRIGHT-4%20Supplementary_Appendix%20revised%20clean.docx#_Toc101778683)

[5.3.2 Criteria of treatment discontinuation](file:///C:\\Users\\momo\\Desktop\\transferred-in%20and%20direct%20admission\\direct%20admission%20vs.%20inter-hospital%20transfer\\投稿6\\upload-revision\\PLOS修稿\\BRIGHT-4%20Supplementary_Appendix%20revised%20clean.docx#_Toc101778684)

[5.3.3 Criteria for drop out](file:///C:\\Users\\momo\\Desktop\\transferred-in%20and%20direct%20admission\\direct%20admission%20vs.%20inter-hospital%20transfer\\投稿6\\upload-revision\\PLOS修稿\\BRIGHT-4%20Supplementary_Appendix%20revised%20clean.docx#_Toc101778685)

**[6. STUDY CONDUCTION](file:///C:\\Users\\momo\\Desktop\\transferred-in%20and%20direct%20admission\\direct%20admission%20vs.%20inter-hospital%20transfer\\投稿6\\upload-revision\\PLOS修稿\\BRIGHT-4%20Supplementary_Appendix%20revised%20clean.docx#_Toc101778686)**

[6.1 Study medications](file:///C:\\Users\\momo\\Desktop\\transferred-in%20and%20direct%20admission\\direct%20admission%20vs.%20inter-hospital%20transfer\\投稿6\\upload-revision\\PLOS修稿\\BRIGHT-4%20Supplementary_Appendix%20revised%20clean.docx#_Toc101778687)

[6.2 Follow-up](file:///C:\\Users\\momo\\Desktop\\transferred-in%20and%20direct%20admission\\direct%20admission%20vs.%20inter-hospital%20transfer\\投稿6\\upload-revision\\PLOS修稿\\BRIGHT-4%20Supplementary_Appendix%20revised%20clean.docx#_Toc101778688)

[6.3 Treatment plan](file:///C:\\Users\\momo\\Desktop\\transferred-in%20and%20direct%20admission\\direct%20admission%20vs.%20inter-hospital%20transfer\\投稿6\\upload-revision\\PLOS修稿\\BRIGHT-4%20Supplementary_Appendix%20revised%20clean.docx#_Toc101778689)

[6.3.1 Bivalirudin group (intervention group)](file:///C:\\Users\\momo\\Desktop\\transferred-in%20and%20direct%20admission\\direct%20admission%20vs.%20inter-hospital%20transfer\\投稿6\\upload-revision\\PLOS修稿\\BRIGHT-4%20Supplementary_Appendix%20revised%20clean.docx#_Toc101778690)

[6.3.2 Heparin group (heparin group)](file:///C:\\Users\\momo\\Desktop\\transferred-in%20and%20direct%20admission\\direct%20admission%20vs.%20inter-hospital%20transfer\\投稿6\\upload-revision\\PLOS修稿\\BRIGHT-4%20Supplementary_Appendix%20revised%20clean.docx#_Toc101778691)

[6.3.3 Concomitant medications](file:///C:\\Users\\momo\\Desktop\\transferred-in%20and%20direct%20admission\\direct%20admission%20vs.%20inter-hospital%20transfer\\投稿6\\upload-revision\\PLOS修稿\\BRIGHT-4%20Supplementary_Appendix%20revised%20clean.docx#_Toc101778692)

**[7. ENDPOINTS](file:///C:\\Users\\momo\\Desktop\\transferred-in%20and%20direct%20admission\\direct%20admission%20vs.%20inter-hospital%20transfer\\投稿6\\upload-revision\\PLOS修稿\\BRIGHT-4%20Supplementary_Appendix%20revised%20clean.docx#_Toc101778693)**

[7.1 Primary endpoint](file:///C:\\Users\\momo\\Desktop\\transferred-in%20and%20direct%20admission\\direct%20admission%20vs.%20inter-hospital%20transfer\\投稿6\\upload-revision\\PLOS修稿\\BRIGHT-4%20Supplementary_Appendix%20revised%20clean.docx#_Toc101778694)

[7.2 Secondary endpoints](file:///C:\\Users\\momo\\Desktop\\transferred-in%20and%20direct%20admission\\direct%20admission%20vs.%20inter-hospital%20transfer\\投稿6\\upload-revision\\PLOS修稿\\BRIGHT-4%20Supplementary_Appendix%20revised%20clean.docx#_Toc101778695)

**[8. SAFETY](file:///C:\\Users\\momo\\Desktop\\transferred-in%20and%20direct%20admission\\direct%20admission%20vs.%20inter-hospital%20transfer\\投稿6\\upload-revision\\PLOS修稿\\BRIGHT-4%20Supplementary_Appendix%20revised%20clean.docx#_Toc101778696)**

[8.1 AE](file:///C:\\Users\\momo\\Desktop\\transferred-in%20and%20direct%20admission\\direct%20admission%20vs.%20inter-hospital%20transfer\\投稿6\\upload-revision\\PLOS修稿\\BRIGHT-4%20Supplementary_Appendix%20revised%20clean.docx#_Toc101778697)

[8.2 SAE](file:///C:\\Users\\momo\\Desktop\\transferred-in%20and%20direct%20admission\\direct%20admission%20vs.%20inter-hospital%20transfer\\投稿6\\upload-revision\\PLOS修稿\\BRIGHT-4%20Supplementary_Appendix%20revised%20clean.docx#_Toc101778698)

[8.3 Reporting of SAE](file:///C:\\Users\\momo\\Desktop\\transferred-in%20and%20direct%20admission\\direct%20admission%20vs.%20inter-hospital%20transfer\\投稿6\\upload-revision\\PLOS修稿\\BRIGHT-4%20Supplementary_Appendix%20revised%20clean.docx#_Toc101778699)

[8.4 Management of AE](file:///C:\\Users\\momo\\Desktop\\transferred-in%20and%20direct%20admission\\direct%20admission%20vs.%20inter-hospital%20transfer\\投稿6\\upload-revision\\PLOS修稿\\BRIGHT-4%20Supplementary_Appendix%20revised%20clean.docx#_Toc101778700)

[8.4.1 Follow-up of AE](file:///C:\\Users\\momo\\Desktop\\transferred-in%20and%20direct%20admission\\direct%20admission%20vs.%20inter-hospital%20transfer\\投稿6\\upload-revision\\PLOS修稿\\BRIGHT-4%20Supplementary_Appendix%20revised%20clean.docx#_Toc101778701)

[8.4.2 Treatment of AE](file:///C:\\Users\\momo\\Desktop\\transferred-in%20and%20direct%20admission\\direct%20admission%20vs.%20inter-hospital%20transfer\\投稿6\\upload-revision\\PLOS修稿\\BRIGHT-4%20Supplementary_Appendix%20revised%20clean.docx#_Toc101778702)

[8.4.3 Measures for AE](file:///C:\\Users\\momo\\Desktop\\transferred-in%20and%20direct%20admission\\direct%20admission%20vs.%20inter-hospital%20transfer\\投稿6\\upload-revision\\PLOS修稿\\BRIGHT-4%20Supplementary_Appendix%20revised%20clean.docx#_Toc101778703)

**[9. DATA COLLECTION](file:///C:\\Users\\momo\\Desktop\\transferred-in%20and%20direct%20admission\\direct%20admission%20vs.%20inter-hospital%20transfer\\投稿6\\upload-revision\\PLOS修稿\\BRIGHT-4%20Supplementary_Appendix%20revised%20clean.docx#_Toc101778704)**

[9.1 Filling in CRF](file:///C:\\Users\\momo\\Desktop\\transferred-in%20and%20direct%20admission\\direct%20admission%20vs.%20inter-hospital%20transfer\\投稿6\\upload-revision\\PLOS修稿\\BRIGHT-4%20Supplementary_Appendix%20revised%20clean.docx#_Toc101778705)

[9.2 CRF auditing](file:///C:\\Users\\momo\\Desktop\\transferred-in%20and%20direct%20admission\\direct%20admission%20vs.%20inter-hospital%20transfer\\投稿6\\upload-revision\\PLOS修稿\\BRIGHT-4%20Supplementary_Appendix%20revised%20clean.docx#_Toc101778706)

**[10. STATISTICAL ANALYSIS](file:///C:\\Users\\momo\\Desktop\\transferred-in%20and%20direct%20admission\\direct%20admission%20vs.%20inter-hospital%20transfer\\投稿6\\upload-revision\\PLOS修稿\\BRIGHT-4%20Supplementary_Appendix%20revised%20clean.docx#_Toc101778707)**

[10.1 Sample size determination](file:///C:\\Users\\momo\\Desktop\\transferred-in%20and%20direct%20admission\\direct%20admission%20vs.%20inter-hospital%20transfer\\投稿6\\upload-revision\\PLOS修稿\\BRIGHT-4%20Supplementary_Appendix%20revised%20clean.docx#_Toc101778708)

[10.2 Description of analysis sets](file:///C:\\Users\\momo\\Desktop\\transferred-in%20and%20direct%20admission\\direct%20admission%20vs.%20inter-hospital%20transfer\\投稿6\\upload-revision\\PLOS修稿\\BRIGHT-4%20Supplementary_Appendix%20revised%20clean.docx#_Toc101778709)

[10.3 Methods of statistical analyses](file:///C:\\Users\\momo\\Desktop\\transferred-in%20and%20direct%20admission\\direct%20admission%20vs.%20inter-hospital%20transfer\\投稿6\\upload-revision\\PLOS修稿\\BRIGHT-4%20Supplementary_Appendix%20revised%20clean.docx#_Toc101778710)

[10.4 Subgroup analyses](file:///C:\\Users\\momo\\Desktop\\transferred-in%20and%20direct%20admission\\direct%20admission%20vs.%20inter-hospital%20transfer\\投稿6\\upload-revision\\PLOS修稿\\BRIGHT-4%20Supplementary_Appendix%20revised%20clean.docx#_Toc101778711)

**[11. QUALITY CONTROL](file:///C:\\Users\\momo\\Desktop\\transferred-in%20and%20direct%20admission\\direct%20admission%20vs.%20inter-hospital%20transfer\\投稿6\\upload-revision\\PLOS修稿\\BRIGHT-4%20Supplementary_Appendix%20revised%20clean.docx#_Toc101778712)**

**[12. CEC](file:///C:\\Users\\momo\\Desktop\\transferred-in%20and%20direct%20admission\\direct%20admission%20vs.%20inter-hospital%20transfer\\投稿6\\upload-revision\\PLOS修稿\\BRIGHT-4%20Supplementary_Appendix%20revised%20clean.docx#_Toc101778713)**

**[13. EC](file:///C:\\Users\\momo\\Desktop\\transferred-in%20and%20direct%20admission\\direct%20admission%20vs.%20inter-hospital%20transfer\\投稿6\\upload-revision\\PLOS修稿\\BRIGHT-4%20Supplementary_Appendix%20revised%20clean.docx#_Toc101778714)**

**[14. STUDY MANAGEMENT](file:///C:\\Users\\momo\\Desktop\\transferred-in%20and%20direct%20admission\\direct%20admission%20vs.%20inter-hospital%20transfer\\投稿6\\upload-revision\\PLOS修稿\\BRIGHT-4%20Supplementary_Appendix%20revised%20clean.docx#_Toc101778715)**

[14.1 Modification in the protocol](file:///C:\\Users\\momo\\Desktop\\transferred-in%20and%20direct%20admission\\direct%20admission%20vs.%20inter-hospital%20transfer\\投稿6\\upload-revision\\PLOS修稿\\BRIGHT-4%20Supplementary_Appendix%20revised%20clean.docx#_Toc101778716)

**[15. RESPOSIBILITIES OF EACH PARTY](file:///C:\\Users\\momo\\Desktop\\transferred-in%20and%20direct%20admission\\direct%20admission%20vs.%20inter-hospital%20transfer\\投稿6\\upload-revision\\PLOS修稿\\BRIGHT-4%20Supplementary_Appendix%20revised%20clean.docx#_Toc101778717)**

[15.1 Responsibilities of sponsor](file:///C:\\Users\\momo\\Desktop\\transferred-in%20and%20direct%20admission\\direct%20admission%20vs.%20inter-hospital%20transfer\\投稿6\\upload-revision\\PLOS修稿\\BRIGHT-4%20Supplementary_Appendix%20revised%20clean.docx#_Toc101778718)

[15.2 Responsibilities of investigators](file:///C:\\Users\\momo\\Desktop\\transferred-in%20and%20direct%20admission\\direct%20admission%20vs.%20inter-hospital%20transfer\\投稿6\\upload-revision\\PLOS修稿\\BRIGHT-4%20Supplementary_Appendix%20revised%20clean.docx#_Toc101778719)

[15.3 Responsibilities of CRO](file:///C:\\Users\\momo\\Desktop\\transferred-in%20and%20direct%20admission\\direct%20admission%20vs.%20inter-hospital%20transfer\\投稿6\\upload-revision\\PLOS修稿\\BRIGHT-4%20Supplementary_Appendix%20revised%20clean.docx#_Toc101778720)

**[16. REFERENCES](file:///C:\\Users\\momo\\Desktop\\transferred-in%20and%20direct%20admission\\direct%20admission%20vs.%20inter-hospital%20transfer\\投稿6\\upload-revision\\PLOS修稿\\BRIGHT-4%20Supplementary_Appendix%20revised%20clean.docx#_Toc101778721)**

**[17. APPENDIX 1 - DEFINITION](file:///C:\\Users\\momo\\Desktop\\transferred-in%20and%20direct%20admission\\direct%20admission%20vs.%20inter-hospital%20transfer\\投稿6\\upload-revision\\PLOS修稿\\BRIGHT-4%20Supplementary_Appendix%20revised%20clean.docx#_Toc101778722)**

**PROTOCOL SIGNATURE PAGE**

I have read this clinical investigation plan and appendices and agree to adhere to the requirements. I will provide copies of this clinical investigation plan and all pertinent information to the trial personnel under my supervision. I will discuss this material with them and ensure they are fully informed regarding the medication and the conduct of the trial.

I will conduct the trial in accordance with the clinical investigation plan, Good Clinical Practice guidelines, the Declaration of Helsinki, EN ISO 14155:2011 (Clinical Investigation of Medical Devices for Human Subjects - Good Clinical Practice), as well as local regulations. I also accept respective revisions to the clinical investigation plan approved by authorized personnel of the ARO and by regulatory authorities.

*Do not copy, distribute, or share this document with others without prior written authorization.*

Investigator name (print):

Investigator name (signature):

Date:

Institution Name (print):

**1. INTRODUCTION**

Bivalirudin, a direct thrombin inhibitor, is a synthetic hirudin derivative with 20 amino acids. Bivalirudin directly and specifically binds and inhibits thrombin, significantly prolongs the activated clotting time (ACT), and thus provides an anticoagulant effect for preventing thrombosis. Bivalirudin rapidly and reversely effects result in a low bleeding risk, and has a better safety profile in comparison with traditional heparin. Thrombin induces platelet activation, leading to platelet aggregation and granule secretion. Therefore, bivalirudin may also indirectly suppress platelet activation through inhibiting thrombin. In recent years abundant studies have demonstrated that bivalirudin decreases bleeding events for patients undergoing primary percutaneous coronary intervention (PCI) compared with heparin, with comparable antithrombotic efficacy, and may even reduce the risk of cardiac death [1,2]. However, some clinical trials showed that the risk of stent thrombosis (ST) was elevated with bivalirudin among patients undergoing primary PCI [3], and several meta-analyses also had consistent findings [4,5].

This potential detrimental risk of bivalirudin may be associated with its administration strategy. The half-life of bivalirudin is approximately 25 min. Patients with ST segment elevation myocardial infarction (STEMI) have a delayed bioavailability of P2Y12 receptor inhibitors including both traditional clopidogrel and the novel potent agents ticagrelor and prasugrel, not reaching their maximum platelet inhibition effects until 6-8 hours after administration. Therefore, a “window period” lack of antithrombotic effect may exist if the bivalirudin infusion is stopped immediately in the cath lab as in the early clinical trials in primary PCI, with patients in a hypercoagulable state. A meta-analysis verified the above hypothesis, showing that most bivalirudin-related acute ST events occurred during the first few hours after PCI [4]. To address this issue, our team conducted the BRIGHT study exploring the impact of peri-procedural bivalirudin administration on clinical outcomes in patients undergoing emergency PCI [6]. With 2194 recruited patients from 82 cites, our study pioneered the continuous high-dose (1.75mg/kg/h) bivalirudin maintenance infusion treatment after the procedure and reported that with this high-dose infusion bivalirudin substantially reduces bleeding events without increasing the risk of ST, providing an important benefit for patients undergoing emergency PCI [6].

Based on the BRIGHT study, the current study aims to further compare the safety and efficacy of bivalirudin with a prolonged high-dose infusion versus heparin alone among STEMI patients undergoing primary PCI, with the goal of providing critical clinical evidence to select the optimal peri-procedural anticoagulation strategy for these high-risk patients.

**2. STUDY OBJECTIVES**

The perspective, randomized, open-label, active drug parallel-controlled BRIGHT-4 study aims to explore the efficacy and safety of bivalirudin with a prolonged high-dose infusion for 2-4 hours after procedure versus heparin alone during primary PCI in STEMI patients at 30 days, 6 and 12 months.

**3. BASIC INFORMATION OF MEDICATIONS**

**3.1 Bivalirudin**

**3.1.1 Pharmacology**

Bivalirudin is a direct, specific, reversible thrombin inhibitor. Bivalirudin directly binds to either free or clot-bound thrombin at the catalytic site and anion-binding exosite and provides an anticoagulation effect independent of antithrombin (AT) Ⅲ and platelet granule secretion. Thrombin inhibition by bivalirudin is rapid and transient, because thrombin induces proteolytic cleavage of Arg3-Pro4 bond of bivalirudin restoring the acting site of thrombin. In vitro, bivalirudin prolongs the activated partial thromboplastin time, thrombin time and prothrombin time in both healthy volunteers and patients undergoing PCI in a concentration-dependent manner. Coagulation is immediately inhibited after bivalirudin intravenous infusion and recovers to normal level 1 hour after bivalirudin discontinuation. The half-life of bivalirudin is approximately 25 min. Currently no antidote drug exists for the overdose of bivalirudin.

**3.1.2 Drug interaction**

Bivalirudin does not interact with plasma proteins or erythrocytes. Concomitant use of heparin, warfarin or thrombolytics increases the bleeding risk of bivalirudin.

**3.1.3 Adverse drug reaction**

Bleeding is the common adverse drug reaction (ADR) of bivalirudin, mostly occurring at the access site of PCI, and may also occur at non-access sites. If blood pressure or volume suddenly decreases, or other unknown symptoms appear, bivalirudin infusion shall be immediately stopped. Other infrequent ADRs include thrombocytopenia, anemia, hypersensitivity, ventricular tachycardia, angina pectoris, bradyarrhythmia, dyspnea, rash, back pain, headache, hypotension, etc.

**3.2 Heparin**

**3.2.1 Pharmacology**

Heparin is a mucopolysaccharide sulfate, composed of glucosamine sulfate, α-L-iduronic acid 2-sulfate, N-acetylglucosamine, and D-glucuronic acid. The average molecular weight of heparin is 12000-15000 Da, with a range from 3000 to 50000 Da. The major anticoagulation mechanism of heparin is binding to AT Ⅲ, inactivating Factor Ⅱa, Ⅹa, Ⅸa and XIIa. The ratio of heparin-AT complex to Factor Xa and IIa inhibition is about 1:1.

After administration, 80% of heparin combines with serum albumin, and a small proportion is absorbed by blood cells or diffuses to the extravascular space. After biotransformation in the reticuloendothelial system of liver, heparin, including a small part in unchanged form, is excreted in urine. The excretion rate of heparin is dependent on infusion dose. The half-life of 100, 400 or 800 U/kg heparin is 1, 2.5 and 5 h, respectively. The excretion may be delayed in individuals with chronic kidney disfunction or obesity, leading to heparin accumulation. The time to onset of heparin is associated with the route of administration. Intravenous infusion immediately reaches the maximum effect but varies greatly among individuals. Subcutaneous administration of heparin prolongs the duration of action.

**3.2.2 Drug interaction**

The bleeding risk may be increased when heparin is administered in combination with drugs as follows: 1) dicumarol and its derivative, leading to reduction of Factor Ⅸ and bleeding; 2) non-steroidal anti-inflammatory drugs including aspirin, mefenamic acid, salicylic acid, inhibiting platelet function, and potentially inducing gastrointestinal bleeding; 3) Dipyridamole or dextran inhibiting platelet function; 4) Adrenocorticosteroid and adrenocorticotropic hormone inducing gastrointestinal ulceration and bleeding; 4) Others including uric acid, tissue plasminogen activator, urokinase and streptokinase, etc.

The following drugs are contraindicated with heparin: kanamycin, amikacin, daunorubicin, erythromycin lactate, gentamicin sulfate, hydrocortisone sodium succinate, polymyxin B, doxorubicin, tobramycin, vancomycin, cefoperazone, cefamandole, [cefalotin](javascript:;) [sodium](javascript:;), chloroquine, chlorpromazine, promazine, anesthetic analgesics.

**3.2.3 Adverse drug reaction**

Spontaneous bleeding is the major ADR of heparin, and it is recommended to measure blood clotting time before administration. If severe bleeding occurs after heparin administration, intravenous infusion of protamine sulfate may be used to neutralize heparin’s effect. Additionally, heparin may cause hypersensitivity and thrombocytopenia, often 5 to 9 days after administration. Therefore, the platelet count shall be monitored during the first month after treatment. Transient hair loss or diarrhea occasionally happens. Heparin may also cause osteoporosis and spontaneous bone fracture. Long-term use of heparin in individuals with hepatic dysfunction may induce depletion of AT Ⅲ, leading to bleeding.

**4. STUDY DESIGN**

**4.1 Overall study design and flow chart**

This is a prospective, multicenter, randomized, open-label, active drug parallel-controlled superiority study.


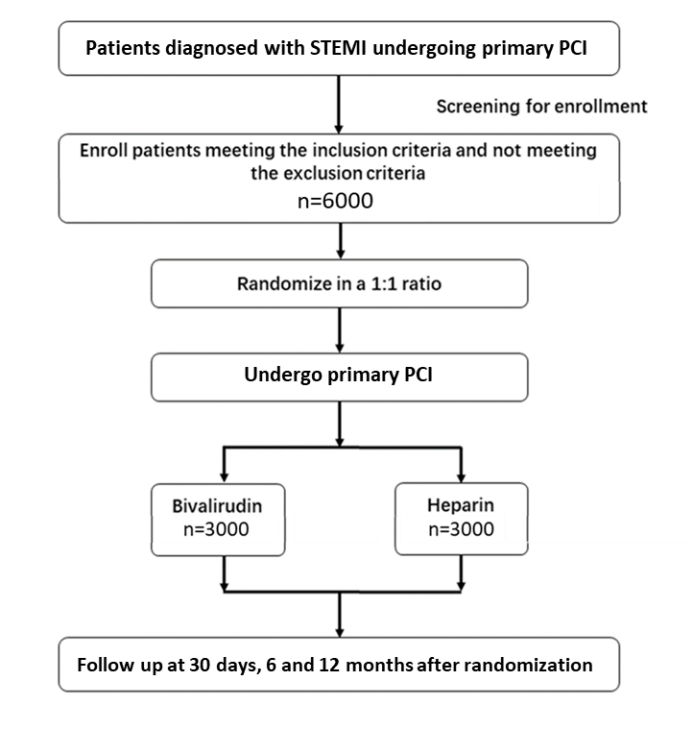


**Figure 1. BRIGHT-4 Trial Design**

**4.2 Treatment**

All enrolled STEMI patients will be randomized after providing written consent. Study medication treatment will be administered according to assigned group before angiography in the catheterization laboratory. ACT is monitored 5 min after the first administration, and if ACT is <225 s (Hemotec method), an additional intravenous injection of 0.30 mg/kg of bivalirudin, or 1000 U of heparin according to assigned group is administered to ensure the re-checked ACT is >225 s.

GPI use is strictly limited. Intravenous or intra-coronary tirofiban is only allowed for target vessel slow blood flow, no-reflow, obvious thrombus or thrombosis complication during PCI. At operator’s discretion in the above indications, tirofiban 10-25μg/kg intravenous infusion for loading dose is administered over 5 min, followed by intravenous infusion of 0.15μg/kg/min maintenance dose for up to 18 hours. For patients with eGFR <60ml/min, the maintenance dose is reduced to 0.075μg/kg/min. For intra-coronary infusion, tirofiban 500-750μg per injection with a 1500-2250 total dose is recommended, and the time interval is 3-5 min between injections.

LWMH after primary PCI is administered at operator’s discretion. Loading dose of aspirin 300mg, and P2Y12 receptor inhibitor clopidogrel 300/600mg or ticagrelor 180mg shall be administered before index PCI. Other medications are prescribed according to current guidelines. Trans-radial (preferred) or -femoral approach, and stent type are at operator’s discretion.

Telephone or outpatient follow-up will be conducted at 30 days, 6 and 12 months after PCI. If any adverse event occurs, subjects shall immediately contact their physicians.

**4.3 Randomization**

A centralized randomization method and electronic data collection system are used in this study. Patients meeting ALL the inclusion criteria and NONE of the exclusion criteria will be assigned to intervention or control group via centralized randomization system after signing informed consent. An interactive web response system will generate a randomization number for each eligible patient, and simultaneously track the enrollments from all participating sites.

**4.4 Blinding**

This study has a single-blind design. Investigators and subjects are aware, but the independent clinical events committee (CEC) is unaware of the type of study medication subjects receive.

**4.5 Schedule of data collection**

**Table 1. Schedule of data collection**

| **Study procedures** | **Visit 1** | **Visit 2** | **Visit 3** | **Visit 4** | **Visit 5** |
| --- | --- | --- | --- | --- | --- |
|  | **PCI** | **Discharge** | **30 days** | **6 months** | **12 months** |
| Window Period | 0d |  | ±7d | ±14d | ±30d |
| Eligibility Criteria | X |  |  |  |  |
| Patient Informed Consent | X |  |  |  |  |
| Randomization | X |  |  |  |  |
| Medical History/ Demographics | X |  |  |  |  |
| Vital Signs | X |  |  |  |  |
| Blood and Urine Routine Test^1^ | X |  |  |  |  |
| Blood Biochemical Examination^2^ | X |  |  |  |  |
| Cardiac Markers (including TnT or TNI/CK/CK-MB)^3^ | X |  |  |  |  |
| ACT Test | X |  |  |  |  |
| 12-Leads ECG^4^ | X |  |  |  |  |
| Key Symptom Inspection | X | X | X | X | X |
| Clinical Assessment | X | X | X | X | X |
| Concomitant Medications | X | X | X | X | X |
| Coronary Artery Angiography | X |  |  |  |  |
| Cardiovascular Events | X | X | X | X | X |
| Adverse Events | X | X | X | X | X |

1-4 are routine tests, repeated tests during in-hospital period are at physicians’ discretion.

**5. STUDY POPULATION**

Enrolled subjects should be patients with STEMI undergoing primary PCI.

**5.1 Inclusion criteria**

1. Any age;
2. STEMI patients with symptom onset ≤48 hours with primary PCI planned (STEMI is defined as ST elevation ≥1mm in ≥2 contiguous leads or new LBBB, or persistent ischemic symptoms in the presence of RBBB [7]);
3. Patients requiring staged revascularization of non-culprit vessels within 30 days may also be enrolled. In such cases the antithrombotic agents and procedures in the staged PCI must be consistent with the index PCI, especially the peri-procedural antithrombotic agents including assigned heparin vs. bivalirudin, and tirofiban);
4. No contraindications to dual antiplatelet therapy, and dual antiplatelet agents must be administrated according to current guidelines before PCI (loading doses and maintenance doses of aspirin and clopidogrel/ticagrelor);
5. The subject or legal representatives has been fully informed and written informed consent provided.

**5.2 Exclusion Criteria**

1. Not suitable for primary PCI as judged by the physician;
2. STEMI treated by thrombolysis;
3. Patients received heparin, LMWH, fondaparinux, bivalirudin, or GPI within 48 hours before the index PCI;
4. Mechanical complications (such as ventricular septal rupture, papillary muscle rupture or acute mitral regurgitation, etc.);
5. Known allergy or contraindications to heparin, bivalirudin, aspirin, clopidogrel or ticagrelor;
6. Patients are participating in other drug or device studies;
7. Patients whom the investigators consider participation in the study to be inappropriate or who may be nonadherent to the study protocol during the follow-up period, such as those with psychiatric disorder, alcoholism or drug addiction.

**5.3 Criteria of patient removal, treatment discontinuation and drop out**

**5.3.1 Criteria of removal**

Patient should be withdrawn from the study if one of the following occurs:

- Misdiagnosis;
- No record of physical or laboratory test results;
- Does not meet the inclusion criteria or meet at least one exclusion criteria.

Withdrawn patients will not be included in the efficacy analysis but may be included in the safety analysis if there are any records regarding efficacy or safety.

**5.3.2 Criteria of treatment discontinuation**

Discontinuation of any study medication is defined as stopping the experimental treatment. The reason for discontinuation should be clearly noted in the original medical records and case report form (CRF).

Treatment should be discontinued if:

- If patient withdraws informed consent;
- For intolerable side effects of study medications;
- For severe allergy that cannot be relieved by anti-allergic therapy;
- At investigator’s discretion, if adverse events occur or for abnormal laboratory test results indicating that continuation of treatment would be inappropriate;
- At investigator’s discretion, of the patient suffered severe complications which necessitates withdrawing study medications;
- At investigator’s discretion, if treatment discontinuation is in the patient’s best interest;
- At investigator’s discretion, for major deviation of the study protocol;
- At investigator’s discretion, for severe adverse events that threaten the patient’s health.

**5.3.3 Criteria for drop out**

All patients enrolled in the trial shall be retained until completion of follow-up. Patients have the right to withdraw from the study at any time without penalty and would not lose any benefits. The reasons that patients drop out may include but are not limited to:

- Severe adverse events causing termination of the study;
- Unexpected illness for which further treatment would be inappropriate;
- Lost to follow-up;
- Patient withdrew informed consent;
- Other reasons causing termination of the study;
- Poor patient compliance which might affect efficacy or safety.

If patients met the above criteria and stop participating in the study, investigators must:

- Inform the study supervisor;
- Complete the “completion of study” page in the case report form;
- If patient withdrawal is caused by an AE/SAE, continue the clinical follow-up until the AE/SAE is resolved or stabilized until the 28^th^ day from last study treatment. Follow-up results shall be recorded in the initial medical record and CRF;
- Perform necessary examinations related to visit termination for all subjects who discontinue treatment. Finish the follow-up procedures that can be performed if subjects request withdrawal of informed consent or lose the ability to freely provide informed consent (such as incarceration or treatment for mental or physical illness).

**6. STUDY CONDUCTION**

**6.1 Study medications**

**Table 2. Identity of Investigational Product**

| **Study drug** | **The form and dosage of drug** | **Manufacturer** |
| --- | --- | --- |
| Zelang (bivalirudin) | 0.25g/bottle | Jiangsu Hansoh Pharmaceutical Group Co., Ltd. |
| Heparin Sodium Injection (heparin) | 12500U/2ml | Shanghai Pharma No.1 Biochemical Pharmaceutical Co., Ltd. |

**6.2 Follow-up**

Patients enrolled in this study will be followed-up at 30 days, 6 and 12 months after randomization.

**6.3 Treatment plan**

- - 1. **Bivalirudin group (intervention group)**

In the cath lab, bivalirudin 0.75 mg/kg intravenous bolus loading dose is started before angiography, and is immediately followed by an intravenous infusion of 1.75 mg/kg/h until 2-4 hours after the index primary PCI. ACT is monitored 5 min after the first administration, and if ACT is <225 s (Hemotec method), an additional intravenous injection of 0.30 mg/kg of bivalirudin is administered, to ensure the re-checked ACT is >225 s.

Bivalirudin bolus loading dose is not affected by renal function. Bivalirudin maintenance infusion dose should be reduced to 1.0mg/kg/h for patients with eGFR <30 ml/min and to 0.25 mg/kg/h for patients on dialysis.

**6.3.2 Heparin group (heparin group)**

In the cath lab, an intravenous bolus injection of heparin 70 U/kg is given before angiography. ACT is monitored 5 min after the first administration, and if the ACT is <225 s (Hemotec method), an additional intravenous injection of 1000 U of heparin is administered, to ensure the re-checked ACT is >225 s.

**6.3.3 Concomitant medications**

1. Routine use of GPI during procedure is strictly forbidden. Intravenous or intra-coronary tirofiban is only allowed for target vessel slow blood flow, no-reflow, obvious thrombus or thrombosis complication during PCI. If necessary, intravenous tirofiban should be started with a 10-25μg/kg bolus infusion (given over more than 5 min) followed by 0.15μg/kg/min maintenance infusion for up to 18 hours. For patients with eGFR <60ml/min, the maintenance dose is reduced to 0.075μg/kg/min. Intra-coronary injection of tirofiban should be 500-750μg per injection, with repeated injection intervals of 3-5 min and total dose no more than 1500-2250μg. LWMH after primary PCI can be administered at operator’s discretion
2. Clopidogrel 300/600mg or ticagrelor 180mg for loading dose (LD), followed by clopidogrel 75mg per day or ticagrelor 90mg twice per day for 12-month maintenance dose (MD) is administered. Aspirin 300mg for LD, followed by 100mg per day for long-term MD is administered. Other medications are prescribed according to current guidelines. Trans-radial (preferred) or -femoral approach, and stent type are at operator’s discretion.

**7. ENDPOINTS**

**7.1 Primary endpoint**

The incidence of a composite of all-cause death or Bleeding Academic Research Consortium (BARC) types 3-5 bleeding at 30 days after randomization.

**7.2 Secondary endpoints**

1. Net adverse clinical events (NACE, defined as a composite of all-cause death, recurrent myocardial infarction, ischemia-driven target vessel revascularization, stroke or BARC types 3-5 bleeding);
2. The incidence of a composite of all-cause death or BARC types 2-5 bleeding;
3. Stent thrombosis (defined as the definite or probable stent thrombosis according to Academic Research Consortium definition);
4. Major adverse cardiac and cerebral events (MACCE, defined as a composite of all-cause death, recurrent myocardial infarction, ischemia-driven target vessel revascularization or stroke);
5. BARC types 3-5 bleeding;
6. BARC types 2-5 bleeding;
7. Thrombocytopenia (defined as post-PCI platelet counts <150×10^9^/L for patients with baseline platelet count >150×10^9^/L);
8. The incidence of each individual event, including all-cause death, cardiac death, non-cardiac death, recurrent myocardial infraction, ischemia-driven target vessel revascularization, ischemia-driven target lesion revascularization and stroke.

Secondary endpoints are assessed at 30 days, 6 and 12 months after randomization.

**8. SAFETY**

There are two classifications of adverse events: adverse event (AE), and serious adverse event (SAE). AEs must be monitored until resolved or adequately explained. All serious and unanticipated AEs must be reported, regardless of the cause.

**8.1 AE**

Any adverse medical events occurring in a clinical trial, not necessarily having a causal relationship with the study treatments. AEs could be any signs (including abnormal laboratory values), symptoms, or temporary disease, whether related or unrelated to the study drug use. An AE may be categorized as the following:

- Mild – signs or symptoms that do not interfere with the patients’ usual activities or is transient and resolves without treatment of sequelae;
- Moderate – interferes with the patients’ usual activities and/or requires symptomatic treatment;
- Severe – symptoms cause severe discomfort and significant impact for the patient’s usual activities and requires treatment.

**8.2 SAE**

A study-related event that is fatal, life-threatening, requires inpatient hospitalization or prolongation of an existing hospitalization, requires intervention to prevent permanent impairment/damage, or results meet in one or more of the following criteria:

- Death;
- Is life-threatening;
- Requires in-patient hospitalization or prolongation of existing hospitalization;
- Persistent or significant disability/incapacity or substantial disruption of the ability to conduct normal life functions;
- A congenital abnormality or birth defect;
- An important medical event that may jeopardize the subject or may require medical intervention to prevent one of the outcomes listed above.

**8.3 Reporting of SAE**

The investigator must complete the SAE form for each SAE, then sign and date the report. The event should be treated immediately and reported to the Investigational Review Board (IRB)/Ethics Committee (EC), national regulatory authority, sponsor and CRO within 24 hours (from the point in time when the SAE is identified by the site) via telephone or fax. The sponsor and investigator should immediately analyze the SAE, take necessary measures to protect the safety and interests of subjects, promptly report to the Primary Investigator and the drug supervision and administration department, and also inform other investigators who are in charge of clinical trials involving the same drug. The report form for the SAE should be submitted within 24 hours, including filling in the corresponding section in CRF, and sending an email containing the event information to the sponsor. If the SAE is determined to be definitely associated with study medications, the sponsor should provide the fees for treatment and corresponding economic compensation.

The investigator shall record a detailed death case report, if death happens. If post-mortem autopsy is conducted, the investigator shall send a copy of the post-mortem autopsy report to the sponsor as soon as possible.

Any SAE that occurred before the last administration of study medication should be reported according to the above procedures. SAE beyond this time limit shall be reported to the sponsor and relevant authorities if the investigator deems the SAE relevant to the study medication.

**8.4 Management of AE**

**8.4.1 Follow-up of AE**

For ALL AEs the subject’s course must be monitored until the event has subsided, including completing the study or finishing the final report of this subject.

Even though they have completed the study, subjects with SAEs should receive clinical follow-up until the adverse event has subsided, or investigators believe the patient has reached a chronic or stable condition.

**8.4.2 Treatment of AE**

The investigator should firstly assess the AE and provide appropriate medical treatments, then reasonably adjust treatments according to subject’s condition, and finally conduct the corresponding visits.

**8.4.3 Measures for AE**

Data of AEs should be collected during the whole period of study. The investigator should record ALL AEs on corresponding CRFs and provide supervision until recovery.

**9. DATA COLLECTION**

**9.1 Filling in CRF**

- All qualified subjects who have signed the informed consent shall have all items in the CRFs carefully recorded. No blank items or missing items shall be allowed, and those that do not need to be filled in shall be marked with a diagonal line;
- Data recorded in the CRF should be verified with source medical records to ensure accuracy;
- Any change in the CRF should be marked with a deletion line. The correction should be written with the investigator’s signature and date;
- The copy of original laboratory test sheets and electrocardiograms (ECG) should be pasted in CRFs;
- Any abnormal data (deviation from normal or clinically unacceptable) should be verified by the investigator;
- Details are in accordance with the instruction of filling in the CRF.

**9.2 CRF auditing**

- Monitors should audit patient consents and enrolment periodically at each participating center;
- To ensure that data in the CRF are correct and are consistent with the original medical records;
- To ensure all errors and missing data are corrected or illustrated and signed by the investigator;
- To ensure each therapeutic change, concomitant medication and complication is recorded accurately.
- To ensure that all patient withdrawals or loss of follow-up are recorded in the CRF;
- To ensure that all AEs are recorded and all SAEs are reported per standard procedure;
- To ensure that the delivery, storage and recycling of study drugs abide to regulatory requirements and are recorded.

**10. STATISTICAL ANALYSIS**

**10.1 Sample size determination**

The study hypothesis is that bivalirudin with a prolonged full-dose infusion is superior to heparin alone for the 30-day primary endpoint, a composite of all-cause death or BARC types 3 to 5 bleeding after primary PCI in patients with STEMI.

H_0_: P_I_=P_C_

H_1_: P_I_≠P_C_, α=0.05 (two-sided)

P_I_ denotes the primary endpoint rate in the bivalirudin group, P_C_ is the primary endpoint rate in the control group.

Power calculations are based on a superiority comparison for the primary endpoint, a composite of all-cause death or BARC types 3-5 bleeding at 30 days after primary PCI in patients with STEMI. Assuming the incidence of the primary endpoint in heparin group is 3.3%, and assuming 1% loss to follow-up for the primary endpoint data at 30 days, 3000 evaluable patients in each group (6000 in total) are planned to be enrolled and randomly assigned in a 1:1 ratio, in order to provide 80% power to detect a 1.2% absolute risk reduction (35% relative risk reduction) in the bivalirudin group in comparison with the heparin group with a 2-sided type I error of 0.05.

**10.2 Description of analysis sets**

**Full analysis set (FAS):** Patients who meet the study inclusion criteria, sign the written informed consent and receive study medications after randomization and have any assessment for the primary events will be included in FAS. FAS will be used as baseline characteristics and endpoint analysis.

**Per-protocol set (PPS):** Population will consist of all randomized subjects without any major deviations (including but not limited to violations of inclusion or exclusion criteria) from the protocol. PP set will be used for endpoint analysis.

The primary efficacy analysis will be performed in the FAS. This analysis will be repeated in PPS to support the primary results.

**Safety set (SS):** Population will consist of subjects who are successfully randomized, received at least one dose of study medications and who have data collected at any time after randomization until the end of the study. The safety analysis will be performed in SS.

**10.3 Methods of statistical analyses**

Continuous variables will be summarized as the number of observations, number of missing values, mean, standard deviation, median, quartiles, and range. Categorical variables will be summarized as the number of observations, number of missing values, frequencies, and percentages. Baseline clinical, demographic, laboratory and procedural characteristics will be summarized by randomized treatment group.

All statistical analyses are performed using SPSS software, Version 22. For comparison between the two groups, all hypothesis tests are conducted by two-side test, α=0.05.

The efficacy analysis will be conducted according to the intention-to-treat (ITT) principle. Kaplan-Meier curves and log-rank test will be used to analyze and compare the timing of the first event in each group. P <0.05 is statistically significant. The reliability of all confidence intervals (CI) is 95%, and Cox proportional risk regression model is used to determine the hazard ratio (HR).

**10.4 Subgroup analyses**

The primary and secondary endpoints will be also analyzed in the following clinically relevant pre-specified subgroups.

- Age (<65 years, ≥65 years)

- Sex (male, female)

- BMI (<25kg/m^2^, ≥25kg/m^2^)

- Killip class (class I, II, III, IV)

- Diabetes mellitus (yes, no)

- Renal function (eGFR<60, ≥60 mL/min/1.73m^2^)

- Anemia (hemoglobin <12.0 g/dL in women and <13.0 g/dL in men; yes, no)

- Type of P2Y12 inhibitors (ticagrelor, clopidogrel)

- Any other anticoagulant treatment after PCI (yes, no)

- GRACE score (<140, ≥140)

- OPT-CAD score (<90, ≥90)

- CRUSADE score (<30, ≥30)

- First medical contact to PCI time (≤90 mins, > 90 mins)

- Access site (radial, femoral)

- Multivessel disease (yes, no)

- GPI during PCI (yes, no)

- Left main/Proximal LAD disease (yes, no)

- Circulation assist device (yes, no)

- No/slow-reflow (yes, no)

- ACT met standard after first bolus injection (yes, no, not performed)

All stratified analyses will be accompanied by a test for interaction between treatment effect and stratification factor.

**11. QUALITY CONTROL**

Sponsor shall appoint monitors to conduct systematic monitoring for the study in accordance with GCP principles to ensure the study is carried out according to the protocol and the case report form is identical with original data. The monitors should also assess compliance corresponding to the regulations and protocol. Investigators must ensure the integrity of the medical files. All study files will be reviewed by the sponsor and monitors. Monitors should ensure the following: the rights of the subjects should be protected; original data should comply with GCP and protocol requirements.

**12. CEC**

A CEC is organized for this study. The committee is composed of a number of interventional cardiologists who are not participants in this study. The committee shoulders the mission for the classification of clinical events and the development of specific criteria of clinical endpoints used in this study. The CEC will request the original data if necessary. All CEC members are unaware of the type of study medication subjects receive. All events are judged based on ARC, BARC and other standard definitions (Appendix 1). The Committee shall regularly make assessments and judgments on:

- Death;
- Myocardial infarction (MI);
- Ischemia-driven target vessel revascularization;
- ST;
- Bleeding;
- Stroke;
- Thrombocytopenia;
- Others.

**13. EC**

The investigator is responsible submitting the study protocol, informed consent form and other related study documents to the EC. The investigator should also meet all the requirements to obtain approval for conducting this clinical trial from EC. During the study, the sponsor or contract research organization appointed by the sponsor should promptly report SAEs, including risks to subjects and other issues to EC.

**14. STUDY MANAGEMENT**

**14.1 Modification in the protocol**

Any modification in the protocol should be released by the sponsor and signed and dated by the investigators. The updated protocol should be saved as an annex. Modifications that do not impact subjects’ interests and study outcome assessment, after identification from the Primary Investigator, could apply for exemption of approval from EC. Any other modifications to protocol should be approved and recorded by EC. If deviations from the protocol are necessary, investigators should inform the sponsor as soon as possible, and discuss the specific situation to reach an agreement. The reason for deviation from the protocol should be recorded. If the telephone or address is changed during the study, the sponsor will inform investigators in writing without modification to protocol.

**14.2 CRF tracking**

All CRFs of subjects who signed an informed consent should be turned over regardless of whether the study is completed. Any questions or comments on the CRF must be submitted directly to the sponsors and CRA.

**14.3 Training**

Sponsor must ensure that all staff involved in the study have been trained by the sponsor or the organization designated by sponsor before the study starts. An investigators’ meeting will be convened for all investigators to be familiarized with the protocol, CRF, and distribution of study medications, etc.

**14.4 Replacement of research site**

Research centers which are replaced must be documented. The only reasons to replace a research center are slow enrollment and poor compliance.

**14.5 Follow-up and medical measures at the end of study**

All AEs and SAEs (including laboratory abnormalities) that are unsolved at the end of study or when the subject drops out early should be followed up; see details in the relevant sections about AEs.

**15. RESPOSIBILITIES OF EACH PARTY**

**15.1 Responsibilities of sponsor**

1. Design the study protocol and sign contracts with the investigators and contract research organization;

2. Provide instruction of the drug used in the study to the investigators and monitors;

3. Provide relevant training to the investigators before study initiation;

4. Collect and keep clinical data and information;

5. Collect and keep relevant information, such as clinical programs, medical records, the EC opinions, adverse event reports, statistical analysis, basic data and final clinical report;

6. Report side effects to the administration;

7. Provide rights and ability to terminate the study.

**15.2 Responsibilities of investigators**

1. Be familiar with and strictly follow the study protocol;

2. Complete the clinical study within the stipulated time, including 30-day, 6-month, and 12-month clinic and telephone follow-up;

3. Submit the study protocol to local EC and update progress to sponsor;

4. Be familiar with relevant data (nature, efficacy and safety of the study drug), and newest information associated with the study drug, which is reported during the course of the study;

5. Conduct the clinical trial in qualified medical institutions capable of handling emergency situations to ensure the safety of subjects; laboratory test results should be accurate and reliable;

6. Obtain consent from the medical institutions, and ensure the clinical study is completed within the stipulated time. Investigators should ensure that all stuff members are familiar with study materials, rules and their responsibilities, in order to enroll a sufficient number of subjects in this trial;

7. Ensure that subjects are familiar with detailed information of study drugs, have enough time to consider participating this study, and sign the informed consent forms prior to study initiation;

8. If adverse events occur, investigators must immediately take appropriate therapeutic measures for the subjects, and report the events to the EC, the sponsor and collaborative organizations in a timely fashion. If SAEs occur, the sponsor and investigator should immediately analyze SAEs, take necessary measures to protect safety and interests of subjects, promptly report to the Primary Investigator and the drug supervision and administration department, and also inform other investigators who are in charge of clinical trials involving the same drug;

9. Ensure that the data is true, accurate, complete, reported in a timely fashion, and legally recorded in the medical files and CRF;

10. Accept the monitoring and auditing of the monitors as well as inspection of the drug supervision and management department so as to ensure the quality of the clinical study;

11. Ensure no conflict exists with other clinical studies;

12. The interests of the subjects are the first priority in any situation. Protecting subjects from adverse events is consistent with the protocol, but any AEs and deviations from the protocol should be described in the final report;

13. Any modification (for subjects’ safety) that deviate from the protocol should be reported to the EC and the sponsor;

14. If unexpected events occur, investigators should terminate the clinical study and notify the subjects and their doctors;

15. Take primary responsibility to ensure the validity, clarity and reliability of all documents relating to the study;

16. Any modification to the original data must be signed by an authorized person and dated, and the original records must be retained for future reference;

17. Ensure the retention of original data for a certain period of time (as required by national laws and regulations). After completion of the follow-up for the study, submit a final study report to the sponsor, and take legal responsibility for the accuracy, clarity and reliability of the report;

18. Maintain confidentiality obligations regarding information provided by the sponsor during the entire process of the study.

**15.3 Responsibilities of CRO**

1. Monitor the entire process of the study;

2. Promptly update any adverse events or deviations to the sponsor and investigators;

3. Follow the protocol and report any variances from the protocol to the sponsor in writing, and reach consensus with the sponsor;

4. Use study drugs strictly according to the protocol, and report any deviations to the sponsor;

5. Ensure that personnel and facilities conduct clinical trials safely and effectively;

6. Conduct systematic monitoring of the study in accordance with GCP principles;

7. Ensure informed consent of the subjects has been obtained;

8. In accordance with national regulations, the case record form should be recorded on time and consistent with the data from the subject;

9. Record and report any adverse events to the sponsor;

10. Retain records for non-compliance of drug prescription and any study terminations from subjects;

11. After completion of the follow-up for the study, submit a final study report to the sponsor and investigators, and accept legal responsibility for the accuracy, clarity and reliability of the report;

12. Maintain confidentiality obligations regarding information provided by the sponsor during the entire process of the study.

**16. REFERENCES**

1. Steg PG, van 't Hof A, Hamm CW, Clemmensen P, Lapostolle F, Coste P, et al. Bivalirudin started during emergency transport for primary PCI. N Engl J Med. 2013;369(23):2207-17.

2. Gregg W. Stone, Bernhard Witzenbichler, Giulio Guagliumi, Jan Z. Peruga, Bruce R. Brodie, Dariusz Dudek, et al. Bivalirudin during Primary PCI in Acute Myocardial Infarction. N Engl J Med. 2008(358):2218-30.

3. Shahzad A, Kemp I, Mars C, Wilson K, Roome C, Cooper R, et al. Unfractionated heparin versus bivalirudin in primary percutaneous coronary intervention (HEAT-PPCI): an open-label, single centre, randomised controlled trial. The Lancet. 2014;384(9957):1849-58.

4. Cavender MA, Sabatine MS. Bivalirudin versus heparin in patients planned for percutaneous coronary intervention: a meta-analysis of randomised controlled trials. Lancet. 2014;384(9943):599-606.

5. Stone GW, Mehran R, Goldstein P, Witzenbichler B, Van't Hof A, Guagliumi G, et al. Bivalirudin versus heparin with or without glycoprotein IIb/IIIa inhibitors in patients with STEMI undergoing primary percutaneous coronary intervention: pooled patient-level analysis from the HORIZONS-AMI and EUROMAX trials. J Am Coll Cardiol. 2015;65(1):27-38.

6. Han Y, Guo J, Zheng Y, Zang H, Su X, Wang Y, et al. Bivalirudin vs heparin with or without tirofiban during primary percutaneous coronary intervention in acute myocardial infarction: the BRIGHT randomized clinical trial. JAMA. 2015;313(13):1336-46.

7. Ibanez B, James S, Agewall S, Antunes MJ, Bucciarelli-Ducci C, Bueno H, et al. 2017 ESC Guidelines for the management of acute myocardial infarction in patients presenting with ST-segment elevation: The Task Force for the management of acute myocardial infarction in patients presenting with ST-segment elevation of the European Society of Cardiology (ESC). Eur Heart J. 2018;39(2):119-77.

**17. APPENDIX 1 - DEFINITION**

**Death:** Deaths that are not caused by definite non-cardiac factors are deemed to be cardiac deaths. Specifically, any unexpected deaths in subjects are deemed to be cardiac deaths, even if they also have potential fatal non-cardiac diseases (for example, cancer and infection).

**Cardiac death:** Any deaths without known cause, and deaths caused by immediate heart-related factors (such as MI, low cardiac output heart failure or fatal arrhythmia) are deemed to be cardiac deaths, including those related to surgery and accompanied treatment.

**Vascular death:** Deaths caused by cerebrovascular diseases, pulmonary embolism, aneurysm rupture or other vascular diseases.

**Non-cardiovascular death:** Any deaths that are not covered in the definitions above, including those caused by infection, pyemia, pulmonary diseases, accident, suicide or injury.

**Myocardial infarction.** Myocardial infarction is defined according to the fourth Universal Definition of Myocardial Infarction.

**Type 1:** Detection of a rise and/or fall of cardiac troponin (cTn) values with at least 1 value above the 99th percentile upper reference limit (URL) and with at least 1 of the following:

- Symptoms of acute myocardial ischemia;
- Myocardial infarction (MI);
- New ischemic ECG changes;
- Development of pathological Q waves;
- Imaging evidence of new loss of viable myocardium or new regional wall motion abnormality in a pattern consistent with an ischemic etiology;
- Identification of a coronary thrombus by angiography including intracoronary imaging or by autopsy*.

*Postmortem demonstration of an atherothrombus in the artery supplying the infarcted myocardium, or a macroscopically large circumscribed area of necrosis with or without intramyocardial hemorrhage, meets the type 1 MI criteria regardless of cTn values.

Type 2: Detection of a rise and/or fall of cTn values with at least 1 value above the 99th percentile URL, and evidence of an imbalance between myocardial oxygen supply and demand unrelated to acute coronary atherothrombosis, requiring at least 1 of the following:

- Symptoms of acute myocardial ischemia;
- New ischemic ECG changes;
- Development of pathological Q waves;
- Imaging evidence of new loss of viable myocardium or new regional wall motion abnormality in a pattern consistent with an ischemic etiology.

**Type 3:** Patients who suffer cardiac death, with symptoms suggestive of myocardial ischemia accompanied by presumed new ischemic ECG changes or ventricular fibrillation, but die before blood samples for biomarkers can be obtained, or before increases in cardiac biomarkers can be identified, or MI is detected by autopsy examination.

**Type 4a:** Coronary intervention–related MI is arbitrarily defined by an elevation of cTn values >5 times the 99th percentile URL in patients with normal baseline values. In patients with elevated preprocedural cTn in whom the cTn level are stable (≤20% variation) or falling, the postprocedural cTn must rise by >20%. However, the absolute postprocedural value must still be at least 5 times the 99th percentile URL. In addition, 1 of the following elements is required:

- New ischemic ECG changes;
- Development of pathological Q waves*;
- Imaging evidence of new loss of viable myocardium or new regional wall motion abnormality in a pattern consistent with an ischemic etiology;
- Angiographic findings consistent with a procedural flow-limiting complication such as coronary dissection, occlusion of a major epicardial artery or a side branch occlusion/thrombus, disruption of collateral flow, or distal embolization†.

*Isolated development of new pathological Q waves meets the type 4a MI criteria if cTn values are elevated and rising but <5 times the 99th percentile URL.

†Postmortem demonstration of a procedure-related thrombus in the culprit artery, or a macroscopically large circumscribed area of necrosis with or without intra-myocardial hemorrhage meets the type 4a MI criteria.

**Type 4b:** A subcategory of PCI-related MI is stent/scaffold thrombosis, type 4b MI, as documented by angiography or autopsy using the same criteria utilized for type 1 MI. It is important to indicate the time of the occurrence of the stent/scaffold thrombosis in relation to the timing of the PCI procedure. The following temporal categories are suggested: acute, 0 to 24 hours; subacute, >24 hours to 30 days; late, >30 days to 1 year; and very late >1 year after stent/scaffold implantation.

**Type 4c:** Occasionally MI occurs and—at angiography, in-stent restenosis, or restenosis following balloon angioplasty in the infarct territory—is the only angiographic explanation since no other culprit lesion or thrombus can be identified. This PCI-related MI type is designated as type 4c MI, defined as focal or diffuse restenosis, or a complex lesion associated with a rise and/or fall of cTn values above the 99th percentile URL applying, the same criteria utilized for type 1 MI.

**Type 5:** CABG-related MI is arbitrarily defined as elevation of cTn values >10 times the 99th percentile URL in patients with normal baseline cTn values. In patients with elevated preprocedural cTn in whom cTn levels are stable (≤20% variation) or falling, the postprocedural cTn must rise by >20%. However, the absolute postprocedural value still must be >10 times the 99th percentile URL. In addition, 1 of the following elements is required:

- Development of pathological Q waves*;
- Angiographic documentation of a new graft occlusion or new native coronary artery occlusion;
- Imaging evidence of new loss of viable myocardium or new regional wall motion abnormality in a pattern consistent with an ischemic etiology.

*Isolated development of new pathological Q waves meets the type 5 MI criteria if cTn values are elevated and rising but <10 times the 99th percentile URL.

**Recurrent myocardial infarction:** Incident MI is defined as the individual’s first MI. When features of MI occur in the first 28 days after an incident event, the second event is not counted as a new MI for epidemiological purposes. If characteristics of MI occur after 28 days following an incident MI, it is considered to be a recurrent MI.

**Revascularization**

**Target lesion revascularization (TLR):** TLR is defined as any repeat percutaneous intervention of the target lesion or bypass surgery of the target vessel performed for restenosis or other complication of the target lesion. All TLRs should be classified prospectively as clinically indicated or not clinically indicated by the investigator prior to repeat angiography. An independent angiographic core laboratory should verify that the severity of percent diameter stenosis meets requirements for clinical indication and will overrule in cases where investigator reports are not in agreement. The target lesion is defined as the treated segment from 5 mm proximal to the stent and to 5 mm distal to the stent.

A revascularization is considered clinically indicated if angiography at follow-up shows a percent diameter stenosis ≥50% (core lab QCA assessment) and if one of the following occurs: (1) a positive history of recurrent angina pectoris, presumably related to the target vessel; (2) objective signs of ischemia at rest (ECG changes) or during exercise test (or equivalent), presumably related to the target vessel; (3) abnormal results of any invasive functional diagnostic test (eg, Doppler flow velocity reserve, fractional flow reserve); (4) A TLR or TVR with a diameter stenosis ≥70% even in the absence of the above-mentioned ischemic signs or symptoms.

**Target vessel revascularization (TVR):** TVR is defined as any repeat percutaneous intervention or surgical bypass of any segment of the target vessel. The target vessel is defined as the entire major coronary vessel proximal and distal to the target lesion, which includes upstream and downstream branches and the target lesion itself.

**Non-TLR:** Non-TLR is defined as any repeat percutaneous intervention or bypass surgery of any other lesion except for target lesion.

**Non-TVR:** Non-TVR is defined as any repeat percutaneous intervention or surgical bypass of any segment of other vessels except for target vessel.

**Ischemia-driven TVR.** An ischemia-driven TVR is defined if angiography at follow-up shows a percent diameter stenosis ≥50% (core lab QCA assessment) and if one of the following occurs:

1. a positive history of recurrent angina pectoris, presumably related to the target vessel;

2. objective signs of ischemia at rest (ECG changes) or during exercise test (or equivalent), presumably related to the target vessel;

3. abnormal results of any invasive functional diagnostic test (eg, Doppler flow velocity reserve, fractional flow reserve);

4. A TLR or TVR with a diameter stenosis ≥70% even in the absence of the above-mentioned ischemic signs or symptoms.

**Stent thrombosis (ST)**

ST should be reported as a cumulative value over time and at the various individual time points specified above. Time 0 is defined as the time point after the guiding catheter has been removed and the patient has left the catheter laboratory.

| Acute stent thrombosis | 0 to 24 hours after stent implantation |
| --- | --- |
| Subacute stent thrombosis | >24 hours to 30 days after stent implantation |
| Late stent thrombosis | >30 days to 1 year after stent implantation |
| Very late stent thrombosis | >1 year after stent implantation |

**Classification of ST:**

**1. Definite ST.** Angiographic confirmation of stent thrombosis. The presence of a thrombus that originates in the stent or in the segment 5 mm proximal or distal to the stent and presence of at least 1 of the following criteria within a 48-hour time window:

- Typical angina pectoris symptoms lasting for over 20 minutes;
- New ischemic ECG changes that suggest acute ischemia;
- Typical rise and fall in cardiac biomarkers (refer to definition of spontaneous MI).

**2. Probable ST.** Unexplained death within 30 days after the procedure, or acute myocardial infarction involving the target-vessel territory without angiographic confirmation.

**3. Possible ST.** All unexplained death occurring at least 30 days after the procedure.

**Stroke.** Stroke is defined as an acute event of non-hemorrhagic cerebrovascular origin causing focal or global neurologic dysfunction lasting > 24 hours, which is confirmed by both clinical and radiographic criteria.

**Definition of BARC bleeding**

| **Type** | **Definition** |
| --- | --- |
| 0 | No evidence of bleeding. |
| 1 | Bleeding that is not actionable and patient does not have unscheduled studies,  hospitalization or treatment by a health care professional. |
| 2 | Any clinically overt sign of hemorrhage that is actionable but does not meet criteria for type 3, 4 or 5 bleeding. It must meet at least one of the following criteria:  1) requiring medical or percutaneous intervention guided by a health care profession, includes (but are not limited to) temporary/permanent cessation of a medication, coiling, compression, local injection;  2) leading to hospitalization or an increased level of care;  3) prompting evaluation defined as an unscheduled visit to a healthcare professional resulting in diagnostic testing (laboratory or imaging). |
| 3 | Clinical, laboratory and/or imaging evidence of bleeding with specific healthcare  provider responses, as listed below: |
| 3a | 1) Any transfusion with overt bleeding;  2) Overt bleeding plus hemoglobin (Hb) drop ≥3 to <5g/dL * (provided Hb drop is related to bleeding). |
| 3b | 1) Overt bleeding plus Hb drop ≥5g/dL* (Hb drop is related to bleed);  2) Cardiac tamponade;  3) Bleeding requiring surgical intervention for control (excluding dental/nasal/skin/hemorrhoid);  4) Bleeding requiring intravenous vasoactive drugs. |
| 3c | 1) Intracranial hemorrhage (does not include microbleeds or hemorrhagic transformation; does include intraspinal). Subcategories: confirmed by autopsy, imaging or lumbar puncture;  2) Intraocular bleed compromising vision. |
| 4 | CABG – Related Bleeding  1) Perioperative intracranial bleeding within 48 hours;  2) Reoperation following closure of sternotomy for the purpose of controlling bleeding;  3) Transfusion of ≥5 units of whole blood or packed red blood cells within a 48-hour period;  4) Chest tube output ≥2L within a 24-hour period. |
| 5 | Fatal Bleeding. Bleeding directly causes death with no other explainable cause.  Categorized further as either definite or probable. |
| 5a | Probable fatal bleeding is bleeding that is clinically suspicious as the cause of death, but the bleeding is not directly observed and there is no autopsy or confirmatory imaging. |
| 5b | Definite fatal bleeding is bleeding that is directly observed (either by clinical specimen – blood, emesis, stool, etc. – or by imaging) or confirmed on autopsy. |

**Thrombocytopenia.** Thrombocytopenia is defined as the lowest platelet count <150×10^9^/L for patients with baseline platelet count >150×10^9^/L after treatment.
