# Supplementary material for: Analysis of inter-hospital transfer on clinical outcomes after primary percutaneous coronary intervention for ST-segment elevation myocardial infarction: A secondary analysis of the BRIGHT-4 trial
Source: PLoS Med. 2025 Jul 23;22(7):e1004679. doi: 10.1371/journal.pmed.1004679 (PMC12313069; doi:10.1371/journal.pmed.1004679)
Supplement: S3 Text — (DOCX) [file pmed.1004679.s014.docx]

S3 Text. Statistical analysis plan

**STATISTICAL ANALYSIS PLAN**

**BivaliRudin with prolonged high-dose Infusion durinG Primary PCI versus Heparin Trial**

**(The BRIGHT-4 Trial)**

Version 1.1

30-May-2021

**CONTENTS**

**[ACRONYMS AND ABBREVIATIONS](file:///C:\\Users\\momo\\Desktop\\transferred-in%20and%20direct%20admission\\direct%20admission%20vs.%20inter-hospital%20transfer\\投稿6\\upload-revision\\PLOS修稿\\BRIGHT-4%20Supplementary_Appendix%20revised%20clean.docx#_Toc101859045)**

**[1 INTRODUCTION](file:///C:\\Users\\momo\\Desktop\\transferred-in%20and%20direct%20admission\\direct%20admission%20vs.%20inter-hospital%20transfer\\投稿6\\upload-revision\\PLOS修稿\\BRIGHT-4%20Supplementary_Appendix%20revised%20clean.docx#_Toc101859046)**

[1.1 Preface](file:///C:\\Users\\momo\\Desktop\\transferred-in%20and%20direct%20admission\\direct%20admission%20vs.%20inter-hospital%20transfer\\投稿6\\upload-revision\\PLOS修稿\\BRIGHT-4%20Supplementary_Appendix%20revised%20clean.docx#_Toc101859047)

**[2 OBJECTIVES](file:///C:\\Users\\momo\\Desktop\\transferred-in%20and%20direct%20admission\\direct%20admission%20vs.%20inter-hospital%20transfer\\投稿6\\upload-revision\\PLOS修稿\\BRIGHT-4%20Supplementary_Appendix%20revised%20clean.docx#_Toc101859048)**

[2.1 Primary Objective](file:///C:\\Users\\momo\\Desktop\\transferred-in%20and%20direct%20admission\\direct%20admission%20vs.%20inter-hospital%20transfer\\投稿6\\upload-revision\\PLOS修稿\\BRIGHT-4%20Supplementary_Appendix%20revised%20clean.docx#_Toc101859049)

[2.2 Secondary Objectives](file:///C:\\Users\\momo\\Desktop\\transferred-in%20and%20direct%20admission\\direct%20admission%20vs.%20inter-hospital%20transfer\\投稿6\\upload-revision\\PLOS修稿\\BRIGHT-4%20Supplementary_Appendix%20revised%20clean.docx#_Toc101859050)

**[3 STUDY DESIGN](file:///C:\\Users\\momo\\Desktop\\transferred-in%20and%20direct%20admission\\direct%20admission%20vs.%20inter-hospital%20transfer\\投稿6\\upload-revision\\PLOS修稿\\BRIGHT-4%20Supplementary_Appendix%20revised%20clean.docx#_Toc101859051)**

[3.1 Overall study design and flow chart](file:///C:\\Users\\momo\\Desktop\\transferred-in%20and%20direct%20admission\\direct%20admission%20vs.%20inter-hospital%20transfer\\投稿6\\upload-revision\\PLOS修稿\\BRIGHT-4%20Supplementary_Appendix%20revised%20clean.docx#_Toc101859052)

[3.2 Inclusion/Exclusion criteria](file:///C:\\Users\\momo\\Desktop\\transferred-in%20and%20direct%20admission\\direct%20admission%20vs.%20inter-hospital%20transfer\\投稿6\\upload-revision\\PLOS修稿\\BRIGHT-4%20Supplementary_Appendix%20revised%20clean.docx#_Toc101859053)

[3.3 Subject randomization](file:///C:\\Users\\momo\\Desktop\\transferred-in%20and%20direct%20admission\\direct%20admission%20vs.%20inter-hospital%20transfer\\投稿6\\upload-revision\\PLOS修稿\\BRIGHT-4%20Supplementary_Appendix%20revised%20clean.docx#_Toc101859054)

[3.4 Blinding](file:///C:\\Users\\momo\\Desktop\\transferred-in%20and%20direct%20admission\\direct%20admission%20vs.%20inter-hospital%20transfer\\投稿6\\upload-revision\\PLOS修稿\\BRIGHT-4%20Supplementary_Appendix%20revised%20clean.docx#_Toc101859055)

[3.5 Study medications and Treatments](file:///C:\\Users\\momo\\Desktop\\transferred-in%20and%20direct%20admission\\direct%20admission%20vs.%20inter-hospital%20transfer\\投稿6\\upload-revision\\PLOS修稿\\BRIGHT-4%20Supplementary_Appendix%20revised%20clean.docx#_Toc101859056)

[3.5.1 Bivalirudin group](file:///C:\\Users\\momo\\Desktop\\transferred-in%20and%20direct%20admission\\direct%20admission%20vs.%20inter-hospital%20transfer\\投稿6\\upload-revision\\PLOS修稿\\BRIGHT-4%20Supplementary_Appendix%20revised%20clean.docx#_Toc101859057)

[3.5.2 Heparin group](file:///C:\\Users\\momo\\Desktop\\transferred-in%20and%20direct%20admission\\direct%20admission%20vs.%20inter-hospital%20transfer\\投稿6\\upload-revision\\PLOS修稿\\BRIGHT-4%20Supplementary_Appendix%20revised%20clean.docx#_Toc101859058)

[3.5.3 Concomitant medications](file:///C:\\Users\\momo\\Desktop\\transferred-in%20and%20direct%20admission\\direct%20admission%20vs.%20inter-hospital%20transfer\\投稿6\\upload-revision\\PLOS修稿\\BRIGHT-4%20Supplementary_Appendix%20revised%20clean.docx#_Toc101859059)

[3.6 Study variables](file:///C:\\Users\\momo\\Desktop\\transferred-in%20and%20direct%20admission\\direct%20admission%20vs.%20inter-hospital%20transfer\\投稿6\\upload-revision\\PLOS修稿\\BRIGHT-4%20Supplementary_Appendix%20revised%20clean.docx#_Toc101859060)

[3.6.1 Study timetable and end of study](file:///C:\\Users\\momo\\Desktop\\transferred-in%20and%20direct%20admission\\direct%20admission%20vs.%20inter-hospital%20transfer\\投稿6\\upload-revision\\PLOS修稿\\BRIGHT-4%20Supplementary_Appendix%20revised%20clean.docx#_Toc101859061)

**[4 PRIMARY AND SECONDARY VARIABLES](file:///C:\\Users\\momo\\Desktop\\transferred-in%20and%20direct%20admission\\direct%20admission%20vs.%20inter-hospital%20transfer\\投稿6\\upload-revision\\PLOS修稿\\BRIGHT-4%20Supplementary_Appendix%20revised%20clean.docx#_Toc101859062)**

[4.1 Primary variable](file:///C:\\Users\\momo\\Desktop\\transferred-in%20and%20direct%20admission\\direct%20admission%20vs.%20inter-hospital%20transfer\\投稿6\\upload-revision\\PLOS修稿\\BRIGHT-4%20Supplementary_Appendix%20revised%20clean.docx#_Toc101859063)

[4.2 Secondary variables](file:///C:\\Users\\momo\\Desktop\\transferred-in%20and%20direct%20admission\\direct%20admission%20vs.%20inter-hospital%20transfer\\投稿6\\upload-revision\\PLOS修稿\\BRIGHT-4%20Supplementary_Appendix%20revised%20clean.docx#_Toc101859064)

[4.3 Safety variables](file:///C:\\Users\\momo\\Desktop\\transferred-in%20and%20direct%20admission\\direct%20admission%20vs.%20inter-hospital%20transfer\\投稿6\\upload-revision\\PLOS修稿\\BRIGHT-4%20Supplementary_Appendix%20revised%20clean.docx#_Toc101859065)

**[5 SAMPLE SIZE](file:///C:\\Users\\momo\\Desktop\\transferred-in%20and%20direct%20admission\\direct%20admission%20vs.%20inter-hospital%20transfer\\投稿6\\upload-revision\\PLOS修稿\\BRIGHT-4%20Supplementary_Appendix%20revised%20clean.docx#_Toc101859066)**

**[6 ANALYSIS SETS](file:///C:\\Users\\momo\\Desktop\\transferred-in%20and%20direct%20admission\\direct%20admission%20vs.%20inter-hospital%20transfer\\投稿6\\upload-revision\\PLOS修稿\\BRIGHT-4%20Supplementary_Appendix%20revised%20clean.docx#_Toc101859067)**

[6.1 Full analysis set (FAS)](file:///C:\\Users\\momo\\Desktop\\transferred-in%20and%20direct%20admission\\direct%20admission%20vs.%20inter-hospital%20transfer\\投稿6\\upload-revision\\PLOS修稿\\BRIGHT-4%20Supplementary_Appendix%20revised%20clean.docx#_Toc101859068)

[6.2 Per protocol set (PPS)](file:///C:\\Users\\momo\\Desktop\\transferred-in%20and%20direct%20admission\\direct%20admission%20vs.%20inter-hospital%20transfer\\投稿6\\upload-revision\\PLOS修稿\\BRIGHT-4%20Supplementary_Appendix%20revised%20clean.docx#_Toc101859069)

[6.3 Safety analysis set](file:///C:\\Users\\momo\\Desktop\\transferred-in%20and%20direct%20admission\\direct%20admission%20vs.%20inter-hospital%20transfer\\投稿6\\upload-revision\\PLOS修稿\\BRIGHT-4%20Supplementary_Appendix%20revised%20clean.docx#_Toc101859070)

**[7 SUBGROUPS](file:///C:\\Users\\momo\\Desktop\\transferred-in%20and%20direct%20admission\\direct%20admission%20vs.%20inter-hospital%20transfer\\投稿6\\upload-revision\\PLOS修稿\\BRIGHT-4%20Supplementary_Appendix%20revised%20clean.docx#_Toc101859071)**

**[8 LOST TO FOLLOW-UP AND MISSING DATA](file:///C:\\Users\\momo\\Desktop\\transferred-in%20and%20direct%20admission\\direct%20admission%20vs.%20inter-hospital%20transfer\\投稿6\\upload-revision\\PLOS修稿\\BRIGHT-4%20Supplementary_Appendix%20revised%20clean.docx#_Toc101859072)**

**[9 STATISTICAL METHODS](file:///C:\\Users\\momo\\Desktop\\transferred-in%20and%20direct%20admission\\direct%20admission%20vs.%20inter-hospital%20transfer\\投稿6\\upload-revision\\PLOS修稿\\BRIGHT-4%20Supplementary_Appendix%20revised%20clean.docx#_Toc101859073)**

[9.1 General principles](file:///C:\\Users\\momo\\Desktop\\transferred-in%20and%20direct%20admission\\direct%20admission%20vs.%20inter-hospital%20transfer\\投稿6\\upload-revision\\PLOS修稿\\BRIGHT-4%20Supplementary_Appendix%20revised%20clean.docx#_Toc101859074)

[9.2 Analysis for the primary endpoint](file:///C:\\Users\\momo\\Desktop\\transferred-in%20and%20direct%20admission\\direct%20admission%20vs.%20inter-hospital%20transfer\\投稿6\\upload-revision\\PLOS修稿\\BRIGHT-4%20Supplementary_Appendix%20revised%20clean.docx#_Toc101859075)

[9.3 Analysis for the secondary endpoints](file:///C:\\Users\\momo\\Desktop\\transferred-in%20and%20direct%20admission\\direct%20admission%20vs.%20inter-hospital%20transfer\\投稿6\\upload-revision\\PLOS修稿\\BRIGHT-4%20Supplementary_Appendix%20revised%20clean.docx#_Toc101859076)

[9.4 Safety analysis](file:///C:\\Users\\momo\\Desktop\\transferred-in%20and%20direct%20admission\\direct%20admission%20vs.%20inter-hospital%20transfer\\投稿6\\upload-revision\\PLOS修稿\\BRIGHT-4%20Supplementary_Appendix%20revised%20clean.docx#_Toc101859077)

[9.5 Subgroup analysis](file:///C:\\Users\\momo\\Desktop\\transferred-in%20and%20direct%20admission\\direct%20admission%20vs.%20inter-hospital%20transfer\\投稿6\\upload-revision\\PLOS修稿\\BRIGHT-4%20Supplementary_Appendix%20revised%20clean.docx#_Toc101859078)

[9.6 Sensitivity analyses](file:///C:\\Users\\momo\\Desktop\\transferred-in%20and%20direct%20admission\\direct%20admission%20vs.%20inter-hospital%20transfer\\投稿6\\upload-revision\\PLOS修稿\\BRIGHT-4%20Supplementary_Appendix%20revised%20clean.docx#_Toc101859079)

**[10 TABLE LISTINGS](file:///C:\\Users\\momo\\Desktop\\transferred-in%20and%20direct%20admission\\direct%20admission%20vs.%20inter-hospital%20transfer\\投稿6\\upload-revision\\PLOS修稿\\BRIGHT-4%20Supplementary_Appendix%20revised%20clean.docx#_Toc101859080)**

**[11 REFERENCES](file:///C:\\Users\\momo\\Desktop\\transferred-in%20and%20direct%20admission\\direct%20admission%20vs.%20inter-hospital%20transfer\\投稿6\\upload-revision\\PLOS修稿\\BRIGHT-4%20Supplementary_Appendix%20revised%20clean.docx#_Toc101859081)**

ACRONYMS AND ABBREVIATIONS

| **Acronym/Abbreviation** | **Term** |
| --- | --- |
| ACT | activated clotting time |
| AE | Adverse event |
| ALT | alanine aminotransferase |
| ARC | Academic Research Consortium |
| BARC | Bleeding academic research consortium |
| BRIGHT | BivaliRudin with prolonged high-dose Infusion durinG primary PCI versus Heparin Trial |
| CEC | clinical event committee |
| CI | confidence interval |
| CRF | case report form |
| EDC | electronic data capture |
| eGFR | estimated Glomerular filtration rate |
| FAS | Full analysis set |
| GPI | GP IIb/IIIa inhibitor |
| HR | Hazard ratio |
| ITT | intent-to-treat |
| LBBB | left bundle branch block |
| LMWH | low molecular weight heparin |
| MACCE | major adverse cardiac and cerebral event |
| NACE | net adverse clinical event |
| OR | odd ratio |
| PCI | percutaneous coronary intervention |
| PPS | per protocol set |
| SAE | serious adverse event |
| SAP | statistical analysis plan |
| SS | safety set |
| STEMI | ST-segment elevation myocardial infarction |
| ST | stent thrombosis |

**1 INTRODUCTION**

This document contains the Statistical Analysis Plan (SAP) for BRIGHT-4 trial, protocol Version 1.10.

**1.1 Preface**

Bivalirudin, a direct thrombin inhibitor, is a synthetic hirudin derivative with 20 amino acids. Bivalirudin directly and specifically binds and inhibits thrombin, significantly prolongs the activated clotting time (ACT), and thus provides an anticoagulant effect for preventing thrombosis. Bivalirudin rapidly and reversely effects result in a low bleeding risk, and has a better safety profile in comparison with traditional heparin. Thrombin induces platelet activation, leading to platelet aggregation and granule secretion. Therefore, bivalirudin may also indirectly suppress platelet activation through inhibiting thrombin. In recent years abundant studies have demonstrated that bivalirudin decreases bleeding events for patients undergoing primary percutaneous coronary intervention (PCI) compared with heparin, with comparable antithrombotic efficacy, and may even reduce the risk of cardiac death [1,2]. However, some clinical trials showed that the risk of stent thrombosis (ST) was elevated with bivalirudin among patients undergoing primary PCI [3], and several meta-analyses also had consistent findings [4,5].

This potential detrimental risk of bivalirudin may be associated with its administration strategy. The half-life of bivalirudin is approximately 25 min. Patients with ST segment elevation myocardial infarction (STEMI) have a delayed bioavailability of P2Y12 receptor inhibitors including both traditional clopidogrel and the novel potent agents ticagrelor and prasugrel, not reaching their maximum platelet inhibition effects until 6-8 hours after administration. Therefore, a “window period” lack of antithrombotic effect may exist if the bivalirudin infusion is stopped immediately in the cath lab as in the early clinical trials in primary PCI, with patients in a hypercoagulable state. A meta-analysis verified the above hypothesis, showing that most bivalirudin-related acute ST events occurred during the first few hours after PCI [4]. To address this issue, our team conducted the BRIGHT study exploring the impact of peri-procedural bivalirudin administration on clinical outcomes in patients undergoing emergency PCI [6]. With 2194 recruited patients from 82 cites, our study pioneered the continuous high-dose (1.75mg/kg/h) bivalirudin maintenance infusion treatment after the procedure and reported that with this high-dose infusion bivalirudin substantially reduces bleeding events without increasing the risk of ST, providing an important benefit for patients undergoing emergency PCI [6].

Based on the BRIGHT study, the current study aims to further compare the safety and efficacy of bivalirudin with a prolonged high-dose infusion versus heparin alone among STEMI patients undergoing primary PCI, with the goal of providing critical clinical evidence to select the optimal peri-procedural anticoagulation strategy for these high-risk patients.

**2 OBJECTIVES**

**2.1 Primary Objective**

The primary study objective is to test the hypothesis that bivalirudin with a prolonged high-dose infusion for 2-4 hours after the procedure is superior to heparin alone during primary PCI in reducing the rate of all-cause death or Bleeding Academic Research Consortium (BARC) types 3-5 bleeding at 30 days (primary endpoint) in STEMI patients.

**2.2 Secondary Objectives**

Secondary objectives include evaluation of net adverse clinical events (a composite of all-cause death, recurrent myocardial infarction, ischemia-driven target vessel revascularization, stroke or BARC types 3-5 bleeding), ST, individual components of the primary endpoint as well as secondary variables listed below (**section 4.2**).

**3 STUDY DESIGN**

**3.1 Overall study design and flow chart**

BRIGHT-4 is a perspective, randomized, open-label, active drug parallel-controlled superiority trial. See Figure 1.


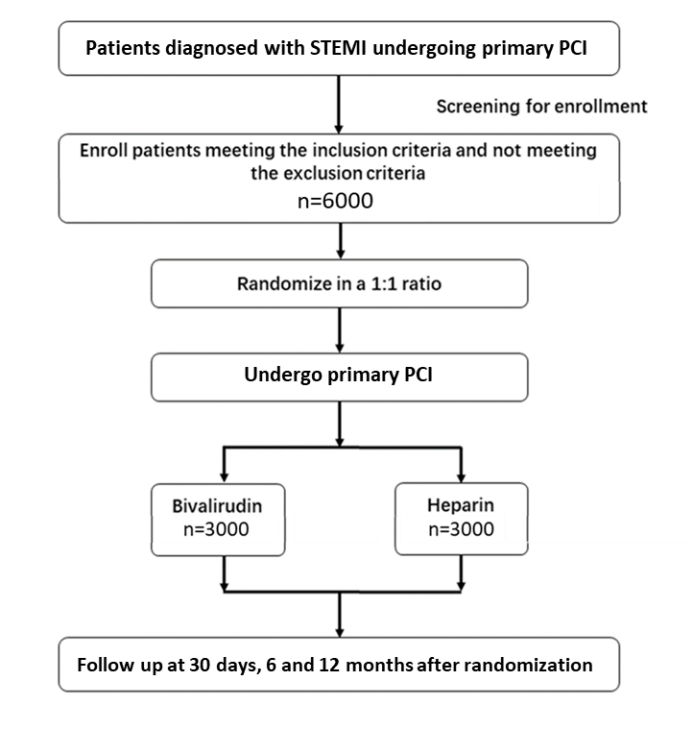


**Figure 1. Study flowchart of the BRIGHT-4 trial**

**3.2 Inclusion/Exclusion criteria**

Enrolment into the study requires meeting all inclusion AND none of the exclusion criteria. Patients meeting all entry criteria will provide informed written consent.

Inclusion criteria are listed below.

(1) Any age;

(2) STEMI patients with symptom onset ≤ 48h with primary PCI planned (STEMI is defined as ST elevation ≥1mm in ≥2 contiguous leads or new LBBB, or persistent ischemic symptoms occur in the presence of RBBB^7^);

(3) Patients requiring staged revascularization of non-culprit vessels within 30 days may also be enrolled. In such cases the antithrombotic agents and procedures in the staged PCI must be consistent with the index PCI, especially the peri-

procedural antithrombotic agents including assigned heparin vs. bivalirudin, and tirofiban);

(4) No contraindications to dual antiplatelet therapy, and dual antiplatelet agents must be administrated according to current guidelines before PCI (loading doses and maintenance doses of aspirin and clopidogrel/ticagrelor);

(5) The subject or legal representatives has been fully informed and written informed consent provided.

Patients may not enter the study if any of the following exclusion criteria are met:

(1) Not suitable for primary PCI as judged by the physicians;

(2) STEMI treated by thrombolysis;

(3) Patients received heparin, LMWH, fondaparinux, bivalirudin, or GP IIb/IIIa inhibitor (GPI) within 48 hours before the index PCI;

(4) Mechanical complications (such as ventricular septal rupture, papillary muscle rupture or acute mitral regurgitation, etc.);

(5) Known allergy or contraindications to heparin, bivalirudin, aspirin, clopidogrel or ticagrelor;

(6) Patients have participated in other drug or device studies;

(7) Patients whom the investigators consider participation in the study to be inappropriate or who may be nonadherent to the study protocol during the follow-up period, such as those with psychiatric disorder, alcoholism or drug addiction.

**3.3 Subject randomization**

Patients meeting ALL the inclusion criteria and NONE of the exclusion criteria will be assigned to the bivalirudin or heparin group via centralized randomization system after signing informed consent.

Patients will be randomized to the bivalirudin or heparin group using a 1:1 allocation ratio with randomly varied block sizes of 4 and 6. The block sizes will not be revealed to sites. The electronic data capture system (EDC) will be used to randomize subjects. The EDC will assign a treatment arm for patients and specify the appropriate medication number for the package of investigational treatment to be dispensed to the patient.

Enrolled patients who do not meet eligibility for randomization will not receive any more study drugs, and subsequent management strategies will be administered at their attending physicians’ discretion in accordance with the local standard of care.

**3.4 Blinding**

The trial program is open-label.

An independent Clinical Events Committee (CEC) will adjudicate all relevant clinical events. The committee members and the CEC management team will be completely blinded to the randomized therapy, as well as to any patient identifying information. The CEC will adjudicate the events based on pre-determined definitions.

The study statistician will be blinded for the primary analyses.

**3.5 Study medications and Treatments**

Study medications were listed in Table 1.

All enrolled STEMI patients will be randomized after providing written consent. Study medication treatment will be administered according to assigned group before angiography in catheterization laboratory. ACT is monitored 5 min after the first administration, and if the ACT is <225 s (Hemotec method), an additional intravenous injection of 0.3 mg/kg of bivalirudin, or 1000 U of heparin according to assigned group is administered to ensure the re-checked ACT is >225 s.

GPI use is strictly limited. Intravenous or intra-coronary tirofiban is only allowed for target vessel slow blood flow, no-reflow, obvious thrombus or thrombosis complication during PCI. At operator’s discretion for the above indications, tirofiban 10-25μg/kg intravenous infusion for loading dose is administered over 5 min, followed by intravenous infusion of 0.15μg/kg/min maintenance dose for up to 18 hours. For patients with eGFR <60ml/min, the maintenance dose is reduced to 0.075μg/kg/min. For intra-coronary infusion, 500-750μg per injection with a 1500-2250 total dose is recommended, with the time interval of 3-5 min between injections.

LWMH after primary PCI is administered at operator’s discretion. Loading doses of aspirin 300mg, and P2Y12 receptor inhibitor clopidogrel 300/600mg or ticagrelor 180mg shall be administered before index PCI. Other medications are prescribed according to current guidelines. Trans-radial (preferred) or -femoral approach, and stent type are at operator’s discretion.

**Table 1. Identity of Study Medications**

| **Study drug** | **The form and dosage of drug** | **Manufacturer** |
| --- | --- | --- |
| Zelang (bivalirudin) | 0.25g/bottle | Jiangsu Hansoh Pharmaceutical Group Co., Ltd. |
| Heparin Sodium Injection (heparin) | 12500U/2ml | Shanghai Pharma No.1 Biochemical Pharmaceutical Co., Ltd. |

**3.5.1 Bivalirudin group**

In the cath lab, bivalirudin 0.75 mg/kg intravenous bolus loading dose is started before angiography, and immediately followed by an intravenous infusion of 1.75 mg/kg/h until 2-4 hours after PCI. ACT is monitored 5 min after the first administration, and if the ACT is <225 s (Hemotec method), an additional intravenous injection of 0.3 mg/kg of bivalirudin is administered to ensure the re-checked ACT is >225 s.

Bivalirudin bolus loading dose is not affected by renal function. Bivalirudin maintenance infusion dose should be reduced to 1.0mg/kg/h for patients with eGFR <30 ml/min and 0.25 mg/kg/h for patients on dialysis.

**3.5.2 Heparin group**

In the cath lab, an intravenous bolus injection of heparin 70 U/kg is given before angiography. ACT is monitored 5 min after the first administration, and if the ACT is <225 s (Hemotec method), an additional intravenous injection of 1000 U of heparin is administered to ensure the re-checked ACT is >225 s.

**3.5.3 Concomitant medications**

Routine use of GPI during procedure is strictly forbidden. Intravenous or intra-coronary tirofiban is only allowed for target vessel slow blood flow, no-reflow, obvious thrombus or thrombosis complication during PCI. If necessary, intravenous tirofiban should be started with a 10-25μg/kg bolus infusion (given over more than 5 min) followed by a 0.15μg/kg/min maintenance infusion for up to 18 hours. For patients with eGFR <60ml/min, the maintenance dose is reduced to 0.075μg/kg/min. Intra-coronary injection of tirofiban should be 500-750μg per injection, with repeated injection intervals of 3-5 min and total dose no more than 1500-2250μg. LWMH after primary PCI can be administered at operator’s discretion.

Clopidogrel 300/600mg or ticagrelor 180mg aspirin for loading dose (LD), followed by clopidogrel 75mg per day or ticagrelor 90mg twice per day for 12-month maintenance dose (MD) is administered. Aspirin 300mg for LD, followed by 100mg per day for long-term MD is administered. Other medications are prescribed according to current guidelines. Trans-radial (preferred) or -femoral approach, and stent type are at operator’s discretion.

**3.6 Study variables**

Data collection commences after the subject has provided informed consent. Data collection including subject demographic information, laboratory tests, and procedural characteristics, medications, as well as follow-up visits or telephone contacts will be conducted by an investigator or site coordinator who has been trained on the protocol and Case Report Forms (CRF). Data required for analysis will be obtained as outlined in Table 2.

**Table 2 Schedule of Data Collection**

| **Study procedures** | **Visit 1** | **Visit 2** | **Visit 3** | **Visit 4** | **Visit 5** |
| --- | --- | --- | --- | --- | --- |
|  | **PCI** | **Discharge** | **30 days** | **6 months** | **12 months** |
| Window Period | 0d |  | ±7d | ±14d | ±30d |
| Eligibility Criteria | X |  |  |  |  |
| Patient Informed Consent | X |  |  |  |  |
| Randomization | X |  |  |  |  |
| Medical History/ Demographics | X |  |  |  |  |
| Vital Signs | X |  |  |  |  |
| Blood and Urine Routine Test^1^ | X |  |  |  |  |
| Blood Biochemical Examination^2^ | X |  |  |  |  |
| Cardiac Markers (including TnT or TNI/CK/CK-MB)^3^ | X |  |  |  |  |
| ACT Test | X |  |  |  |  |
| 12-Leads ECG^4^ | X |  |  |  |  |
| Key Symptom Inspection | X | X | X | X | X |
| Clinical Assessment | X | X | X | X | X |
| Concomitant Medications | X | X | X | X | X |
| Coronary Artery Angiography | X |  |  |  |  |
| Cardiovascular Events | X | X | X | X | X |
| Adverse Events | X | X | X | X | X |

1-4 are routine tests, repeated tests during in-hospital period are at physicians’ discretion.

**3.6.1 Study timetable and end of study**

Trial enrolment of BRIGHT-4 began in January 2019 and the last patient will be enrolled in April 2022. The last patient 30-day follow-up visit will occur in May 2022.

**4 PRIMARY AND SECONDARY ENDPOINTS**

**4.1 Primary endpoint**

The primary endpoint is the first occurrence of all-cause death or BARC types 3-5 bleeding at 30 days after randomization. Time to event will be calculated using calendar days with date of randomization as day 0.

**4.2 Secondary endpoints**

Secondary endpoints listed below will be evaluated:

1. Net adverse clinical events (NACE, defined as a composite of all-cause death, recurrent myocardial infarction, ischemia-driven target vessel revascularization, stroke or BARC types 3-5 bleeding) at 30 days, 6 and 12 months after randomization;

2. The incidence of a composite of all-cause death or BARC types 2-5 bleeding at 30 days, 6 and 12 months after randomization;

3. Stent thrombosis (defined as definite or probable stent thrombosis according to Academic Research Consortium definition) at 30 days, 6 and 12 months after randomization;

4. Major adverse cardiac and cerebral events (MACCE, defined as a composite of all-cause death, recurrent myocardial infarction, ischemia-driven target vessel revascularization or stroke) at 30 days, 6 and 12 months after randomization;

5. BARC types 3-5 bleeding at 30 days, 6 and 12 months after randomization;

6. BARC types 2-5 bleeding at 30 days, 6 and 12 months after randomization;

7. Thrombocytopenia (defined as post-PCI platelet counts <150×10^9^/L for patients with baseline platelet count >150×10^9^/L) at 30 days after randomization;

8. The incidence of each individual event, including all-cause death, cardiac death, non-cardiac death, recurrent myocardial infarction, ischemia-driven target vessel revascularization, ischemia-driven target lesion revascularization and stroke at 30 days, 6 and 12 months after randomization.

**4.3 Safety endpoints**

Adverse events (AEs) of interest will be collected throughout the study period. All serious adverse events (SAEs) will be recorded from the time of informed consent throughout the study. Section 8 in the Protocol gives more detailed information on adverse event data collection.

**5 SAMPLE SIZE**

The study hypothesis is that bivalirudin with a prolonged high-dose infusion is superior to heparin alone for the 30-day primary endpoint, a composite of all-cause death or BARC types 3 to 5 bleeding after primary PCI in patients with STEMI.

H_0_: P_I_=P_C_

H_1_: P_I_≠P_C_, α=0.05 (two-sided)

P_I_ denotes the primary endpoint rate in the bivalirudin group, P_C_ is the primary endpoint rate in the control group.

Power calculations are based on a superiority comparison for the primary endpoint, a composite of all-cause death or BARC types 3-5 bleeding at 30 days after primary PCI in patients with STEMI. Assuming the incidence of the primary endpoint in the heparin group is 3.3%, and assuming 1% loss to follow-up the primary endpoint data at 30 days, 3000 evaluable patients in each group (6000 in total) are planned to be enrolled and randomly assigned in a 1:1 ratio, in order to provide 80% power to detect a 1.2% absolute risk reduction (35% relative risk reduction) in the bivalirudin group in comparison with the heparin group with a 2-sided type I error of 0.05.

**6 ANALYSIS SETS**

**6.1 Full analysis set (FAS)**

All patients who have been randomized to study treatment will be included irrespective of their protocol adherence and continued participation in the study. Patients will be analyzed according to their randomized study drug assignment (not to which treatment they actually received) irrespective of whether the event occurred before or following discontinuation of study drug. Patients who withdraw consent to participate in the study (or are lost to follow-up) will be included up to the date of their study termination except for vital status known through public records (for use in the analyses of deaths). All primary, secondary, and exploratory efficacy variables will be analyzed using the FAS. The FAS will be considered the primary analysis set for the primary and secondary variables and for the exploratory variables.

**6.2 Per protocol set (PPS)**

The PPS consists of all randomized subjects without any major deviations from the protocol. The following deviations in randomized subjects will lead to exclusion from the PP population. The primary analyses will be repeated in the PPS to support the primary results.

The following deviations will lead to exclusion from the PP population:

1. Not fulfilling randomization eligibility criteria at the time of randomization;

2. Not receiving the assigned treatment at the time of randomization.

**6.3 Safety analysis set**

The safety analysis set includes patients who received at least 1 dose of study drug and who have data observed at any time after randomization until the end of the study. Throughout the safety results sections, erroneously treated patients (patients randomized to one of the treatment groups but actually given the other treatment) will be accounted for in the actual treatment group. Patients with erroneous treatment would be analyzed according to that treatment only if they only received the erroneous treatment and none of the correct treatment.

All safety variables will be analyzed using the safety analysis set.

**7 SUBGROUPS**

The primary and secondary endpoints will be also analyzed in the following clinically relevant pre-specified subgroups.

- Age (<65 years, ≥65 years)

- Sex (male, female)

- BMI (<25kg/m^2^, ≥25kg/m^2^)

- Killip class (class I, II, III, IV)

- Diabetes mellitus (yes, no)

- Renal function (eGFR<60, ≥60 mL/min/1.73m^2^)

- Anemia (hemoglobin <12.0 g/dL in women and <13.0 g/dL in men; yes, no)

- Type of P2Y12 inhibitors (ticagrelor, clopidogrel)

- Any other anticoagulant treatment after PCI (yes, no)

- GRACE score (<140, ≥140)

- OPT-CAD score (<90, ≥90)

- CRUSADE score (<30, ≥30)

- First medical contact to PCI time (≤90 mins, > 90 mins)

- Access site (radial, femoral)

- Multi-vessel disease (yes, no)

- GPI during PCI (yes, no)

- Left main/Proximal LAD disease (yes, no)

- Circulation assist device (yes, no)

- No/slow-reflow (yes, no)

- ACT met standard after first bolus injection (yes, no, not performed)

All stratified analyses will be accompanied by a test for interaction between treatment effect and stratification factor.

**8 LOST TO FOLLOW-UP AND MISSING DATA**

In keeping with an intent-to-treat philosophy, the primary analyses are performed on all randomized subjects. We anticipate that the amount of missing data will be minimal in the present study as we are specifically asking all study sites to ensure that the data elements are complete. No imputation will be carried out for missing baseline data. Those subjects who withdraw, are lost to follow-up or have missing data will be included in the primary ITT analysis.

**9 STATISTICAL METHODS**

**9.1 General principles**

Statistical analyses will be performed using SAS version 9.3 or greater. Unless otherwise stated, all hypothesis tests will be performed using two-sided tests at the 5% significance level. Data will be summarized overall and by treatment group. Continuous variables will be summarized as the number of observations, number of missing values, mean, standard deviation, median, quartiles, and range. Categorical variables will be summarized as the number of observations, number of missing values, frequencies, and percentages. Baseline clinical, demographic, laboratory and procedural characteristics will be summarized by randomized treatment group using descriptive statistics. P-values will not be shown in the baseline table since any significant difference can be explained by the play of chance if the randomization was performed properly.

**9.2 Analysis for the primary endpoint**

The primary objective of BRIGHT-4 is to determine the impact of bivalirudin versus heparin on the endpoint of all-cause death or BARC types 3-5 bleeding that occurs between the time of randomization and 30 days thereafter. Event rates will be estimated at 30 days after randomization and Kaplan-Meier curves will be plotted from the time of randomization to the first occurrence of confirmed all-cause death or BARC types 3-5 bleeding by treatment group. Patients not experiencing a primary endpoint event during this time interval will be censored at last contact date (for subjects withdrawing consent of lost to follow-up) or 30 days after randomization, whichever comes first.

A hazard ratio (HR) and two-sided 95% CI for the primary endpoint will be generated using the Cox proportional hazards model that includes treatment group (bivalirudin versus heparin) as a covariate. The null hypothesis for this analysis is that the HR for the treatment group (H_0_) = 1. The alternative hypothesis is that the HR for the treatment group (H_1_) ≠ 1. A test of superiority at the two-sided 0.05 level will be performed and the null hypothesis will be rejected if the HR and 95% CI excludes one. This analysis will be performed in the ITT cohort and repeated in the PP cohort to support the primary results.

**9.3 Analysis for the secondary endpoints**

Analysis of the secondary endpoints will be based on the intention-to-treat principle by treatment group. Kaplan-Meier curves will be plotted by treatment group. Patients not experiencing an endpoint event during corresponding time interval will be censored at last contact date (for subjects withdrawing consent or lost to follow-up) or 30 days, whichever comes first.

A hazard ratio (HR) and two-sided 95% CI for the primary endpoint will be generated using the Cox proportional hazards model. The null hypothesis (H_0_) for this analysis is that the HR = 1. The two-sided alternative hypothesis (H_1_) is that the HR ≠ 1. A test of superiority at the two-sided 0.05 level will be performed using a p-value from a log-rank test.

- NACE, defined as a composite of all-cause death, recurrent MI, ischemia-driven target vessel revascularization, stroke or BARC types 3-5 bleeding at 30 days, 6 and 12 months after randomization;

- A composite of all-cause death or BARC types 2-5 bleeding at 30 days, 6 and 12 months after randomization;

- Definite or probable stent thrombosis according to ARC definition at 30 days, 6 and 12 months after randomization;

- MACCE, defined as a composite of all-cause death, recurrent MI, ischemia-driven target vessel revascularization or stroke at 30 days, 6 and 12 months after randomization;

- BARC types 3-5 bleeding at 30 days, 6 and 12 months after randomization;

- BARC types 2-5 bleeding at 30 days, 6 and 12 months after randomization;

- Thrombocytopenia (defined as post-PCI platelet counts <150×10^9^/L for patients with baseline platelet count >150×10^9^/L) at 30 days after randomization;

- The incidence of each individual event, including all-cause death, cardiac death, non-cardiac death, recurrent MI, ischemia-driven target vessel revascularization, ischemia-driven target lesion revascularization and stroke at 30 days, 6 and 12 months after randomization.

All analysis will be repeated in the PPS to support the primary results.

**9.4 Safety analysis**

All safety analyses will be based on the safety analysis set defined in Section 6.3.

All adverse events will be listed for the FAS population with randomized treatment, seriousness, severity, time of onset and resolution counted from randomization, relationship to heparin and/or bivalirudin, action taken, resolution, ICD-10 code and verbatim free text, and summarized by randomized treatment as number and percent of patients with event.

**9.5 Subgroup analysis**

The primary endpoint will be summarized in clinically relevant subgroups of the FAS as a whole and by treatment group. Subgroup analyses will be performed using a proportional hazards model with treatment, subgroup, and treatment-subgroup interactions, and will be presented with within-group hazard ratios with 95% CIs and the interaction p-value. Interaction p-values should be interpreted with caution because of the multiplicity of comparisons. Patients with missing data for the subgroup will be excluded from the analysis in question. The specific subgroups are shown in Section 7.

**9.6 Sensitivity analyses**

Sensitivity analyses of the primary and secondary endpoints will be performed in the PPS.

**10 TABLE LISTINGS**

**1. Enrolled Cohort**

1.1 Registration (inclusion and exclusion criteria)

1.1.1 Baseline demographics (age, sex, height, weight, et al.)

1.1.2 Baseline clinical and medical history (risk factors, pre-admission antiplatelet therapy, et al.)

1.1.3 Baseline vital signs and clinical presentation (heart rates, blood pressure, et al.)

1.1.4 Baseline laboratory assessment

1.1.5 Baseline medications pre-procedure

1.1.6 Baseline angiographic details

1.1.7 Baseline intervention details

1.1.8 Baseline medications in cath-lab

1.1.9 Adverse events during baseline hospitalization

1.2.1 Site reported AEs/SAEs

1.2.2 Adjudicated AEs/SAEs

1.2.3 Mortality assessment

1.2.4 Medications adherence (bivalirudin and heparin)

1.2.5 Eligibility for randomization evaluation

1.2.5.1 Reasons for randomization ineligibility

**2. Randomized ITT Cohort**

2.1 Registration (Inclusion and Exclusion criteria)

2.1.1 Baseline demographics (age, sex, height, weight, et al.)

2.1.2 Baseline clinical and medical history (risk factors, pre-admission antiplatelet therapy, et al.)

2.1.3 Baseline vital signs and clinical presentation (heart rates, blood pressure, et al.)

2.1.4 Baseline laboratory assessment

2.1.5 Baseline medications pre-procedure

2.1.6 Baseline angiographic details

2.1.7 Baseline intervention details

2.1.8 Baseline medications in cath-lab

2.1.9 Adverse events during baseline hospitalization

2.2.1 Site reported clinical events

2.2.1.1 30 days binary frequency and Kaplan-Meier estimates

2.2.1.2 6 months binary frequency and Kaplan-Meier estimates

2.2.1.3 12 months binary frequency and Kaplan-Meier estimates

2.2.2 Adjudicated clinical events

2.2.2.1 30 days binary frequency and Kaplan-Meier estimates

2.2.2.2 6 months binary frequency and Kaplan-Meier estimates

2.2.2.3 12 months binary frequency and Kaplan-Meier estimates

2.2.3 Medications adherence (bivalirudin and heparin)

2.2.4 Subject disposition (lost or consent withdrawn)

**11 REFERENCES**

1. Steg PG, van 't Hof A, Hamm CW, Clemmensen P, Lapostolle F, Coste P, et al. Bivalirudin started during emergency transport for primary PCI. N Engl J Med. 2013;369(23):2207-17.

2. Gregg W. Stone, Bernhard Witzenbichler, Giulio Guagliumi, Jan Z. Peruga, Bruce R. Brodie, Dariusz Dudek, et al. Bivalirudin during Primary PCI in Acute Myocardial Infarction. N Engl J Med. 2008(358):2218-30.

3. Shahzad A, Kemp I, Mars C, Wilson K, Roome C, Cooper R, et al. Unfractionated heparin versus bivalirudin in primary percutaneous coronary intervention (HEAT-PPCI): an open-label, single centre, randomised controlled trial. The Lancet. 2014;384(9957):1849-58.

4. Cavender MA, Sabatine MS. Bivalirudin versus heparin in patients planned for percutaneous coronary intervention: a meta-analysis of randomised controlled trials. Lancet. 2014;384(9943):599-606.

5. Stone GW, Mehran R, Goldstein P, Witzenbichler B, Van't Hof A, Guagliumi G, et al. Bivalirudin versus heparin with or without glycoprotein IIb/IIIa inhibitors in patients with STEMI undergoing primary percutaneous coronary intervention: pooled patient-level analysis from the HORIZONS-AMI and EUROMAX trials. J Am Coll Cardiol. 2015;65(1):27-38.

6. Han Y, Guo J, Zheng Y, Zang H, Su X, Wang Y, et al. Bivalirudin vs heparin with or without tirofiban during primary percutaneous coronary intervention in acute myocardial infarction: the BRIGHT randomized clinical trial. JAMA. 2015;313(13):1336-46.

7. Ibanez B, James S, Agewall S, Antunes MJ, Bucciarelli-Ducci C, Bueno H, et al. 2017 ESC Guidelines for the management of acute myocardial infarction in patients presenting with ST-segment elevation: The Task Force for the management of acute myocardial infarction in patients presenting with ST-segment elevation of the European Society of Cardiology (ESC). Eur Heart J. 2018;39(2):119-77.
