# Supplementary material for: Analysis of inter-hospital transfer on clinical outcomes after primary percutaneous coronary intervention for ST-segment elevation myocardial infarction: A secondary analysis of the BRIGHT-4 trial
Source: PLoS Med. 2025 Jul 23;22(7):e1004679. doi: 10.1371/journal.pmed.1004679 (PMC12313069; doi:10.1371/journal.pmed.1004679)
Supplement: Attachment — Submitted filename: Response to reviewers.docx [file pmed.1004679.s015.docx]

**Submission ID: PMEDICINE-D-24-04173R1**

**Impact of Inter-hospital Transfer on Clinical Outcomes After Primary PCI in STEMI: The BRIGHT-4 Trial**

Dear Editors and Reviewers:

Thanks a lot for your careful reading and valuable suggestions concerning our manuscript entitled "Impact of Inter-hospital Transfer on Clinical Outcomes After Primary PCI in STEMI: The BRIGHT-4 Trial" (PMEDICINE-D-24-04173R1). Those comments are very helpful for revising and improving our paper, as well as are of the important guiding significance to our researches.

According to your comments, we have checked and revised our manuscript. The changes we made will not influence the content and framework of the manuscript. In the following sections, we provide detailed, point-by-point responses to each comment. Hope the corrections can meet the comments with approval.

We earnestly appreciate for your warm work, thank you very much again for your comments and suggestions. We greatly value your support and are happy to address any further questions or suggestions you may have.

Sincerely yours,

Yaling Han, MD, FACC, FESC, State Key Laboratory of Frigid Zone Cardiovascular Disease, Department of Cardiology, General Hospital of Northern Theatre Command, Shenyang 110016, China. E-mail: [hanyaling@163.net](mailto:hanyaling@163.net)

The corrections and answers in response to your comments are listed as follows:

**Answers to Reviewers**

**Reviewer #1:**

The BRIGHT 4 trial was a randomised trial comparing bivalirudin with heparin in more than 6000 patients with STEMI. Approximately 36% of patients required transfer from the hospital of admission to a tertiary PCI-capable hospital. Although the time from symptom onset to first medical contact was significantly shorter in the transfer group, transfer caused a delay of about 2.5 hours from symptom onset to final hospital arrival compared with those who did not require transfer. Despite this delay, the outcomes of patients who required transfer were not significantly different from those who did not. These are interesting data, although they contradict the common belief that "time is muscle". Apparently, the baseline characteristics of the patients do not suggest that the transfer group necessarily has a lower risk that compensates for the presumed disadvantage of delayed treatment. However, more information is needed to clarify the possible reasons for this finding:

**Comment 1:** Were the tertiary hospitals the same for both transferred and non-transferred patients? Were there differences between these hospitals in terms of primary PCI volume and guideline-based management strategy? It is noteworthy that ticagrelor was used significantly more often in the hospitals receiving non-transferred patients.

**Response:** Thank you for this important question.

1) All participating tertiary hospitals received both directly admitted patients and those transferred from other facilities. The study design specifically selected the largest tertiary centers in each region, based on their high annual PCI volumes. As a result, the same or comparable tertiary hospitals managed both transferred and non-transferred patients across the study population.
2) Although there may have been some variation in annual PCI volume among the participating tertiary hospitals, all centers demonstrated consistent adherence to guideline-based management strategies for ST-elevation myocardial infarction (STEMI). Furthermore, our study protocol explicitly required compliance with current clinical guidelines for medication use, with the exception of the randomized assignment of either bivalirudin or heparin. In response, we compared guideline-recommended medication between directly admitted and transferred patients, as presented in S1 Table below.

**S1 Table. Medication use**

|  | **Inter-hospital transfer (N=2121)** | **Direct admission**  **(N=3817)** | ***P* Value** |
| --- | --- | --- | --- |
| Chronic oral anticoagulant | 45 (2.1%) | 98 (2.6%) | 0.28 |
| Warfarin | 13 (0.6%) | 31 (0.8%) | 0.39 |
| Direct-acting oral anticoagulant | 32 (1.5%) | 67 (1.8%) | 0.48 |
| Beta-blocker | 1611 (76.0%) | 2901 (76.0%) | 0.97 |
| Calcium channel blocker | 167 (7.9%) | 281 (7.4%) | 0.47 |
| ACEI/ARB/ARNI | 1233 (58.1%) | 2180 (57.1%) | 0.45 |
| Diuretic | 1269 (59.8%) | 2220 (58.2%) | 0.21 |
| Any lipid-lowering agent | 2000 (94.3%) | 3637 (95.3%) | 0.10 |
| Statin | 1911 (90.1%) | 3460 (90.6%) | 0.49 |
| Other lipid-lowering agent | 103 (4.9%) | 188 (4.9%) | 0.91 |

Data are shown as n (%). ACEI, angiotensin converting enzyme inhibitor. ARB, angiotensin receptor blocker. ARNI, angiotensin receptor-neprilysin inhibitor.

3) Ticagrelor was more frequently prescribed in patients directly admitted for primary PCI. This preference may be due to a worse baseline clinical profile in the direct admission group, but it is more likely explained by the unavailability of ticagrelor in some referring hospitals. Consequently, patients may have received a loading dose of clopidogrel upon their first medical contact (FMC) in primary-level settings. To address this issue, we have added the following statement to the Discussion section (Page 14, Lines 330-333): “Additionally, it is also worth noting that ticagrelor, providing faster and greater P2Y12 receptor inhibition than clopidogrel, was more frequently chosen in patients directly admitted for primary PCI, which might be due to a worse baseline clinical profile in the direct admission group or lack of ticagrelor in some referring hospitals.”

**Comment 2:** It has been hypothesized that the disadvantage of treatment delay is almost eliminated when the interval from symptom onset is greater than 4-5 hours (JAMA, 23 February 2005-Vol 293, No 8). The authors show the percentage of patients with an interval >12 hours. However, the information on treatment interval should be more detailed: e.g. proportion of patients between 3 and 6 hours, between 6 and 12 hours. I am aware of the problems attributed to analyses of subgroups of subgroups, but the exercise of looking at outcomes according to these subgroups might be informative.

**Response:** Thank you for your insightful comment. We fully agree with your suggestion and have stratified patients according to symptom onset-to-wire time into the following subgroups: <3 hours, 3-6 hours, 6-12 hours, and ≥12 hours. We then used multivariable Cox models to analyze primary outcomes across these subgroups, as presented in S10 Table below. To address this point, we have added the following statement to the Results section (Page 11, Lines 270-275): “According to S10 Table, we further stratified the symptom onset-to-wire time and found that the incidence of the primary outcome increased with longer symptom onset-to-wire intervals, regardless of whether the analysis was conducted in the overall population or in the directly admitted and transferred subgroups. Although the ≥ 12 hours group showed a slight decrease in the primary outcome, this finding should be interpreted with caution given the relatively small number of patients in this category.”

**S10 Table. Multivariable-adjusted HRs (95% CI) of 30-day primary outcome according to symptom onset-to-wire time**

|  | **Primary outcome (%)** | **Adjusted HR (95%CI)** | ***P* value** |
| --- | --- | --- | --- |
| **All population** |  |  |  |
| < 3 hours (N=1174) | 22 (1.9%) | Reference | - |
| 3-6 hours (N=1679) | 42 (2.5%) | 1.31 (0.77-2.23) | 0.32 |
| 6-12 hours (N=1015) | 52 (5.1%) | 2.15 (1.27-3.64) | 0.004 |
| ≥ 12 hours (N=613) | 23 (3.8%) | 1.37 (0.74-2.52) | 0.31 |
| **Direct admission** |  |  |  |
| < 3 hours (N=992) | 18 (1.8%) | Reference | - |
| 3-6 hours (N=1079) | 25 (2.3%) | 1.29 (0.69-2.41) | 0.43 |
| 6-12 hours (N=539) | 30 (5.6%) | 2.55 (1.38-4.69) | 0.003 |
| ≥ 12 hours (N=304) | 13 (4.3%) | 1.63 (0.78-3.43) | 0.20 |
| **Inter-hospital transfer** |  |  |  |
| < 3 hours (N=182) | 4 (2.2%) | Reference | - |
| 3-6 hours (N=600) | 17 (2.8%) | 1.19 (0.37-3.87) | 0.77 |
| 6-12 hours (N=476) | 22 (4.6%) | 1.80 (0.54-6.00) | 0.34 |
| ≥ 12 hours (N=309) | 10 (3.2%) | 1.19 (0.33-4.31) | 0.79 |

Event rates are number of events. Primary outcome, all-cause death or BARC types 3-5 bleeding.

**Comment 3:** The implications for the current guideline recommendations of a pharmaco-invasive approach for expected delays of 2 hours or more need to be discussed.

**Response:** Thank you for your valuable comment. As you correctly point out, current guidelines recommend a pharmaco-invasive strategy for STEMI patients when FMC-to-wire time is expected to exceed 120 minutes. However, in our study, patients who received thrombolytic therapy prior to PCI were specifically excluded, which limits our ability to evaluate the pharmaco-invasive strategy. We then have added the following statement to the Discussion section (Page 14, Lines 333-340): “These limitations at referring hospitals emphasize the necessity for optimized reperfusion strategies in scenarios when delays are inevitable. Current guidelines advocate the pharmaco-invasive strategy when FMC-to-wire time exceeds 120 minutes [2]. However, our study excluded patients who received thrombolytic therapy prior to PCI, which limited our ability to evaluate the pharmaco-invasive strategy. This critical evidence gap highlights the urgent need for future studies comparing clinical outcomes between STEMI patients undergoing direct primary PCI and those receiving pharmaco-invasive therapy following inter-hospital transfer.”

2. Ibanez B, James S, Agewall S, Antunes MJ, Bucciarelli-Ducci C, Bueno H, et al. 2017 ESC Guidelines for the management of acute myocardial infarction in patients presenting with ST-segment elevation: The Task Force for the management of acute myocardial infarction in patients presenting with ST-segment elevation of the European Society of Cardiology (ESC). Eur Heart J. 2018;39(2):119-77.

**Reviewer #2:**

As the statistical reviewer I will focus on methods and reporting. Overall, this is a well conducted post-hoc analysis and also well reported. I have some points to raise.

**Comment 1:** There is no mention of missing data and how that was handled. At the end of the statistical analysis section this needs to be discussed. If all data were complete, please state so. if not, why weren't multiple imputation approaches considered?

**Response:** Thank you for raising this important methodological consideration. In our study, we implemented rigorous data quality control measures to minimize missing data. Specifically, all study sites were prospectively required to verify complete data submission. Additionally, follow-up data at 30 days was available in 99.7% (5998/6016) of randomized patients. Given this level of data completeness, no imputation was used to infer missing values. Thus, we have added the following statement to the Method section (Page 8, Lines 202-203): “No imputation was used to infer missing values."

**Comment 2:** This is a multi-centre study. why weren't random effects (shared frailty) Cox models considered?

**Response:** Thank you for this valuable suggestion. We have performed supplementary analyses using Cox frailty models to evaluate center-specific effects, as presented in S4 Table. In response, we added the following statement to the Method section (Page 9, Lines 213-215): “Given the multi-center design of the study, Cox frailty models were further applied to estimate hazard ratios (HRs) for the primary outcome, incorporating center as a random intercept to account for clustering effects." and to the Results section (Page 10-11, Lines 256-258): “As shown in Table S4, the results from both standard Cox models and Cox frailty models for the primary outcome were consistent, which suggested that the observed associations were not significantly influenced by inter-center variability.”

**S4 Table. Comparison of HRs (95%CI) for the primary outcome: Cox model vs. Cox frailty model**

|  | **Unadjusted HR (95%CI)** | ***P* value** | **Adjusted HR (95%CI)** | ***P* value** |
| --- | --- | --- | --- | --- |
| **Inter-hospital transfer vs. Direct admission** |  |  |  |  |
| Cox models | 1.21 (0.92-1.59) | 0.17 | 0.99 (0.73-1.33) | 0.94 |
| Cox frailty models | 1.20 (0.91-1.59) | 0.21 | 0.92 (0.67-1.25) | 0.59 |
| **Inter-hospital transfer (Bivalirudin vs. Heparin)** |  |  |  |  |
| Cox models | 0.72 (0.47-1.10) | 0.13 | 0.66 (0.42-1.05) | 0.08 |
| Cox frailty models | 0.72 (0.47-1.10) | 0.13 | 0.65 (0.41-1.04) | 0.07 |
| **Direct admission**  **(Bivalirudin vs. Heparin)** |  |  |  |  |
| Cox models | 0.67 (0.47-0.95) | 0.02 | 0.62 (0.43-0.89) | 0.01 |
| Cox frailty models | 0.68 (0.48-0.97) | 0.03 | 0.63 (0.44-0.92) | 0.02 |

Event rates are number of events (Kaplan-Meier estimated percentages). HR, hazard ratio.

**Comment 3:** a sensitivity analysis with propensity score matching (between patients with and without inter-hospital transfer) would go a long way in offering reassurance for the findings.

**Response:** Thank you for your suggestion. We have performed the requested propensity score matching analysis to further validate our findings, as shown in S5 Table. In response, we added the following statement to the Method section (Page 9, Lines 216-219): “To confirm the robustness of our findings, propensity score matching (PSM) was conducted between patients with and without inter-hospital transfer using a 1:1 nearest-neighbor matching algorithm with a caliper of 0.2 standard deviations. The matched variables were identical to those included as covariates in multivariable Cox models." and to the Results section (Page 11, Lines 258-260): “Furthermore, we conducted a PSM analysis, which identified 1896 well-balanced pairs. The results of this sensitivity analysis (see S5 Table) were in line with our primary findings.”

**S5 Table. Clinical outcomes at 30 days after propensity score matching**

|  | **Inter-hospital transfer (N=1896)** | **Direct admission  (N=1896)** | **HR (95%CI)** | ***P* Value** |
| --- | --- | --- | --- | --- |
| Primary outcome: All-cause death or BARC types 3-5 bleeding | 72 (3.8%) | 70 (3.7%) | 1.03 (0.74-1.43) | 0.87 |
| Death from any cause | 64 (3.4%) | 65 (3.4%) | 0.98 (0.70-1.39) | 0.92 |
| From cardiovascular causes | 61 (3.2%) | 62 (3.3%) | 0.98 (0.69-1.40) | 0.93 |
| BARC types 3-5 bleeding | 13 (0.7%) | 9 (0.5%) | 1.45 (0.62-3.38) | 0.40 |
| Reinfarction | 16 (0.8%) | 13 (0.7%) | 1.23 (0.59-2.56) | 0.58 |
| Ischemia-driven TVR | 8 (0.4%) | 7 (0.4%) | 1.14 (0.41-3.15) | 0.80 |
| Stroke | 10 (0.5%) | 8 (0.4%) | 1.25 (0.49-3.17) | 0.64 |
| Stent thrombosis | 16 (0.8%) | 13 (0.7%) | 1.23 (0.59-2.56) | 0.58 |
| Acute (<24 hours) | 7 (0.4%) | 4 (0.2%) | 1.75 (0.51-5.98) | 0.37 |
| Subacute (1-30 days) | 9 (0.5%) | 9 (0.5%) | 1.00 (0.40-2.52) | 1.00 |
| MACCE* | 87 (4.6%) | 87 (4.6%) | 1.00 (0.74-1.35) | >0.999 |
| BARC types 2-5 bleeding | 36 (1.9%) | 48 (2.5%) | 0.75 (0.48-1.15) | 0.19 |
| All-cause death or BARC types 2-5 bleeding | 94 (5.0%) | 107 (5.6%) | 0.87 (0.66-1.15) | 0.34 |
| Acquired thrombocytopenia† | 87 (4.6%) | 71 (3.7%) | 1.23 (0.90-1.68) | 0.19 |
| NACE‡ | 93 (4.9%) | 91 (4.8%) | 1.02 (0.77-1.36) | 0.88 |

Event rates are number of events (Kaplan-Meier estimated percentages). MACCE, Major adverse cardiac or cerebral events. NACE, Net adverse clinical events. *MACCE includes all-cause death, myocardial infarction, ischemia-driven target vessel revascularization, or stroke. †Defined as nadir platelet count of <150×10^9^ cells/L after the index procedure in patients in whom the baseline platelet count was ≥150×10^9^ cells/L. ‡ NACE includes MACCE or BARC types 3–5 bleeding.

**Comment 4:** was the proportional hazards assumption assessed and how? if it does not stand, consider time-varying covariates.

**Response:** Thank you for this important suggestion. According to the statistical analysis plan of this study, the Cox proportional hazards assumption was tested using log(-log) plots. To address this issue, we added the following statement to the Method section (Page 8-9, Lines 211-213): “The Cox proportional hazards assumption for the primary outcome was confirmed graphically using log(-log) plots.”

**Reviewer #3:**

The statistical methods used in this manuscript are generally appropriate for the study objectives and are well-executed. A few main points to consider:

**Comment 1:** Please clarify whether Cox proportional hazards assumptions were tested.

**Response:** Thank you for this important suggestion. According to the statistical analysis plan of this study, the Cox proportional hazards assumption was tested using log(-log) plots. To address this issue, we added the following statement to the Method section (Page 8-9, Lines 211-213): “The Cox proportional hazards assumption for the primary outcome was confirmed graphically using log(-log) plots.”

**Comment 2:** Specify whether covariates were prespecified or selected based on univariate associations.

**Response:** Thank you for this important methodological question. According to the statistical analysis plan of this study, all covariates included in the Cox proportional hazards models were prospectively specified based on both clinical relevance and statistical considerations. Specifically, we included age, sex, baseline clinical characteristics, medications and so on, which have been shown to be significant predictors of outcomes in previous studies.

**Comment 3:** The handling of missing data is not described (e.g., complete case analysis, imputation) and should be reported.

**Response:** Thank you for raising this important methodological consideration. In our study, we implemented rigorous data quality control measures to minimize missing data. Specifically, all study sites were prospectively required to verify complete data submission. Additionally, follow-up data at 30 days was available in 99.7% (5998/6016) of randomized patients. Given this level of data completeness, no imputation was used to infer missing values. Thus, we have added the following statement to the Method section (Page 8, Lines 202-203): “No imputation was used to infer missing values."

**Comment 4:** Multiple secondary endpoints and subgroup analyses are tested without adjustment; considering adjustment and acknowledge increased type I error risk.

**Response:** Thank you for your insightful observation concerning the methodological approach. No adjustment was made for multiple comparisons, which may have increased the risk of type I error. To address this issue, we have revised the first point in the Limitations section (Page 16-17, Lines 386-394) as follows:” First, as a *post hoc* analysis, randomization was not stratified by transfer status, and no adjustment was made for multiple comparisons, which may have increased the risk of type I error. Although multivariable analysis was used to adjust for differences in baseline characteristics, medications and procedures (most of which were modest) between the inter-hospital transfer and direct admission groups, selection bias and unmeasured confounders cannot be excluded. Therefore, these results should be interpreted with caution in the context of an underpowered subgroup analysis. Accordingly, our results should be considered hypothesis-generating rather than conclusive inference and warrant dedicated, prospective confirmation.”

**Comment 5:** Consider providing sensitivity analyses (e.g., excluding very late presenters, >24h symptom onset-to-wire time) to confirm robustness.

**Response:** Thank you for this constructive suggestion. In response, we conducted sensitivity analyses by excluding very late presenters (defined as symptom onset-to-wire time >24 hours, N=175) to confirm the robustness of our primary findings, as shown in S6 and S7 Table. In response, we added the following statement to the Method section (Page 9, Lines 219-221): “In addition, we excluded patients with symptom onset-to-wire time exceeding 24 hours and employed multivariable Cox models to test the consistency of our hypothesis.” and to the Results section (Page 11, Lines 260-263): “Lastly, we performed an additional analysis excluding patients with symptom onset-to-wire time exceeding 24 hours, and applied multivariable Cox models. The results (see S6 and S7 Tables) remained consistent with those from the primary analysis."

**S6 Table. Clinical outcomes at 30 days in patients with symptom onset-to-wire time ≤ 24 hours**

|  | **Inter-hospital transfer (N=1468)** | **Direct admission  (N=2838)** | **Unadjusted HR (95%CI)** | ***P* Value** | **Adjusted HR (95%CI)** | ***P* Value** |
| --- | --- | --- | --- | --- | --- | --- |
| Primary outcome: All-cause death or BARC types 3-5 bleeding | 53 (3.6%) | 82 (2.9%) | 1.25 (0.89-1.77) | 0.20 | 0.99 (0.68-1.46) | 0.97 |
| Death from any cause | 46 (3.1%) | 78 (2.7%) | 1.14 (0.79-1.64) | 0.48 | 0.91 (0.60-1.36) | 0.63 |
| From cardiovascular causes | 46 (3.1%) | 75 (2.6%) | 1.19 (0.82-1.71) | 0.36 | 0.95 (0.63-1.43) | 0.80 |
| BARC types 3-5 bleeding | 9 (0.6%) | 9 (0.3%) | 1.93 (0.77-4.87) | 0.16 | 1.51 (0.56-4.02) | 0.41 |
| Reinfarction | 10 (0.7%) | 22 (0.8%) | 0.88 (0.42-1.86) | 0.73 | 0.95 (0.44-2.08) | 0.91 |
| Ischemia-driven TVR | 5 (0.3%) | 15 (0.5%) | 0.64 (0.23-1.77) | 0.39 | 0.63 (0.22-1.79) | 0.39 |
| Stroke | 9 (0.6%) | 18 (0.6%) | 0.97 (0.43-2.15) | 0.93 | 1.11 (0.49-2.53) | 0.80 |
| Stent thrombosis | 11 (0.7%) | 23 (0.8%) | 0.93 (0.45-1.90) | 0.83 | 0.96 (0.46-2.03) | 0.92 |
| Acute (<24 hours) | 7 (0.5%) | 8 (0.3%) | 1.69 (0.61-4.66) | 0.31 | 1.61 (0.56-4.67) | 0.38 |
| Subacute (1-30 days) | 4 (0.3%) | 15 (0.5%) | 0.52 (0.17-1.55) | 0.24 | 0.57 (0.18-1.77) | 0.33 |
| MACCE* | 61 (4.2%) | 119 (4.2%) | 0.99 (0.73-1.35) | 0.95 | 0.88 (0.63-1.23) | 0.47 |
| BARC types 2-5 bleeding | 30 (2.0%) | 79 (2.8%) | 0.73 (0.48-1.11) | 0.14 | 0.74 (0.48-1.14) | 0.17 |
| All-cause death or BARC types 2-5 bleeding | 74 (5.0%) | 149 (5.3%) | 0.96 (0.72-1.27) | 0.76 | 0.87 (0.64-1.17) | 0.35 |
| Acquired thrombocytopenia† | 71 (4.9%) | 89 (3.1%) | 1.56 (1.14-2.13) | 0.005 | 1.39 (1.00-1.93) | 0.05 |
| NACE‡ | 66 (4.5%) | 122 (4.3%) | 1.05 (0.78-1.41) | 0.77 | 0.92 (0.67-1.27) | 0.61 |

Event rates are number of events (Kaplan-Meier estimated percentages). Abbreviations and footnotes as in S5 Table.

**S7 Table. Clinical outcomes at 30 days according to randomization to bivalirudin vs. heparin in patients with symptom onset-to-wire time ≤ 24 hours**

|  | **Direct admission (N=2838)** | | | | **Inter-hospital transfer (N=1468)** | | | |
| --- | --- | --- | --- | --- | --- | --- | --- | --- |
|  | **Bivalirudin  (N=1405)** | **Heparin (N=1433)** | **Adjusted HR (95%CI)** | ***P* value** | **Bivalirudin  (N=739)** | **Heparin  (N=729)** | **Adjusted HR (95%CI)** | ***P* value** |
| Primary outcome: All-cause death or BARC types 3-5 bleeding | 27 (1.9%) | 55 (3.8%) | 0.47 (0.29-0.76) | 0.002 | 25 (3.4%) | 28 (3.8%) | 0.87 (0.48-1.57) | 0.65 |
| Death from any cause | 26 (1.9%) | 52 (3.6%) | 0.48 (0.30-0.79) | 0.004 | 24 (3.2%) | 22 (3.0%) | 1.15 (0.60-2.19) | 0.68 |
| From cardiovascular causes | 26 (1.9%) | 49 (3.4%) | 0.52 (0.31-0.85) | 0.009 | 24 (3.2%) | 22 (3.0%) | 1.15 (0.60-2.19) | 0.68 |
| BARC types 3-5 bleeding | 2 (0.1%) | 7 (0.5%) | 0.21 (0.03-1.29) | 0.09 | 1 (0.1%) | 8 (1.1%) | 0.03 (0.00-0.48) | 0.01 |
| Reinfarction | 9 (0.6%) | 13 (0.9%) | 0.70 (0.30-1.65) | 0.42 | 4 (0.5%) | 6 (0.8%) | 0.83 (0.20-3.54) | 0.80 |
| Ischemia-driven TVR | 5 (0.4%) | 10 (0.7%) | 0.49 (0.17-1.45) | 0.20 | 1 (0.1%) | 4 (0.5%) | 0.42 (0.01-24.23) | 0.68 |
| Stroke | 9 (0.6%) | 9 (0.6%) | 1.09 (0.43-2.77) | 0.86 | 5 (0.7%) | 4 (0.5%) | 1.29 (0.31-5.30) | 0.73 |
| Stent thrombosis | 5 (0.4%) | 18 (1.3%) | 0.27 (0.10-0.74) | 0.01 | 4 (0.5%) | 7 (1.0%) | 0.51 (0.13-2.02) | 0.34 |
| Acute (<24 hours) | 2 (0.1%) | 6 (0.4%) | 0.31 (0.06-1.57) | 0.16 | 2 (0.3%) | 5 (0.7%) | 0.23 (0.03-1.92) | 0.18 |
| Subacute (1-30 days) | 3 (0.2%) | 12 (0.8%) | 0.25 (0.07-0.90) | 0.03 | 2 (0.3%) | 2 (0.3%) | 0.88 (0.10-7.58) | 0.91 |
| MACCE* | 47 (3.3%) | 72 (5.0%) | 0.66 (0.45-0.96) | 0.03 | 30 (4.1%) | 31 (4.3%) | 1.08 (0.63-1.86) | 0.77 |
| BARC types 2-5 bleeding | 41 (2.9%) | 38 (2.7%) | 1.13 (0.72-1.77) | 0.59 | 11 (1.5%) | 19 (2.6%) | 0.51 (0.23-1.12) | 0.09 |
| All-cause death or BARC types 2-5 bleeding | 64 (4.6%) | 85 (5.9%) | 0.77 (0.55-1.07) | 0.13 | 35 (4.7%) | 39 (5.3%) | 0.88 (0.54-1.44) | 0.62 |
| Acquired thrombocytopenia† | 43 (3.1%) | 46 (3.2%) | 0.92 (0.60-1.41) | 0.70 | 30 (4.1%) | 41 (5.6%) | 0.74 (0.46-1.21) | 0.23 |
| NACE‡ | 48 (3.4%) | 74 (5.2%) | 0.65 (0.45-0.95) | 0.02 | 30 (4.1%) | 36 (4.9%) | 0.90 (0.53-1.51) | 0.68 |

Event rates are number of events (Kaplan-Meier estimated percentages). Abbreviations and footnotes as in S5 Table.

**Comment 6:** Terms like "symptom onset-to-wire time" and "symptom onset-to-reperfusion time" are used interchangeably; recommend standardizing.

**Response:** Thank you for this correction. We have standardized the terminology throughout the revised manuscript to use "symptom onset-to-wire time".

**Comment 7:** Non-significant hazard ratios may still have clinically important ranges (e.g., HR 0.66 [0.42-1.05]); better discussion of possible clinical relevance is advised.

**Response:** Thank you for your valuable comment. Although some HRs did not reach statistical significance in inter-hospital transfer group in Table 4, it suggests a potential reduction in risk that could be clinically important. We have discussed that the relative hazards for all primary and secondary ischemic and bleeding outcomes with these anticoagulation regimens were consistent in the inter-hospital transfer and direct admission groups. Thus, STEMI patients undergoing primary PCI who are anticoagulated with bivalirudin plus a post-PCI high-dose infusion for 2-4 hours have lower rates of all-cause mortality, major bleeding, stent thrombosis and NACE compared with heparin monotherapy, regardless of whether patients were transferred or not. We then added the following statement to the Discussion section (Page 15, Lines 352-353): “Future studies with larger sample sizes or longer follow-up periods may help clarify whether this trend translates into a significant benefit."

**Comment 8:** Direct references to Supplementary Tables should be embedded earlier in the main Results text for easier navigation.

**Response:** Thank you for your advice. We have embedded direct references to the supplementary tables earlier in the main Results section to improve clarity and facilitate navigation for readers. Specifically, we have revised the following statement in the Results section as follows: (Page 10, Line 241)” As presented in S2 and S3 Tables, baseline characteristics, procedural data, and medications....” and (Page 11, Line 264) “As shown in S8 and S9 Tables, Outcomes significant differences emerged in 30-day outcomes....”

**Answers to Editors**

**Comment 1:** We also ask you to further discuss the potential relevance of the findings for clinical implementation or healthcare policy.

**Response:** Thank you for your valuable comment. We added the following statement to the Discussion section (Page 16, Lines 377-385): “This study highlighted the value of a time-based stratified management strategy that prioritized total ischemic time over the specific mode of transfer. It underscored the essential role of timely reperfusion in optimizing outcomes for both directly admitted and transferred STEMI patients. The results supported the hypothesis that bivalirudin might serve as a preferable anticoagulant option during primary PCI, offering enhanced safety and survival benefits compared to heparin monotherapy, regardless of whether patients were transferred or not. Nonetheless, further research is warranted to evaluate the long-term prognostic implications of inter-hospital transfer and to assess the sustained benefits of bivalirudin therapy over time.”

**Comment 2:** We ask every co-author listed on the manuscript to fill in a contributing author statement, making sure to declare all competing interests. If any of the co-authors have not filled in the statement, we will remind them to do so when the paper is revised. If all statements are not completed in a timely fashion this could hold up the re-review process. If new competing interests are declared later in the revision process, this may also hold up the submission. Should there be a problem getting one of your co-authors to fill in a statement we will be in contact. Please do not add or remove authors without first discussing this with the handling editor. You can see our competing interests policy here: http://journals.plos.org/plosmedicine/s/competing-interests.

**Response:** Thank you for this advice. We have thoroughly read [PLOS Competing Interests Policy](http://journals.plos.org/plosmedicine/s/competing-interests) and are in compliance with it. We have revised “Competing Interests” statement (Page 20, Lines 460-461) as follows: ”I have read the journal's policy and the authors of this manuscript have the following competing interests: Dr. Stone has received speaker honoraria from Pulnovo, Medtronic, Amgen, Boehringer Ingelheim, Abiomed; has served as a consultant to CorFlow, Cardiomech, Robocath, Daiichi Sankyo, Ablative Solutions, Vectorious, Miracor, Apollo Therapeutics, Elucid Bio, Abbott, Cardiac Success, Occlutech, Millennia Biopharma, Remote Cardiac Enablement, Valfix, Zoll, HeartFlow, Shockwave, Impulse Dynamics, Adona Medical, Oxitope, HighLife, Elixir, Aria; and has equity/options from Cardiac Success, Ancora, Cagent, Applied Therapeutics, Biostar family of funds, SpectraWave, Orchestra Biomed, Aria, Valfix, Xenter. Dr. Stone’s employer, Mount Sinai Hospital, receives research grants from Shockwave, Biosense-Webster, Abbott, Abiomed, Bioventrix, Cardiovascular Systems Inc, Phillips, Vascular Dynamics, Pulnovo, V-wave and PCORI (via Weill Cornell Medical Center). The other authors have no disclosures.”

**Comment 3:** Please upload any figures associated with your paper as individual TIF or EPS files with 300dpi resolution at resubmission; please read our figure guidelines for more information on our requirements: http://journals.plos.org/plosmedicine/s/figures. While revising your submission, please upload your figure files to the PACE digital diagnostic tool, https://pacev2.apexcovantage.com/. PACE helps ensure that figures meet PLOS requirements. To use PACE, you must first register as a user. Then, login and navigate to the UPLOAD tab, where you will find detailed instructions on how to use the tool. If you encounter any issues or have any questions when using PACE, please email us at PLOSMedicine@plos.org.

**Response:** Thank you for this advice. We confirm that all figures in our manuscript have been uploaded as individual TIF files with 300dpi resolution at resubmission, in accordance with the PLOS Medicine figure guidelines. We have used the PACE digital diagnostic tool for compliance verification.

**Comment 4:** Please ensure that the paper adheres to the PLOS Data Availability Policy (see http://journals.plos.org/plosmedicine/s/data-availability), which requires that all data underlying the study's findings be provided in a repository or as Supporting Information. For data residing with a third party, authors are required to provide instructions with contact information (web or email address) for obtaining the data. Please note that a study author cannot be the contact person for the data. PLOS journals do not allow statements supported by "data not shown" or "unpublished results." For such statements, authors must provide supporting data or cite public sources that include it.

**Response:** Thank you for this advice. We have thoroughly read PLOS Data Availability Policy and are in compliance with it. We have added “Data Availability” statement (Page 21, Lines 490-492) as follows:” The authors are willing to share de-identified individual data with researchers who provide a methodologically sound proposal. Interested parties should contact the Clinical Research Data Center of the General Hospital of Northern Theatre Command via [Lcyjsjzx@163.com.”](mailto:Lcyjsjzx@163.com.) All data supporting the Results and Discussion sections of this manuscript are provided in the main text or the Supporting Information section.

**Comment 5:** We expect all researchers with submissions to PLOS in which author-generated code underpins the findings in the manuscript to make all author-generated code available without restrictions upon publication of the work. In cases where code is central to the manuscript, we may require the code to be made available as a condition of publication. Authors are responsible for ensuring that the code is reusable and well documented. Please make any custom code available, either as part of your data deposition or as a supplementary file. Please add a sentence to your data availability statement regarding any code used in the study, e.g. "The code used in the analysis is available from Github [URL] and archived in Zenodo [DOI link]" Please review our guidelines at https://journals.plos.org/plosmedicine/s/materials-software-and-code-sharing and ensure that your code is shared in a way that follows best practice and facilitates reproducibility and reuse. Because Github depositions can be readily changed or deleted, we encourage you to make a permanent DOI'd copy (e.g. in Zenodo) and provide the URL.

**Response:** Thank you for this advice. This paper does not include any author-generated code. All analyses were conducted using SAS or publicly available R packages.

**Comment 6:** Please ensure that the study is reported according to the [XXXX] guideline and include the completed [XXXX] checklist as Supporting Information. When completing the checklist, please use section and paragraph numbers, rather than page numbers. Please add the following statement, or similar, to the Methods: "This study is reported as per [XXXX] guideline (S1 Checklist)."

**Response:** Thank you for this valuable suggestion. We have ensured that this study is reported in accordance with the CONSORT guideline. The completed CONSORT checklist has been added as Supporting Information (S1 Checklist). In addition, we have added the following statement to the Methods section (Page 7, Lines 173-174): “This study is reported as per the Consolidated Standards of Reporting Trials (CONSORT) guideline.”

**Comment 7:** Abstract: Please structure your abstract using the PLOS Medicine headings (Background, Methods and Findings, Conclusions). Please combine the Methods and Findings sections into one section.

**Response:** Thank you for this advice. We have structured the Abstract according to the PLOS Medicine headings and combined the Methods and Findings sections into one section.

**Comment 8:** At this stage, we ask that you include a short, non-technical Author Summary of your research to make findings accessible to a wide audience that includes both scientists and non-scientists. The Author Summary should immediately follow the Abstract in your revised manuscript. This text is subject to editorial change and should be distinct from the scientific abstract. Ideally each sub-heading should contain 2-3 single sentence, concise bullet points containing the most salient points from your study. In the final bullet point of 'What Do These Findings Mean?', please include the main limitations of the study in non-technical language. Please see our author guidelines for more information: https://journals.plos.org/plosmedicine/s/revising-your-manuscript#loc-author-summary.

**Response:** Thank you for this advice. We have added “Author Summary” statement (Page 3, Lines 81-109) as follows:

**Why Was This Study Done?**

- Many ST-segment elevation myocardial infarction (STEMI) patients present at non-percutaneous coronary intervention (PCI) capable hospitals and require transfer, a process that may prolong ischemic time and increase mortality.
- The large-scale BRIGHT-4 trial found that using bivalirudin instead of heparin, followed by a 2-4-hour post-PCI high-dose infusion, significantly lowered the risk of death or major bleeding within 30 days in patients with STEMI undergoing primary PCI.
- Current clinical practice lacks evidence on outcomes and optimal anticoagulation for transferred STEMI patients.

**What Did the Researchers Do and Find?**

- We conducted a *post hoc* analysis of the BRIGHT-4 trial, focusing on 5938 patients with STEMI undergoing primary PCI, all of whom had clear records of how they arrived at PCI-capable hospitals.
- Despite a 2-hour delay to reperfusion, patients who had to be transferred for primary PCI did not have worse outcomes after 30 days compared to those who arrived directly at PCI-capable hospitals.
- The need to transfer between hospitals did not reduce the benefits of using bivalirudin with a post-PCI high-dose infusion to lower the risk of death or major bleeding, compared to using heparin alone.

**What Do These Findings Mean?**

- This study emphasized that speed mattered most － timely PCI treatment was more important than how patients got to the hospital, whether they came directly or were transferred.
- The findings suggested that using bivalirudin instead of heparin during primary PCI might improve survival and offer better protection against major bleeding in STEMI patients, no matter how they reached the hospital.
- More research is needed to see how inter-hospital transfer affects the long-term prognosis of STEMI patients and to assess the benefits of bivalirudin over time.

**Comment 9:** Please express the main results with 95% CIs as well as p values. When reporting p values please report as p<0.001 and where higher as the exact p value p=0.002, for example. Throughout, suggest reporting statistical information as follows to improve clarity for the reader "22% (95% CI [13%,28%]; p</=)". Please be sure to define all numerical values at first use.

**Response:** Thank you for this advice. We are in compliance with it.

**Comment 10:**Please include page numbers and line numbers in the manuscript file. Use continuous line numbers (do not restart the numbering on each page).

**Response:** Thank you for this advice. We are in compliance with it.

**Comment 11:** Please cite the reference numbers in square brackets. Citations should precede punctuation.

**Response:** Thank you for this advice. We are in compliance with it.

**Comment 12:** Please provide titles and legends for all figures and tables (including those in Supporting Information files).

**Response:** Thank you for this advice. We are in compliance with it.

**Comment 13:** Please define all abbreviations used in each figure/table (including those in Supporting Information files).

**Response:** Thank you for this advice. We are in compliance with it.

**Comment 14:** Please consider avoiding the use of red and green in order to make your figure more accessible to those with color blindness.

**Response:** Thank you for this advice. We confirm that we did not use a red-and-green color combination in our figures. The colors used are either red and blue or green and blue, which are both colorblind-friendly and clearly distinguishable.

**Comment 15:** Please note that supplementary material will be posted as supplied by the authors. Therefore, please amend it according to the relevant comments outlined here.

**Response:** Thank you for this advice. We are in compliance with it.

**Comment 16:** Please cite your Supporting Information as outlined here: <https://journals.plos.org/plosmedicine/s/supporting-information>

**Response:** Thank you for this advice. We are in compliance with it.

**Comment 17:** PLOS uses the numbered citation (citation-sequence) method and first six authors, et al.

**Response:** Thank you for this advice. We are in compliance with it.

**Comment 18:** Please ensure that journal name abbreviations match those found in the National Center for Biotechnology Information (NCBI) databases (http://www.ncbi.nlm.nih.gov/nlmcatalog/journals), and are appropriately formatted and capitalised.

**Response:** Thank you for this advice. We are in compliance with it.

**Comment 19:** Where website addresses are cited, please include the complete URL and specify the date of access (e.g. [accessed: 12/06/2023]).

**Response:** Thank you for this advice. Upon reviewing the manuscript, we confirm that there are no website addresses or online resources cited.

**Comment 20:**Please also see https://journals.plos.org/plosmedicine/s/submission-guidelines#loc-references for further details on reference formatting.

**Response:** Thank you for this advice. We are in compliance with it.

**Comment 21:** PLOS Medicine requires that all trials be prospectively registered in one of registries recognized by WHO. Please ensure that study registration details are included in the Methods section.

**Response:** Thank you for this advice. We are in compliance with it.

**Comment 22:** Please structure the Methods section using the following sub-headings: Study design and participants, Randomization and masking, Procedures, Outcomes, Statistical analysis.

**Response:** Thank you for this advice. This study is a *post hoc* analysis of the BRIGHT-4 trial. The design of the BRIGHT-4 trial has been previously published in detail. Thus, we have integrated the details of randomization and masking, as well as procedures, into the "Study design and participants" section. Accordingly, we have structured the Methods section with the following sub-headings: Study design and participants, Clinical outcomes and definitions, and Statistical analysis.

**Comment 23:** The following outcomes measures [ADD DETAILS AS NEEDED OR DELETE BULLET POINT] appear to differ between the submitted manuscript and the protocol [and/or trial registry]. Please clarify and explain all discrepancies between the paper and protocol. If the outcomes were not prespecified in the protocol, please define them in the Methods (Outcomes section) as post hoc and explain why they were added. Post-hoc comparisons should be presented as hypothesis generating rather than conclusive.

**Response:** Thank you for your valuable advice. This study is a *post hoc* analysis of the BRIGHT-4 trial, rather than a pre-specified subgroup analysis. However, the statistical methods employed in this analysis are identical to those used for the pre-specified subgroups in the BRIGHT-4 trial. To ensure clarity, we have emphasized the nature of our analysis in the Methods section, and noted in the Limitations section that our results should be considered hypothesis-generating rather than conclusive inference.

**Comment 24:** Please ensure that all prespecified outcomes (primary, secondary, and exploratory) are listed in the Methods/Outcomes section and indicate whether there are outcomes that are not presented in the current report.

**Response:** Thank you for this advice. This study is a *post hoc* analysis of the BRIGHT-4 trial, rather than a pre-specified subgroup analysis. However, we applied the same statistical methods used for the pre-specified subgroups in the BRIGHT-4 trial. The study protocol and statistical analysis plan have been added as Supporting Information (S1 Text. Study protocol, S2 Text. Statistical analysis plan). We have clarified the exploratory nature of our analysis in the Methods section, and noted in the Limitations section that our results should be considered hypothesis-generating rather than conclusive inference.

**Comment 25:** Please specify the dates (Month Day, Year) during which study enrollment and follow up occurred.

**Response:** Thank you for this advice. We are in compliance with it.

**Comment 26:** Please include absolute numbers wherever you report percentages; eg, n/N (%)

**Response:** Thank you for this advice. We are in compliance with it.

**Comment 27:** Please present the safety data for the study including numbers of specific events and whether or not adverse events are thought to be related to treatment. AEs should be reported in the abstract, per CONSORT and CONSORT-Harms.

**Response:** Thank you for your comment. Bivalirudin and heparin are commonly used anticoagulants during the peri-procedural period of primary PCI in STEMI patients, and both are widely applied in clinical practice. The most common adverse event associated with these agents is an increased risk of bleeding, which has been clearly presented in the manuscript, as shown in Abstract and Table 3-4, S4-7 Tables.

**Comment 28:** Please complete the CONSORT checklist (https://www.equator-network.org/reporting-guidelines/consort/) and ensure that all components of CONSORT are present in the manuscript, including how randomization was performed, allocation concealment, blinding of intervention, definition of lost to follow-up, power statement. When completing the checklist, please use section and paragraph numbers, rather than page numbers.

**Response:** Thank you for this advice. The completed CONSORT checklist has been added as Supporting Information (S1 Checklist). In addition, we have added the following statement to the Methods section (Page 7, Lines 173-174): “This study is reported as per the Consolidated Standards of Reporting Trials (CONSORT) guideline.”

**Comment 29:** Please report your abstract according to CONSORT for abstracts, following the PLOS Medicine abstract structure (Background, Methods and Findings, Conclusions) https://www.equator-network.org/reporting-guidelines/consort-abstracts/

**Response:** Thank you for this advice. We are in compliance with it.

**Comment 30:** If your trial had to undergo important modifications in response to extenuating circumstances, please complete the CONSERVE-CONSORT checklist and provide in your Supporting Information; (https://www.equator-network.org/reporting-guidelines/guidelines-for-reporting-trial-protocols-and-completed-trials-modified-due-to-the-covid-19-pandemic-and-other-extenuating-circumstances-the-conserve-2021-statement/). When completing the checklist, please use section and paragraph numbers, rather than page numbers.

**Response:** Thank you for this advice. The CONSERVE-CONSORT checklist is not applicable to our research.

**Comment 31:** In keeping with our commitment to Open Science, please include the study protocol document and analysis plan (including any amendments) as Supporting Information to be published with the manuscript if accepted.

**Response:** Thank you for this advice. This study is a *post hoc* analysis of the BRIGHT-4 trial, rather than a pre-specified subgroup analysis. However, the statistical methods employed in this analysis are identical to those used for the pre-specified subgroups in the BRIGHT-4 trial. To ensure clarity, the study protocol and statistical analysis plan have been added as Supporting Information (S1 Text. Study protocol, S2 Text. Statistical analysis plan).

**Comment 32:** Please note that PLOS Medicine requires prospective, public registration of a data sharing plan (as part of mandatory clinical trials registration) for all clinical trials that began enrollment on or after January 1, 2019, in accordance with ICMJE requirements.

**Response:** Thank you for this advice. We are in compliance with it. We then have added “Data Availability” statement (Page 21, Lines 490-492) as follows: ”The authors are willing to share de-identified individual data with researchers who provide a methodologically sound proposal. Interested parties should contact the Clinical Research Data Center of the General Hospital of Northern Theatre Command via Lcyjsjzx@163.com.”

**Comment 33:** Please amend your detailed Financial Disclosure statement. This is published with the article, therefore should be completed in full sentences and contain the exact wording you wish to be published. i). State what role the funders took in the study. If the funders had no role in your study, please state: “The funders had no role in study design, data collection and analysis, decision to publish, or preparation of the manuscript.” ii). If any authors received a salary from any of your funders, please state which authors and which funders.

**Response:** Thank you for this advice. We have added the following statement to the Financial Disclosure section (Page 21, Lines 477-479): “The funders had no role in study design, data collection and analysis, decision to publish, or preparation of the manuscript. None of the authors received salary support from any of the funders.”

**Comment 34:** Please amend your detailed Financial Disclosure statement. This is published with the article, therefore should be completed in full sentences and contain the exact wording you wish to be published. i). State the initials, alongside each funding source, of each author to receive each grant.

**Response:** Thank you for this advice. We have added the initials of the authors alongside their respective funding sources as requested.

**Comment 35:** Please send a completed 'Competing Interests' statement, including any COIs declared by your co-authors. If you have no competing interests to declare, please state "The authors have declared that no competing interests exist". Otherwise please declare all competing interests beginning with the statement "I have read the journal's policy and the authors of this manuscript have the following competing interests:”

**Response:** Thank you for this advice. We have reviewed [PLOS Competing Interests Policy](http://journals.plos.org/plosmedicine/s/competing-interests) and are compliant with it. We have revised ‘Competing Interests’ statement (Page 20, Lines 460-461) as follows: ”I have read the journal's policy and the authors of this manuscript have the following competing interests: Dr. Stone has received speaker honoraria from Pulnovo, Medtronic, Amgen, Boehringer Ingelheim, Abiomed; has served as a consultant to CorFlow, Cardiomech, Robocath, Daiichi Sankyo, Ablative Solutions, Vectorious, Miracor, Apollo Therapeutics, Elucid Bio, Abbott, Cardiac Success, Occlutech, Millennia Biopharma, Remote Cardiac Enablement, Valfix, Zoll, HeartFlow, Shockwave, Impulse Dynamics, Adona Medical, Oxitope, HighLife, Elixir, Aria; and has equity/options from Cardiac Success, Ancora, Cagent, Applied Therapeutics, Biostar family of funds, SpectraWave, Orchestra Biomed, Aria, Valfix, Xenter. Dr. Stone’s employer, Mount Sinai Hospital, receives research grants from Shockwave, Biosense-Webster, Abbott, Abiomed, Bioventrix, Cardiovascular Systems Inc, Phillips, Vascular Dynamics, Pulnovo, V-wave and PCORI (via Weill Cornell Medical Center). The other authors have no disclosures.”

**Comment 36:** Thank you for your statement " The authors are willing to share deidentiffed individual data with researchers who provide a methodologically sound proposal. Interested parties should contact the corresponding author of this article via email. " Please note that the contact for accessing your data cannot be an author on the manuscript. Please therefore replace the current contact information with a third party contact, such as an institutional data access committee.

**Response:** Thank you for this advice. We have thoroughly read PLOS Data Availability Policy and are in compliance with it. We have added “Data Availability” statement (Page 21, Lines 490-492) as follows: ”The authors are willing to share de-identified individual data with researchers who provide a methodologically sound proposal. Interested parties should contact the Clinical Research Data Center of the General Hospital of Northern Theatre Command via Lcyjsjzx@163.com.”

**Comment 37:** Some material included in your submission may be copyrighted. According to PLOS’s copyright policy, authors who use figures or other material (e.g., graphics, clipart, maps) from another author or copyright holder must demonstrate or obtain permission to publish this material under the Creative Commons Attribution 4.0 International (CC BY 4.0) License used by PLOS journals. Please closely review the details of PLOS’s copyright requirements here: PLOS Licenses and Copyright. If you need to request permissions from a copyright holder, you may use PLOS's Copyright Content Permission form.

Please respond directly to this email and provide any known details concerning your material's license terms and permissions required for reuse, even if you have not yet obtained copyright permissions or are unsure of your material's copyright compatibility. Once you have responded and addressed all other outstanding technical requirements, you may resubmit your manuscript within Editorial Manager.

Potential Copyright Issues:

1) [Central Illustration.tif]: Please confirm whether you drew the images / clip-art within the figure panels by hand. If you did not draw the images, please provide (a) a link to the source of the images or icons and their license / terms of use; or (b) written permission from the copyright holder to publish the images or icons under our CC BY 4.0 license. Alternatively, you may replace the images with open source alternatives. See these open source resources you may use to replace images / clip-art:

- https://commons.wikimedia.org

- https://openclipart.org/

**Response:** Thank you for your valuable advice. We have removed the ambulance and heart images that could potentially infringe on copyright. The remaining content now only includes illustrations that we have created ourselves. Therefore, there should be no further concerns regarding copyright issues.
