# Supplementary material for: Analysis of inter-hospital transfer on clinical outcomes after primary percutaneous coronary intervention for ST-segment elevation myocardial infarction: A secondary analysis of the BRIGHT-4 trial
Source: PLoS Med. 2025 Jul 23;22(7):e1004679. doi: 10.1371/journal.pmed.1004679 (PMC12313069; doi:10.1371/journal.pmed.1004679)
Supplement: Attachment — Submitted filename: Response_to_reviewers_auresp_3.docx [file pmed.1004679.s016.docx]

**Submission ID: PMEDICINE-D-24-04173R3**

**Analysis of inter-hospital transfer on clinical outcomes after primary percutaneous coronary intervention for ST-segment elevation myocardial infarction: A secondary analysis of the BRIGHT-4 trial**

Dear Editors and Reviewers:

We sincerely appreciate the time and effort you have devoted to carefully reviewing our manuscript. Your thoughtful comments and valuable suggestions have greatly contributed to enhancing the quality of our work.

We have carefully considered each of your comments and have implemented revisions to address them effectively. In the following sections, we provide detailed, point-by-point responses to each comment, aiming to clearly demonstrate our commitment to enhancing the clarity, rigor, and overall impact of our research. Additionally, we have utilized the Track Changes feature in Microsoft Word to ensure that all modifications made during the revision process are clearly highlighted for your reference. Hope the corrections can meet the comments with approval.

We earnestly appreciate for your warm work, thank you very much again for your comments and suggestions. We greatly value your support and are happy to address any further questions or suggestions you may have.

Sincerely yours,

Yaling Han, MD, FACC, FESC, State Key Laboratory of Frigid Zone Cardiovascular Disease, Department of Cardiology, General Hospital of Northern Theatre Command, Shenyang 110016, China. E-mail: [hanyaling@163.net](mailto:hanyaling@163.net)

The corrections and answers in response to your comments are listed as follows:

**Answers to Editors**

**Comment 1:** Please remove the 'conclusions' subheading from the discussion. Please also remove any other subheadings from the discussion.

**Response:** Thank you for your guidance. We have removed the 'Conclusions' and 'Limitations' subheadings from the Discussion section. These sections are now introduced with the sentences (Page 16, Line 388)“This study also has some limitations.” and (Page 17, Line 401)“We concluded that...”, respectively.

**Comment 2:** In the last sentence of the Abstract Methods and Findings section, please describe the main limitation(s) of the study's methodology.

**Response:** Thank you for your advice. To address this issue, we have added the following statement in the last sentence of the Abstract Methods and Findings section (Page 2, Lines 66-68): “The main limitations of this study are that it is a *post hoc* analysis, and the long-term prognostic impact of transfer on STEMI patients requires further investigation.”

**Comment 3:** In the author summary, in the final bullet point of 'What Do These Findings Mean?', please include the main limitations of the study in non-technical language.

**Response:** Thank you for your advice. In response, we have added the following statement in the author summary (Page 4, Lines 111-113): “The generalizability of the present study is limited by not fully balancing the differences between patients who were transferred and those who arrived directly at the hospital, as well as by a relatively short follow-up period. ”

**Comment 4:** PLOS Medicine requires that the de-identified data underlying the specific results in a published article be made available, without restrictions on access, in a public repository or as Supporting Information at the time of article publication, provided it is legal and ethical to do so. Please see the policy at http://journals.plos.org/plosmedicine/s/data-availability and FAQs at http://journals.plos.org/plosmedicine/s/data-availability#loc-faqs-for-data-policy"

The Data Availability Statement (DAS) requires revision. For each data source used in your study:

If the data are owned by a third party but freely available upon request, please note this and state the owner of the data set and contact information for data requests (web or email address).

Note that a study author cannot be the contact person for the data. Please confirm that the listed email address is not that of a study author.

**Response:** We thank the reviewer for highlighting this important requirement. As stated in the Data Availability Statement, we are willing to share de-identified individual data with researchers who provide a methodologically sound proposal. Interested parties may request access to the data by contacting the Clinical Research Data Center of the General Hospital of Northern Theatre Command at Lcyjsjzx@163.com. We confirm that the contact email provided is not associated with any of study authors and is the official email address of the Clinical Research Data Center of the General Hospital of Northern Theatre Command.

**Comment 5:** Did your study have a prospective protocol or analysis plan? Please state this (either way) early in the Methods section.

a) If a prospective analysis plan (from your funding proposal, IRB or other ethics committee submission, study protocol, or other planning document written before analyzing the data) was used in designing the study, please include the relevant prospectively written document with your revised manuscript as a Supporting Information file to be published alongside your study, and cite it in the Methods section. A legend for this file should be included at the end of your manuscript.

b) If no such document exists, please make sure that the Methods section transparently describes when analyses were planned, and when/why any data-driven changes to analyses took place.

c) In either case, changes in the analysis-- including those made in response to peer review comments-- should be identified as such in the Methods section of the paper, with rationale.

**Response:** Thank you for this important suggestion. This study is a *post hoc* analysis of the BRIGHT-4 trial, rather than a pre-specified subgroup analysis. However, we applied the same statistical methods used for the pre-specified subgroups in the BRIGHT-4 trial to ensure methodological consistency and transparency. The original study protocol and statistical analysis plan (SAP) of the BRIGHT-4 trial have been uploaded as Supporting Information files (S2 Text. Study protocol, S3 Text. Statistical analysis plan). To address this issue, we have added the following statement to the Methods section (Page 7, Lines 175-176):” Further details regarding the original study protocol and statistical analysis plan are provided in the Supporting Information (S2 and S3 Texts).”

**Comment 6:** Please remove any language that implies causality and refer to associations instead.

**Response:** Thank you for this valuable suggestion. We have carefully reviewed the manuscript and revised all relevant sections to remove language that may imply causality. Throughout the text, we now refer to observed associations or relationships rather than causal effects.

**Comment 7:** Please confirm that your title complies with to PLOS Medicine's style. Your title must be nondeclarative and not a question. It should begin with main concept if possible. "Effect of" should be used only if causality can be inferred, i.e., for an RCT. Please place the study design ("A randomized controlled trial," "A retrospective study," "A modelling study," etc.) in the subtitle (ie, after a colon). Please see the requested revised title.

**Response:** Thank you for your advice. We have revised the title to comply with PLOS Medicine’s style requirements. The revised title is as follows:“ Analysis of inter-hospital transfer on clinical outcomes after primary percutaneous coronary intervention for ST-segment elevation myocardial infarction: A secondary analysis of the BRIGHT-4 trial”.

**Comment 8:** Please ensure that all abbreviations are defined at first use throughout the text.

**Response:** Thank you for your comment. We have checked the entire text and made sure that all abbreviations are now defined upon first use in each section of the manuscript.

**Comment 9:** Please define or remove BARC in the Abstract and explain onset-to-wire' for the general reader.

**Response:** Thank you for your advice. To address this issue, we have made the following revisions in the Abstract: we have supplemented the full definition of BARC as “Bleeding Academic Research Consortium” (Page 2, Lines 50-51) and clarified that “onset-to-wire” refers to “the time from symptom onset to wire crossing the infarct-related artery” (Page 2, Lines 59).

**Comment 10:** Please identify by name all of the IRBs that approved the protocol.

**Response:** Thank you for your advice. The study protocol was approved by the ethics committee of the General Hospital of Northern Theatre Command as well as at each participating center. In response, we have added S1 Text, which provides detailed information on the IRBs, and this section is referenced in the Methods (Page 7, Lines 172).

**S1 Text. Institutional Review Boards**

|  | **Institutional Review Boards (IRBs)** |
| --- | --- |
| 01 | General Hospital of Northern Theater Command, Shenyang, China |
| 02 | Kaifeng Central Hospital, Kaifeng, China |
| 03 | West China Hospital Sichuan University, Chengdu, China |
| 04 | Yuzhou City People’s Hospital, Yuzhou, China |
| 05 | Taian City Central Hospital, Taian, China |
| 06 | Tianjin Chest Hospital, Tianjin, China |
| 07 | Tangdu Hospital, Air Force Medical University, Xi’an, China |
| 08 | The Fifth People’s Hospital of Jinan, Jinan, China |
| 09 | The Second Hospital of Shenyang Medical College, Shenyang, China |
| 10 | Affillated Hospital of Qilu Medical University (The People’s Hospital of Xintai City), Xintai, China |
| 11 | Ganzhou People’s Hospital, Ganzhou, China |
| 12 | Bozhou People’s Hospital, Bozhou, China |
| 13 | Beijing Luhe Hospital of Capital Medical University, Beijing, China |
| 14 | Ganzhou Municipal Hospital, Ganzhou, China |
| 15 | Shangqiu First People’s Hospital, Shangqiu, China |
| 16 | Jiaozuo People’s Hospital, Jiaozuo, China |
| 17 | The First Affiliated Hospital of Anhui University of Science and Technology, Huannan, China |
| 18 | The Second Affiliated Hospital of Nanchang University, Nanchang, China |
| 19 | First Affiliated Hospital of Gannan Medical University, Ganzhou, China |
| 20 | The Third Affiliated Hospital of Xinxiang Medical University, Xinxiang, China |
| 21 | Yanbian University Hospital, Yanbian, China |
| 22 | The Fourth Affiliated Hospital of China Medical University, Shenyang, China |
| 23 | Heze Municipal Hospital, Heze, China |
| 24 | Huainan Cardiovascular Research Laboratory, Huannan, China |
| 25 | Xichang People’s Hospital, Xichang, China |
| 26 | The First Affiliated Hospital of Zhengzhou University, Zhengzhou, China |
| 27 | Peking University Third Hospital Yanqing Hospital, Beijing, China |
| 28 | The First Hospital of Lanzhou University, Lanzhou, China |
| 29 | Taihe County People’s Hospital, Taihe, China |
| 30 | Taizhou Central Hospital, Taizhou, China |
| 31 | The Affiliated Traditional Chinese Medicine Hospital of Southwest Medical University, Luzhou, China |
| 32 | Datong Third People’s Hospital, Datong, China |
| 33 | Luohe Central Hospital, Luohe, China |
| 34 | The Second Hospital of Jilin University, Changchun, China |
| 35 | China-Japan Union Hospital of Jilin University, Changchun, China |
| 36 | Fuyang People’s Hospital, Fuyang, China |
| 37 | The Affiliated Hospital of Guizhou Medical University, Guiyang, China |
| 38 | Meizhou Peopie’s Hospital, Meizhou, China |
| 39 | Qingdao Municipal Hospital, Qingdao, China |
| 40 | Affiliated Hospital of Zunyi Medical University, Zunyi, China |
| 41 | Bengbu Second People’s Hospital, Bengbu, China |
| 42 | Yueyang Central Hospital, Yueyang, China |
| 43 | The First Affiliated Hospital of Zhengzhou University, Zhengzhou, China |
| 44 | People’s Hospital of Pingchang County, Pingchang, China |
| 45 | Yunnan St.John’s Hospital, Kunming, China |
| 46 | Fuwai Central China Cardiovascular Hospital, Zhengzhou, China |
| 47 | The First Hospital of Liangshan, Liangshan, China |
| 48 | Central Hospital Affiliated to Shandong First Medical University, Jinan, China |
| 49 | Nanjing First Hospital, Nanjing, China |
| 50 | The First Affiliated Hospital of Nanchang University, Nanchang, China |
| 51 | Tianjin Medical University General Hospital, Tianjin, China |
| 52 | Hengshui Cardiovascular Hospital, Hengshui, China |
| 53 | Hospital Of Chengdu University of Traditional Chinese Medicine, Chengdu, China |
| 54 | Tongji Hospital, Tongji Medical College of HUST, Wuhan, China |
| 55 | Zaozhuang Central Hospital of Shandong Guoxin Healthcare Group, Zaozhuang, China |
| 56 | Shandong Second Provincial General Hospital, Jinan, China |
| 57 | Union Hospital, Tongji Medical College Of HUST, Wuhan, China |
| 58 | Xuzhou Central Hospital, Xuzhou, China |
| 59 | The First People’s Hospital of Ruzhou, Ruzhou, China |
| 60 | The Affiliated Hospital of Northwest University, Xi’an, China |
| 61 | Shanxi Cardiovascular Hospital, Xi’an, China |
| 62 | Suzhou Municipal Hospital of Anhui Province, Suzhou, China |
| 63 | Chongqing University Fuling Hospital, Chongqing, China |
| 64 | Heze Mudan People’s Hospital, Heze, China |
| 65 | The Fifth Affiliated Hospital of Xinjiang Medical University, Urumqi, China |
| 66 | Dalian No.3 People’s Hospital, Dalian, China |
| 67 | Linquan County People’s Hospital, Linquan, China |
| 68 | General Hospital of Fuxin Mining Industry Group of Liaoning Health Industry Group, Fuxin, China |
| 69 | General Hospital of Benxi Iron & Steel Industry Group of Liaoning Health Industry Group, Benxi, China |
| 70 | Yulin First People’s Hospital, Yulin, China |
| 71 | The First People’s Hospital of Xinmi, Xinmi, China |
| 72 | General Hospital of Fushun Mining Industry Group of Liaoning Health Industry Group, Fushun, China |
| 73 | The First Affiliated Hospital of Zhejiang Chinese Medical University, Hangzhou, China |
| 74 | The Second Affiliated Hospital of Xi’an Jiaotong University, Xi’an, China |
| 75 | No.2 People’s Hospital of Fuyang City, Fuyang, China |
| 76 | Xiangtan Central Hospital, Xiangtan, China |
| 77 | Loudi Central Hospital, Loudi, China |
| 78 | Jiangxi Provincial People’s Hospital, Nanchang, China |
| 79 | Wuhan Asia General Hospital, Wuhan, China |
| 80 | Jiangsu Taizhou People’s Hospital, Taizhou, China |
| 81 | Chifeng College Affiliated Hospital, Chifeng, China |
| 82 | Chengdu Second People’s Hospital, Chengdu, China |
| 83 | General Hospital of Tiefa Coal Industry Group of Liaoning Health Industry Group, Tiefa, China |
| 84 | Fushun Central Hospital, Fushun, China |
| 85 | Yan’an University Affiliated Hospital, Yan’an, China |
| 86 | Fourth Affiliated Hospital of Harbin Medical University, Harbin, China |
| 87 | Suixi Hospital, Suixi, China |

**Comment 11:** Please state in Methods any changes to the prespecified protocol for this analysis (if prespecified).

**Response:** Thank you for your comment. We confirm that there were no changes to the prespecified protocol for this analysis.

**Comment 12:** Please identify the central illustration in the file. Is this figure 2? Is this illustration copyrighted (which is not permitted)?

**Response:** Thank you for your comment. We have revised the figures accordingly: the original central illustration has been split into Fig 1 (The flowchart of the study population) and Fig 2 (Intervals for patients to a PCI facility), as indicated in the Results section. The former Figure 1 has been renumbered as Fig 3. All figures are original and do not involve any copyrighted material. Additionally, we confirm that all figures in our manuscript have been uploaded as individual TIF files with 300dpi resolution at resubmission, in accordance with the PLOS Medicine figure guidelines. We have used the PACE digital diagnostic tool for compliance verification.

**Comment 13:** Please state in the Abstract where the trial was conducted and if multicenter.

**Response:** Thank you for your advice. In response, we have revised the sentence in the Abstract from “The BRIGHT-4 trial demonstrated...” to “The multicenter BRIGHT-4 trial demonstrated...” (Page 2, Line 48)

**Comment 14:** Please state in the Abstract the specific objectives/outcomes of this study.

**Response:** Thank you for your valuable comment. In response, we have clarified the specific objective and outcomes of the study in the Abstract. The objective of the study is now stated as follows (Page 2, Lines 52-53):“This study aimed to assess the impact of inter-hospital transfer on clinical outcomes and the effectiveness of bivalirudin versus heparin in STEMI patients undergoing PCI.” And the clinical outcome has been specified as (Page 2, Lines 56-57):“The primary outcome was the composite of all-cause death or BARC types 3-5 bleeding occurring within 30 days. The secondary outcomes included stent thrombosis.”

**Comment 15:** Does the "primary outcome" (Abstract) refer to the BRIGHT-4 trial or to the primary analysis in this study. Please clarify whether these are primary and/or secondary outcomes:30-day all-cause mortality, major bleeding and stent thrombosis.

**Response:** Thank you for your important comment. To clarify, the primary and secondary outcomes reported in this study are identical to those of the BRIGHT-4 trial. As stated in the Methods section, the primary outcome was defined as the composite of 30-day all-cause death or BARC types 3-5 bleeding. The secondary outcomes included major adverse cardiac or cerebral events (MACCE, defined as the composite of all-cause death, (recurrent) myocardial infarction (MI), ischemia-driven target vessel revascularization (TVR), or stroke) and its components; stent thrombosis; BARC types 2-5 bleeding; the composite of all-cause death or BARC types 2-5 bleeding; acquired thrombocytopenia; and net adverse clinical events (NACE, the composite of MACCE or BARC types 3-5 bleeding). Additionally, we have added a description of both the primary and secondary outcomes in the Abstract, as follows: (Page 2, Lines 56-57) The primary outcome was the composite of all-cause death or BARC types 3-5 bleeding occurring within 30 days. The secondary outcomes included stent thrombosis.

**Comment 16:** Please use commas rather than dashes in confidence intervals.

**Response:** Thank you for your comment. We have revised all confidence intervals in the manuscript, Supporting Information and Fig 3 to use commas instead of dashes.

**Comment 17:** Please do not refer to patients as admissions (line 65). Please use direct admission patients.

**Response:** Thank you for your suggestion. We have changed “those for direct admissions” to “direct admission patients” at Line 70 (previously at Line 65).

**Comment 18:** Line 98: Please remove causal language and use associative language.

**Response:** Thank you for your valuable comment. In response, we have revised the sentence to use associative language throughout. The revised sentence reads: The association between bivalirudin use and lower rates of death or major bleeding appeared to be maintained regardless of the need for inter-hospital transfer.

**Comment 19:** Line 105: does this statement refer to findings in this manuscript or in the BRIGHT-4 trial? If the latter, please remove or revise.

**Response:** Thank you for your advice. The statement on Line 105 refers to the findings of the current study. To further avoid any implication of causality and align with the study’s observations, we have revised the statement as follows (Page 4, Lines 107-110): “When treating STEMI patients with primary PCI, bivalirudin could be a reasonable option no matter how the patient got to the hospital - even if they had to be moved from another facility. The connection between using bivalirudin and better outcomes (fewer deaths, less bleeding) stayed consistent either way.”

**Comment 20:** Please clarify for the general reader in the Abstract or Introduction what the high dose infusion is.

**Response:** Thank you for this helpful suggestion. In response, we have added the specific dose of the high-dose bivalirudin infusion (1.75 mg/kg/h) in the Abstract (Page 2, Line 49) and Introduction (Page 6, Line 153) sections to improve clarity for general readers.

**Comment 21:** Please state in the Abstract and Introduction that this is a post-hoc secondary analysis, and indicate in Methods if prespecified.

**Response:** Thank you for your suggestion. We have revised both the Abstract (Page 2, Line 69) and Introduction (Page 6, Line 155) to clearly state that this is a *post hoc* analysis, rather than a pre-specified subgroup analysis.

**Comment 22:** Line 287: provide a figure callout, and not to 'central illustration'.

**Response:** Thank you for your comment. We have revised the figures accordingly: the original central illustration has been split into Fig 1 (The flowchart of the study population) and Fig 2 (Intervals for patients to a PCI facility), as indicated in the Results section. The former Figure 1 has been renumbered as Fig 3. All figures are now properly called out in the text.

**Comment 23:** Please remove the Limitations subheading in the Discussion.

**Response:** Thank you for your advice. We have removed the Limitations subheadings from the Discussion section. The section is now introduced with the sentences (Page 16, Line 388)“This study also has some limitations.”

**Answers to Reviewers**

**Reviewer #2:**

**Comment 1:** I am satisfied with the authors responses and the resulting additional analyses. This is not the caliper I would choose by the way (too large), and I would probably opt for a maximum of 0.05. I don't need to see the paper again.

**Response:** Thank you for your valuable comment. In response, we have re-performed the PSM analysis using a caliper of 0.05 and updated the corresponding results accordingly. As shown below, Table S5 has been replaced with the updated data. A total of 1,882 well-balanced pairs were identified through the PSM analysis. The main findings remain consistent with those from the original analysis.

**S5 Table. Clinical outcomes at 30 days after propensity score matching**

|  | **Inter-hospital transfer (N=1882)** | **Direct admission  (N=1882)** | **HR (95%CI)** | ***P* Value** |
| --- | --- | --- | --- | --- |
| Primary outcome: All-cause death or BARC types 3-5 bleeding | 67 (3.6%) | 65 (3.5%) | 1.03 (0.73, 1.45) | 0.87 |
| Death from any cause | 63 (3.3%) | 62 (3.3%) | 1.01 (0.71, 1.44) | 0.94 |
| From cardiovascular causes | 60 (3.2%) | 59 (3.1%) | 1.01 (0.71, 1.45) | 0.94 |
| BARC types 3-5 bleeding | 9 (0.5%) | 7 (0.4%) | 1.29 (0.48, 3.45) | 0.62 |
| Reinfarction | 16 (0.9%) | 8 (0.4%) | 2.00 (0.86, 4.68) | 0.11 |
| Ischemia-driven TVR | 8 (0.4%) | 8 (0.4%) | 1.00 (0.38, 2.66) | >0.999 |
| Stroke | 10 (0.5%) | 10 (0.5%) | 1.00 (0.42, 2.40) | >0.999 |
| Stent thrombosis | 16 (0.9%) | 11 (0.6%) | 1.46 (0.68, 3.14) | 0.34 |
| Acute (<24 hours) | 7 (0.4%) | 4 (0.2%) | 1.75 (0.51, 5.98) | 0.37 |
| Subacute (1-30 days) | 9 (0.5%) | 7 (0.4%) | 1.29 (0.48, 3.46) | 0.62 |
| MACCE* | 86 (4.6%) | 83 (4.4%) | 1.03 (0.77, 1.40) | 0.83 |
| BARC types 2-5 bleeding | 32 (1.7%) | 44 (2.3%) | 0.72 (0.46, 1.14) | 0.16 |
| All-cause death or BARC types 2-5 bleeding | 89 (4.7%) | 99 (5.3%) | 0.89 (0.67, 1.19) | 0.44 |
| Acquired thrombocytopenia† | 85 (4.5%) | 63 (3.3%) | 1.36 (0.98, 1.88) | 0.07 |
| NACE‡ | 88 (4.7%) | 85 (4.5%) | 1.03 (0.77, 1.39) | 0.83 |

Event rates are number of events (Kaplan-Meier estimated percentages). MACCE, Major adverse cardiac or cerebral events. NACE, Net adverse clinical events. *MACCE includes all-cause death, myocardial infarction, ischemia-driven target vessel revascularization, or stroke. †Defined as nadir platelet count of <150×10^9^ cells/L after the index procedure in patients in whom the baseline platelet count was ≥150×10^9^ cells/L. ‡ NACE includes MACCE or BARC types 3-5 bleeding.
